# Rixosomal RNA degradation contributes to silencing of Polycomb target genes

**DOI:** 10.1038/s41586-022-04598-0

**Published:** 2022-03-30

**Authors:** Haining Zhou, Chad B. Stein, Tiasha A. Shafiq, Gergana Shipkovenska, Marian Kalocsay, Joao A. Paulo, Jiuchun Zhang, Zhenhua Luo, Steven P. Gygi, Karen Adelman, Danesh Moazed

**Affiliations:** 1 Howard Hughes Medical Institute, Department of Cell Biology, Blavatnik Institute, Harvard Medical School, Boston, MA, USA.; 2 Department of Biological Chemistry and Molecular Pharmacology, Blavatnik Institute, Harvard Medical School, Boston, MA, USA.; 3 Department of Cell Biology, Blavatnik Institute, Harvard Medical School, Boston, MA, USA.; 4 Initiative for Genome Editing and Neurodegeneration, Department of Cell Biology, Blavatnik Institute, Harvard Medical School, Boston, MA, USA.; 5 Precision Medicine Institute, the First Affiliated Hospital, Sun Yat-sen University, Guangzhou, China.

## Abstract

Polycomb repressive complexes 1 and 2 (PRC1 and PRC2) are histone-modifying and -binding complexes that mediate the formation of facultative heterochromatin and are required for silencing of developmental genes and maintenance of cell fate^1-3^. Multiple pathways of RNA decay work together to establish and maintain heterochromatin in fission yeast, including a recently identified role for a conserved RNA-degradation complex known as the rixosome or RIX1 complex^4-6^. Whether RNA degradation also has a role in the stability of mammalian heterochromatin remains unknown. Here we show that the rixosome contributes to silencing of many Polycomb targets in human cells. The rixosome associates with human PRC complexes and is enriched at promoters of Polycomb target genes. Depletion of either the rixosome or Polycomb results in accumulation of paused and elongating RNA polymerase at Polycomb target genes. We identify point mutations in the RING1B subunit of PRC1 that disrupt the interaction between PRC1 and the rixosome and result in diminished silencing, suggesting that direct recruitment of the rixosome to chromatin is required for silencing. Finally, we show that the RNA endonuclease and kinase activities of the rixosome and the downstream XRN2 exoribonuclease, which degrades RNAs with 5′ monophosphate groups generated by the rixosome, are required for silencing. Our findings suggest that rixosomal degradation of nascent RNA is conserved from fission yeast to human, with a primary role in RNA degradation at facultative heterochromatin in human cells.

The Polycomb group proteins have central roles in silencing of cell type-specific and growth-related control genes and their loss is associated with developmental abnormalities and cancer^1-3^. Two major Polycomb complexes with histone-modifying and -binding activities have been identified. In the canonical PRC1 (cPRC1) complex, the RING1A or RING1B protein associate with PCGF2 or PCGF4, PHC, and chromobox (CBX) proteins^7^. RING1A and RING1B are RING finger E3 ubiquitin ligases that form the catalytic core of PRC1 complexes and mediate the mono-ubiquitination of histone H2A lysine 119^8,9^ (H2AK119ub1). The PRC2 complex, consisting of EED, SUZ12, RBBP4 or RBBP7, and the EZH1 or EZH2 methyltransferases, methylates histone H3 lysine 27^10-13^ (H3K27). In addition to cPRC1, variant PRC1 (vPRC1) complexes, which lack CBX proteins and along with RING1A or B contain PCGF1, 3, 5 or 6 and RYBP or YAF2^14^, have been identified. Each PRC complex can recognize the modification it catalyses as well as the one catalysed by the other complex. Thus H3K27 trimethylation (H3K27me3) is recognized by the EED subunit of PRC2 itself and the CBX subunit of cPRC1s^15^, and H2AK119ub1 is recognized by RYBP–vPRC1 and PRC2 accessory subunits JARID2 and AEBP2^16-18^. This crosstalk creates reinforcing positive-feedback loops that may promote the epigenetic inheritance of silencing^2,3^.

H2AK119ub1 has a key role in initiating the cascade of modifications that lead to the formation of Polycomb domains. The direct recruitment of vPRC1 to DNA and subsequent ubiquitination of H2AK119 lead to the recruitment of PRC2, deposition of H3K27me3 and cPRC1 binding^19-21^. Notably, the RING1B and CBX2 subunits of PRC1 can mediate chromatin compaction in vitro and in vivo^22,23^, and CBX2 in particular can mediate liquid–liquid phase separation^24-29^. The mechanism of silencing has therefore been proposed to involve the exclusion of RNA polymerase II (PolII) via compaction or condensate formation^1,24-29^. However, in mouse embryonic stem cells, vPRC1 complexes lacking chromatin compaction activity contribute to silencing largely independently of cPRC1^30,31^. Previous studies also provide evidence for the presence of the general transcription machinery and PolII at promoters of Polycomb-repressed genes^1,32,33^, suggesting that mechanisms beyond chromatin compaction contribute to Polycomb silencing.

The rixosome is a highly conserved and essential multienzyme complex whose major role is in ribosomal RNA (rRNA) processing and ribosome biogenesis^6^. It contains an endonuclease subunit (human LAS1L), which cleaves within the rRNA internal transcribed spacer 2 and generates a precursor with a 5′-OH group. The polynucleotide kinase subunit (human NOL9) of the complex then phosphorylates the precursor in a step that is required for XRN2-mediated trimming and the generation of mature 26S rRNA. In the fission yeast *Schizosaccharomyces pombe*, the rixosome associates with heterochromatin and is required for the spreading of histone H3 lysine 9 (H3K9) methylation into actively transcribed regions and epigenetic inheritance of heterochromatin^4,34^. To test whether the human rixosome has similar roles in heterochromatin regulation, we purified the complex from human cells and analysed its composition by mass spectrometry. We found that the rixosome associates with the human PRC1 and PRC2 complexes and is recruited to Polycomb target genes, where it promotes degradation of nascent RNA and release of PolII.

## Rixosome association with Polycomb

**Extended Data Fig. 1 ∣:**
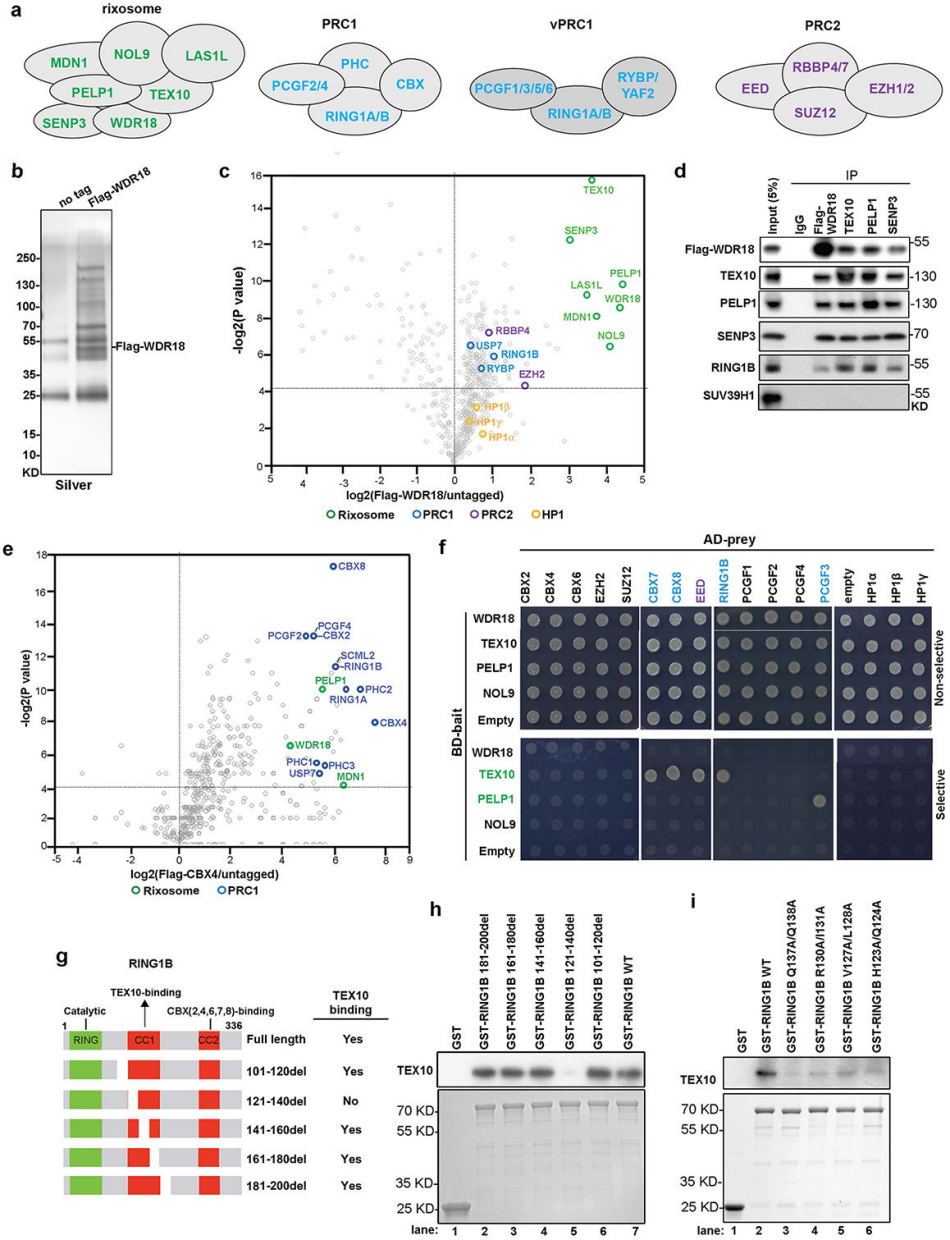
Physical association of the rixosome and Polycomb complexes. **a**, Diagrams showing the composition of the rixosome and Polycomb complexes. **b**, Silver-stained gel of Flag immunoprecipitations from untagged (no tag) and Flag-WDR18 HEK293FT cells. **c**, Volcano plot of TMT mass spectrometry results showing log2-fold changes in proteins enrichment in Flag immunoprecipitations from Flag-WDR18 versus untagged cells from two independent experiments. *p* values calculated by two-sided student’s t test. Rixosome, PRC1, PCR2, and HP1 proteins are highlighted in green, blue, magenta, and yellow, respectively. **d**, Immunoprecipitations (IP) showing the association of RING1B with rixosome subunits PELP1, TEX10, SENP3 and Flag-WDR18 in HEK293FT cells. **e**, Volcano plot of mass spectrometry results showing log2-fold changes in proteins enrichment in Flag immunoprecipitations from Flag-CBX4 versus untagged cells from two independent experiments. *p* values calculated by two-sided student’s t test. Rixosome and PRC1 subunits are highlighted in green and blue, respectively. **f**, Yeast two-hybrid assays. Yeast cells transformed with the indicated plasmids were plated onto double dropout (Non-selective) (SC-Trp, -Leu) or quadruple dropout (Selective)(SC-Trp, -Leu, -His, -Ade) medium. AD, Activation Domain; BD, Binding Domain. **g**, Diagram of the RING1B protein and the binding activities of its indicated truncations. CC1 and CC2, coiled-coil domains 1 and 2. **h, i**, Pull-down assays using bacterially expressed and purified TEX10 proteins and the indicated bead-immobilized GST or GST-fusion RING1B WT or mutant proteins. TEX10 detected by immunoblotting using an anti-TEX10 antibody. GST-tagged proteins were stained with Coomassie. The assays were performed three time independently with similar results.

**Fig. 1 ∣:**
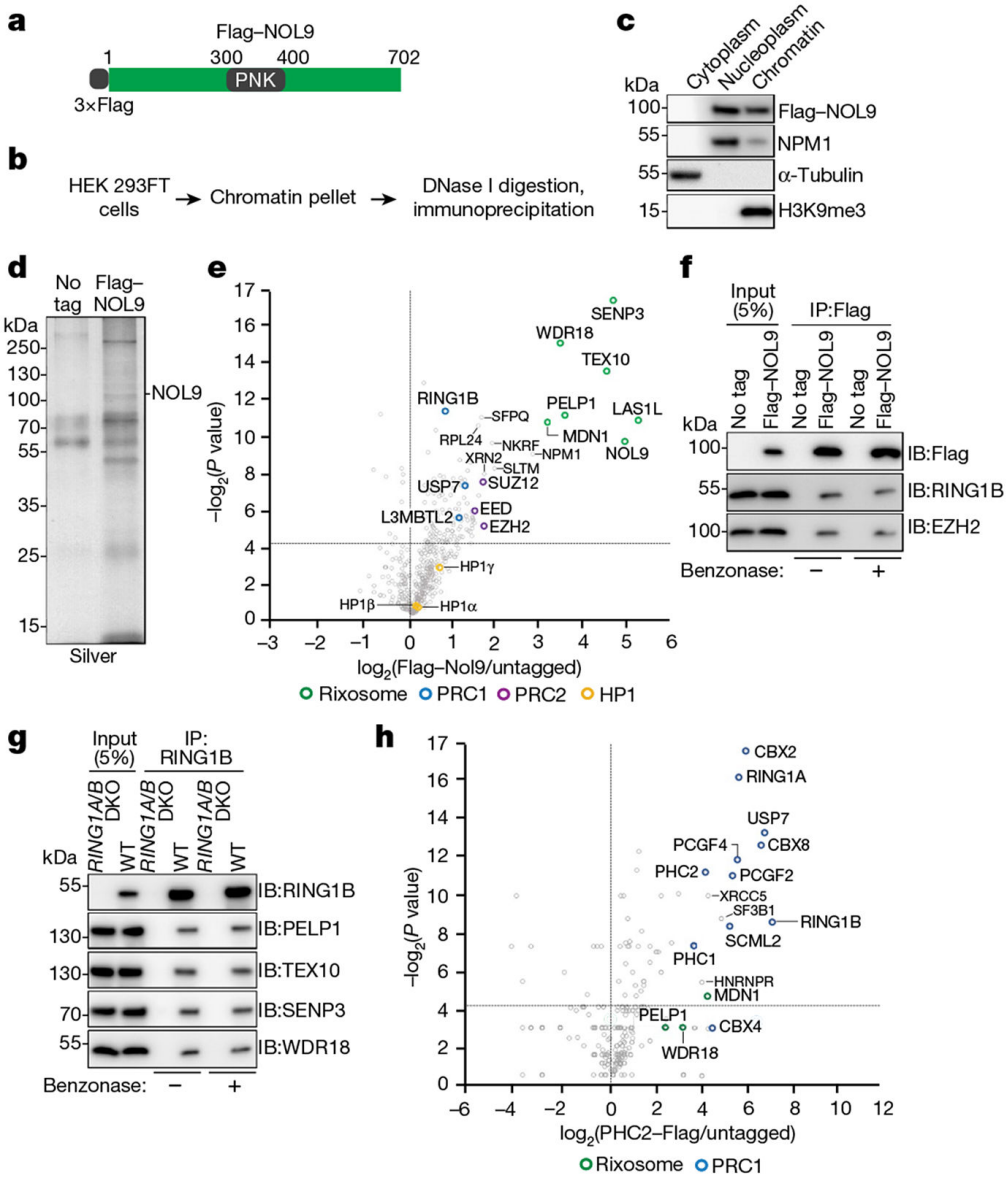
The rixosome interacts with the PRC1 and PRC2 complexes. **a,** Genomic tagging of endogenous *NOL9* with 3×Flag. **b,** Experimental design for protein immunoprecipitation from a chromatin fraction. **c,** Western blots showing fractionation of HEK 293FT cells. **d,** Silver-stained gel of Flag immunoprecipitations from wild-type and Flag–NOL9-expressing HEK 293FT cells. **e,** Volcano plot displaying results of tandem-mass-tag mass spectrometry of proteins enriched in Flag immunoprecipitations from Flag–NOL9-expressing cells relative to untagged cells from two independent experiments. *P* values calculated by two-sided *t*-test. Subunits of the rixosome, PRC1, PCR2 and H3K9me3-associated HP1 proteins are highlighted. **f,** Immunoprecipitations (IP) showing the association of RING1B and EZH2 with Flag–NOL9 in HEK 293FT cells with or without benzonase treatment. IB, immunoblot. **g,** Immunoprecipitations showing the association of RING1B with rixosome subunits PELP1, TEX10, SENP3 and WDR18 in HEK 293FT cells with or without benzonase treatment. **h,** Volcano plot displaying mass spectrometry results of proteins enriched in Flag immunoprecipitations from cells expressing Flag–PHC2 relative to untagged cells from two independent experiments. *P* values calculated by two-sided *t*-test. Subunits of the rixosome, PRC1, PCR2 and selected other proteins are highlighted.

**Fig. 2 ∣:**
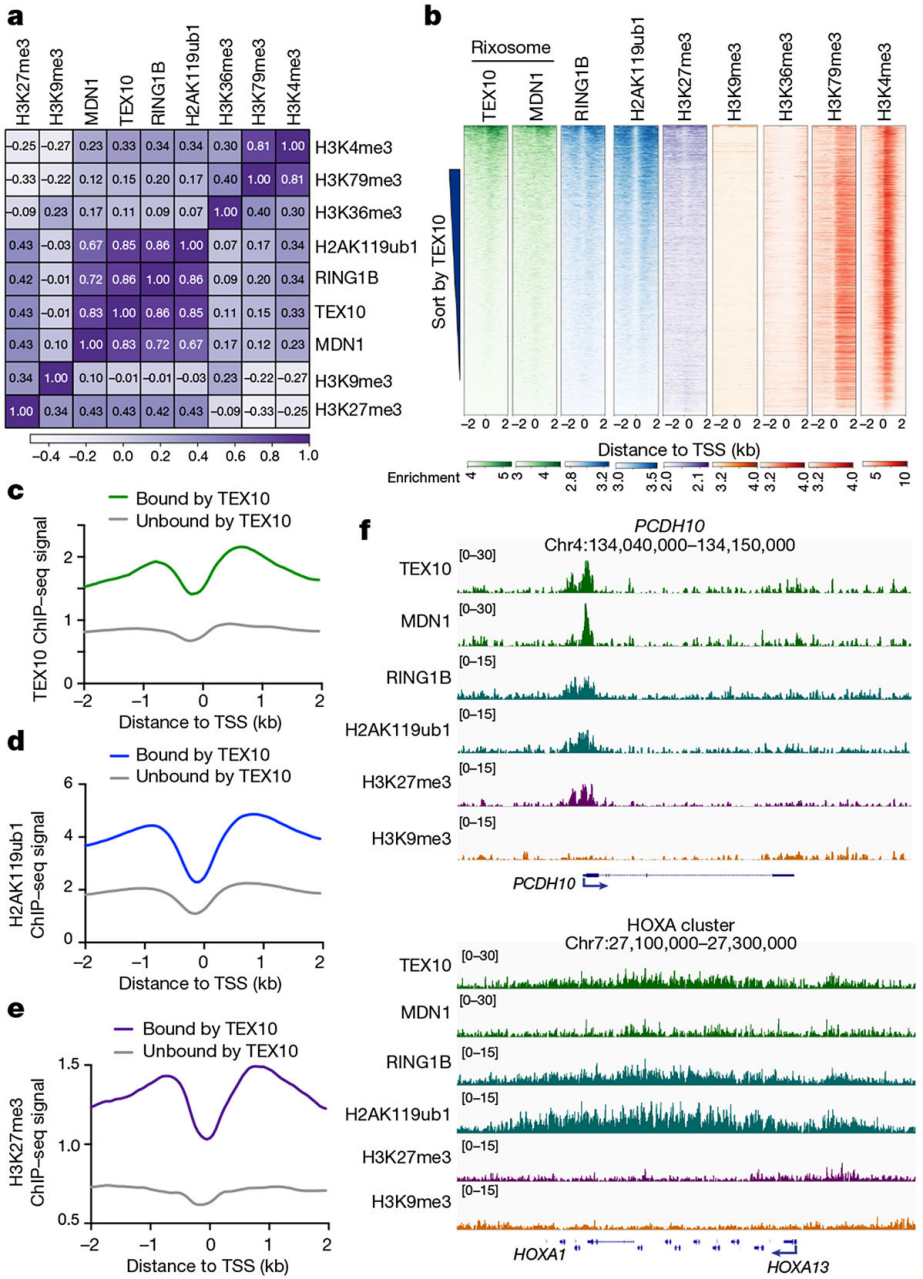
The rixosome localizes to transcription start sites with high PRC1 and PRC2 occupancy. **a**, Matrix depicting Spearman correlation coefficients between ChIP–seq datasets in HEK 293FT cells, calculated using summed read counts ±2 kb from all annotated gene TSSs (hg19). **b**, Heatmap representations of ChIP–seq of TEX10, MDN1, RING1B and histone modifications (H2AK119ub1, H3K4me3, H3K27me3, H3K9me3, H3K36me3, H3K79me3 and H3K4me3). Rank order is from highest to lowest TEX10 signal. log_2_ enrichment was normalized to reads per genome coverage. Read counts per gene were averaged in 50-nucleotide (nt) bins. **c–e**, Average distribution of TEX10 (**c**), H2AK119ub1 (**d**) and H3K27me3 (**e**) ChIP–seq reads at TEX10-bound genes (*n* = 7,827) versus TEX10-unbound genes (*n* = 13,177). Read counts per gene were summed in 50-nt bins. All three factors are significantly enriched at TEX10-bound genes, *P* < 2.2 × 10^−16^ for TEX10 (**c**), *P* < 2.2 × 10^−16^ for H2AK119ub1 (**d**), *P* < 2.2 × 10^−16^ for H3K27me3 (**e**); two-tailed Mann–Whitney test, using summed reads in the window ±2 kb from TSS. See Extended Data Fig. 2c, d for other modifications. **f**, Genomic snapshots of ChIP–seq reads for the indicated experiments at the Polycomb target *PCDH10* gene and HOXA cluster. log_2_ enrichment levels were normalized to reads per genome coverage.

We used CRISPR–Cas9 genome editing in human embryonic kidney (HEK 293FT) cells to modify the endogenous copies of rixosome genes *NOL9* and *WDR18* to express 3×Flag–NOL9 and 3×Flag–WDR18 (Fig. 1a, Extended Data Fig. 1a). NOL9 and WDR18 are the human orthologues of the Grc3 and Crb3 subunits of the fission yeast rixosome, mutations of which disrupt heterochromatin maintenance^4^. As the rixosome also has an essential role in rRNA processing and ribosome biogenesis in nucleoli^6^, we used a fractionation protocol to enrich for chromatin-bound, rather than nucleolar, rixosomes (Fig. 1b, c). We immunopurified 3×Flag–NOL9 and 3×Flag–WDR18 proteins (Fig. 1d, Extended Data Fig. 1b) and performed tandem-mass-tag mass spectrometry analysis of immunoprecipitates, which identified all seven known subunits of the rixosome—NOL9, WDR18, LAS1L, MDN1, PELP1, TEX10 and SENP3—in 3×Flag–NOL9 purifications (Fig. 1e, Extended Data Fig. 1a, Supplementary Table 7). In addition, subunits of PRC1 (RING1B), vPRC1.6 (L3MBTL2), PRC2 (EZH2, EED and SUZ12), and the PRC1-interacting ubiquitin protease (USP7) co-purified with 3×Flag–NOL9, but at lower efficiency than core rixosome components (Fig. 1e). Similarly, 3×Flag–WDR18 immunoprecipitations contained core rixosome, PRC1 (RING1B, RYBP and USP7), and PRC2 (EZH2 and RBBP4) subunits (Extended Data Fig. 1c). Consistent with these results, a previous study also identified rixosome subunits in immunoprecipitations of two different vPRC1 subunits^35^ and a proteome-wide immunoprecipitation and mass spectrometry study found an association between CBX4 and multiple rixosome subunits^36^. In addition, both rixosome and PRC1 subunits were identified in purifications of CHTOP, a human chromatin-associated protein^37^. In contrast to fission yeast^4,34^, H3K9me-associated HP1α, HP1β and HP1γ, were not significantly enriched in human rixosome purifications (Fig. 1e, Extended Data Fig. 1c), suggesting that in human cells the rixosome associates with heterochromatin modified with H2AK119ub1 and/or H3K27me3, rather than H3K9me3. We examined the above associations using immunoprecipitation and western blotting and found that (1) RING1B and EZH2 co-immunoprecipitated with Flag–NOL9 (Fig. 1f); (2) PELP1, NOL9, SENP3 and WDR18 rixosome subunits co-immunoprecipitated with RING1B (Fig. 1g); and (3) RING1B, but not SUV39H1, co-immunoprecipitated with several subunits of the rixosome (Extended Data Fig. 1d). These rixosome–Polycomb interactions were not sensitive to treatment with benzonase, suggesting that they occurred independently of RNA or DNA (Fig. 1f, g). In addition, mass spectrometry analysis of endogenously tagged and immunopurified PHC2–Flag and Flag–CBX4 showed that in addition to all of the subunits of cPRC1, the immunoprecipitates were enriched for the MDN1, WDR18 and PELP1 subunits of the rixosome (Fig. 1h, Extended Data Fig. 1e). These results demonstrate that the rixosome interacts physically with Polycomb complexes.

We next carried out yeast two-hybrid (Y2H) assays to identify potential direct rixosome–Polycomb interactions. These assays suggested that the rixosome subunit TEX10 interacts with CBX7, CBX8, EED and RING1B, and that PELP1 interacts with PCGF3 (Extended Data Fig. 1f). Consistent with the immunoprecipitation–mass spectrometry results, we observed no interactions between HP1 proteins and any of the rixosome subunits that we tested (Extended Data Fig. 1f). Therefore, in support of the biochemical data, the Y2H assays demonstrate interactions between the rixosome and PRC subunits.

As both immunoprecipitation–mass spectrometry and immunoprecipitation–western blotting identified RING1B as a rixosome-associated protein, and RING1B interacts with TEX10 in Y2H assays, we tested whether bacterially expressed and purified glutathione *S*-transferase (GST)–RING1B and TEX10 interacted in a pull-down assay. As shown in Extended Data Fig. 1g and Extended Data Fig. 1h, lane 7, full-length GST–RING1B, but not GST alone, pulled down TEX10. This interaction was greatly diminished upon deletion of amino acids 121–140 in the coiled-coil domain 1 (CC1) of RING1B but was not affected by several other RING1B deletions (Extended Data Fig. 1h, compare lane 5 with other lanes; summarized in Extended Data Fig. 1g). Furthermore, several amino acid substitutions within this domain, which did not affect RING1B expression, abolished the interaction of GST–RING1B with TEX10 (Extended Data Fig. 1i). Together, these results identify RING1B–TEX10 as a direct contact point between the rixosome and PRC1.

## Rixosome and Polycomb co-localization

**Extended Data Fig. 2 ∣:**
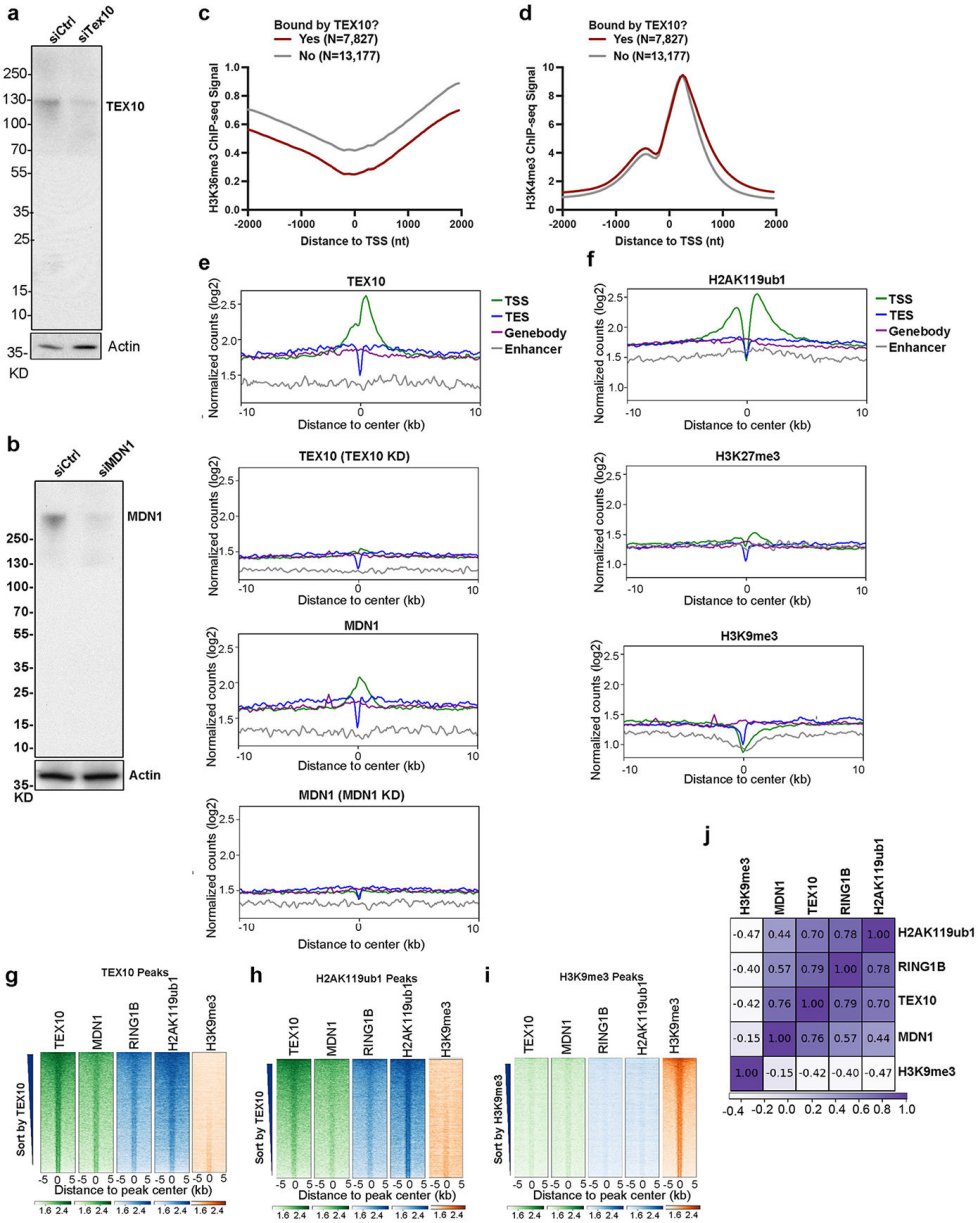
The rixosome is preferentially enriched at promoter regions. **a, b**, Immunoblot validation of siRNA-mediated TEX10 **(a)** and MDN1 **(b)** knockdowns. **c, d**, Average distribution of the indicated ChIP-seq reads at the TEX10-bound genes (n = 7,827) versus TEX-10-unbound genes (n = 13,177). Read counts per gene were averaged in 50-nt bins, using summed reads in the window +/−1kb from TSS. **e**, Average distribution of TEX10 and MDN1 ChIP-seq signal is shown relative to all annotated (hg19) transcription start sites (TSS), transcription termination sites (TES), gene bodies, and enhancers. Enrichment levels (log2) were normalized with Reads Per Genome Coverage. Read counts per gene were summed in 50-nt bins. **f**, Average distribution of H2AK119ub1, H3K27me3, and H3K9me3 ChIP-seq signal is shown relative to all annotated (hg19) transcription start sites (TSS), transcription termination sites (TES), gene bodies, and enhancers. Enrichment levels (log2) were normalized with Reads Per Genome Coverage. Read counts per gene were summed in 50-nt bins. **g**, Heatmap representations of ChIP-seq of TEX10, MDN1, RING1B and histone modifications H2AK119ub1 and H3K9me3 at TEX10 peak regions. Rank order is from most to least TEX10 signal. Enrichment levels (log2) were normalized with Reads Per Genome Coverage. Read counts per peak region were summed in 50-nt bins. **h**, Heatmap representations of ChIP-seq of TEX10, MDN1, RING1B and histone modifications H2AK119ub1 and H3K9me3 at H2AK119ub1 peak regions. Rank order is from most to least TEX10 signal. Enrichment levels (log2) were normalized with Reads Per Genome Coverage. Read counts per peak region were summed in 50-nt bins. **i**, Heatmap representations of ChIP-seq of TEX10, MDN1, RING1B and histone modifications H2AK119ub1 and H3K9me3 at H3K9me3 peak regions. Rank order is from most to least H3K9me3 signal. Enrichment levels (log2) were normalized with Reads Per Genome Coverage. Read counts per peak region were summed in 50-nt bins. **j**, Matrix depicting Spearman correlation coefficients between ChIP-seq datasets in HEK293 cells, calculated using read counts in all the genomic loci from **e–g**.

To examine the genome-wide localization of the rixosome in human cells, we carried out chromatin immunoprecipitation followed by high-throughput sequencing (ChIP–seq) in HEK 293FT cells using antibodies that recognize the TEX10 and MDN1 subunits of the rixosome. To control for antibody specificity, we performed ChIP–seq on cells treated with either control, TEX10-specific or MDN1-specific small interfering RNA (siRNA) (Extended Data Fig. 2a, b). Correlation analysis with ChIP–seq signals for Polycomb-catalysed histone modifications and RING1B showed highly correlated TEX10 (*r* = 0.85) and MDN1 (*r* = 0.67) colocalization with H2AK119ub1, high correlation for TEX10 colocalization with H3K27me3 (*r* = 0.43), and very high correlation between TEX10 (0.86) and MDN1 (0.72) colocalization with RING1B (Fig. 2a). For comparison, the correlation between H2AK119ub1 and H3K27me3 (*r* = 0.43) in these datasets was in a similar range (Fig. 2a). Consistently, heatmap analysis at all annotated transcription start sites (TSSs) showed similar enrichment patterns for TEX10, MDN1, RING1B, H2AK119ub1 and H3K27me3, but not H3K9me3 or H3K36me3, when we rank ordered genes by their TEX10 signal (Fig. 2b). TEX10-occupied genes also tended to exhibit H3K4me3, suggesting the presence of the rixosome at loci with engaged PolII, including bivalent Polycomb domains (containing both H3K27me3 and H3K4me3) (Fig. 2b). We then rigorously defined a set of TEX10-bound genes (Fig. 2c, *n* = 7,827) and compared with similarly active TEX10-unbound genes (*n* = 13,177), as described in Methods. We observed significant enrichment of H2AK119ub1 and H3K27me3 at TEX10-bound versus TEX10-unbound genes (Fig. 2d, e). By contrast, TEX10-bound genes were depleted of H3K36me3, whereas the TEX10-bound and unbound genes displayed similar enrichment for H3K4me3 (Extended Data Fig. 2c, d). Furthermore, TEX10 and MDN1 were enriched at TSSs (Extended Data Fig. 2e), as has been previously described for H2AK119ub1 and H3K27me3^38^ (Extended Data Fig. 2f). When we repeated the co-occupancy analysis using peak calling, rather than enrichment relative to TSSs, we found that both RING1B and H2AK119ub1, but not H3K9me3, were enriched at TEX10- and MDN1-bound genomic regions (Extended Data Fig. 2g-j). At the single-gene level, genome browser snapshots of TEX10 and MDN1 ChIP–seq reads at the *PCDH10* gene showed co-enrichment with H2AK119ub1 and H3K27me3, but not H3K9me3, whereas the HOXA cluster was enriched for the rixosome subunits and H2AK119ub1 (Fig. 2f).

**Extended Data Fig. 3 ∣:**
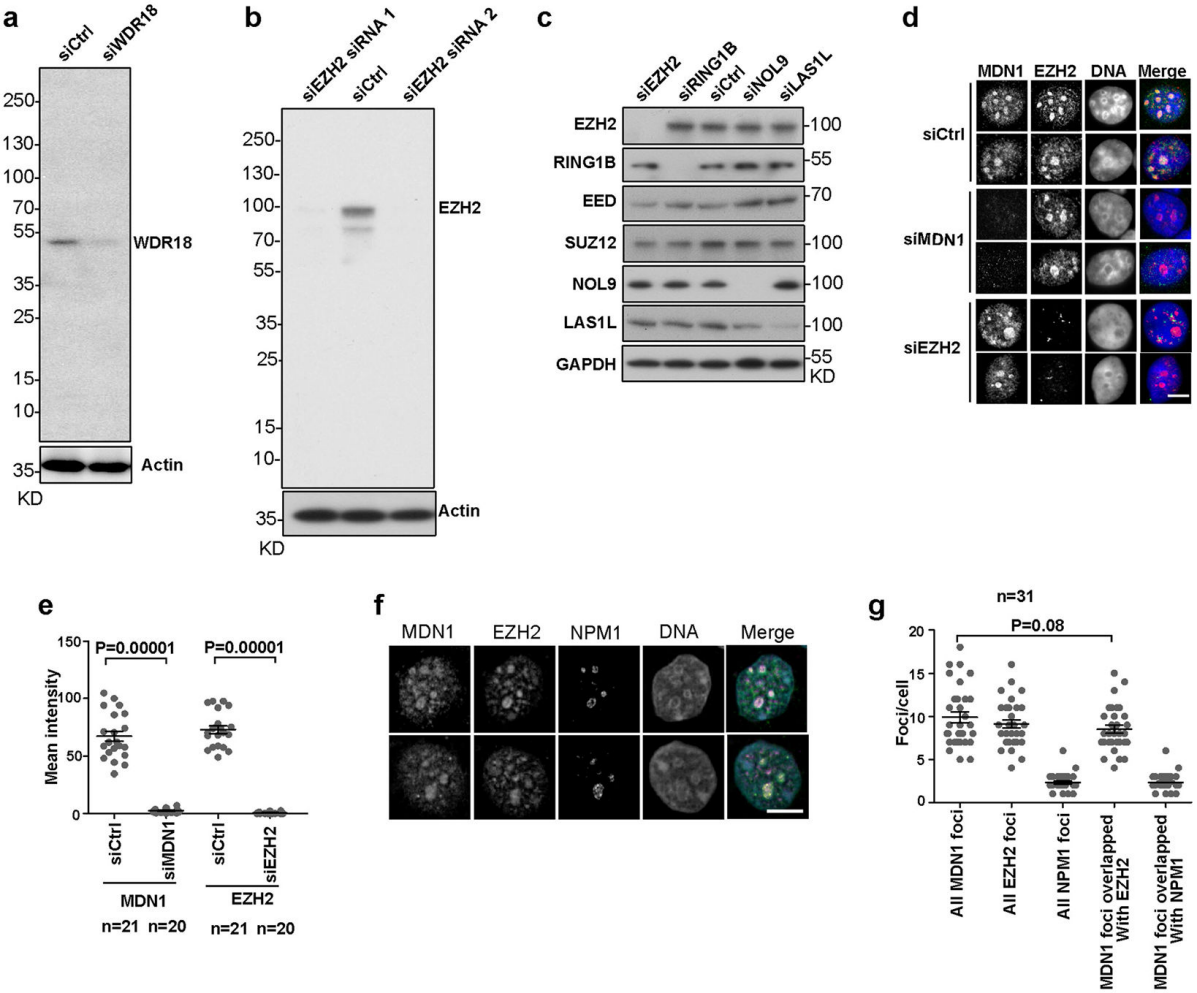
Colocalization of the rixosome with Polycomb bodies. **a, b**, Immunoblot validation of siRNA-mediated WDR18 **(a)** and EZH2 **(b)** knockdowns. **c**, Validation of siRNA knockdowns (48 h after transfection) of rixosome subunits, RING1B, and EZH2. **d**, Immunofluorescence colocalization of rixosome subunits MDN1 with EZH2-stained Polycomb bodies in cells treated with the indicated siRNA. DNA was stained with DAPI (blue). Scale bar, 5 μm. **e**, Quantification of MDN1 and EZH2 fluorescence intensity in **d**. *p* values are from two-sided student’s t-tests. Data are presented as mean values +/− SEM**. f**, Immunofluorescence of MDN1 (green), the nucleolar NPM1 protein (purple), and EZH2-stained foci (yellow). DNA was stained with DAPI (blue). **g**, Quantification of overlap between MDN1 foci and EZH2 or NPM1 per nucleus in the wild type cells. *p* values are from two-sided student’s t-tests. Data are presented as mean values +/− SEM.

Polycomb proteins localize to distinct foci in the nucleus, referred to as Polycomb bodies^39,40^. We next performed immunofluorescence staining using an antibody that recognizes the EZH2 subunit of PRC2 to test for colocalization of the rixosome and Polycomb bodies. We first validated each of the commercially available antibodies used in these experiments by showing that they recognized protein species that were depleted by specific siRNA treatments (Extended Data Fig. 3a-c). Consistent with the ChIP–seq results, immunofluorescence showed that MDN1 and WDR18 localized to closely overlapping domains with EZH2 Polycomb bodies (Extended Data Fig. 3d, e). The mammalian rixosome has previously been shown to localize to nucleoli, where it performs its rRNA processing functions^41^. To examine the relationship between Polycomb bodies and nucleoli, we stained cells for EZH2 and the nucleolar protein NPM1 and found that whereas the most intensely staining EZH2 foci co-localized with NPM1-stained nucleoli, the remaining EZH2 foci did not co-localize with NPM1 (Extended Data Fig. 3f, g).

## RING1A and B in rixosome recruitment

**Extended Data Fig. 4 ∣:**
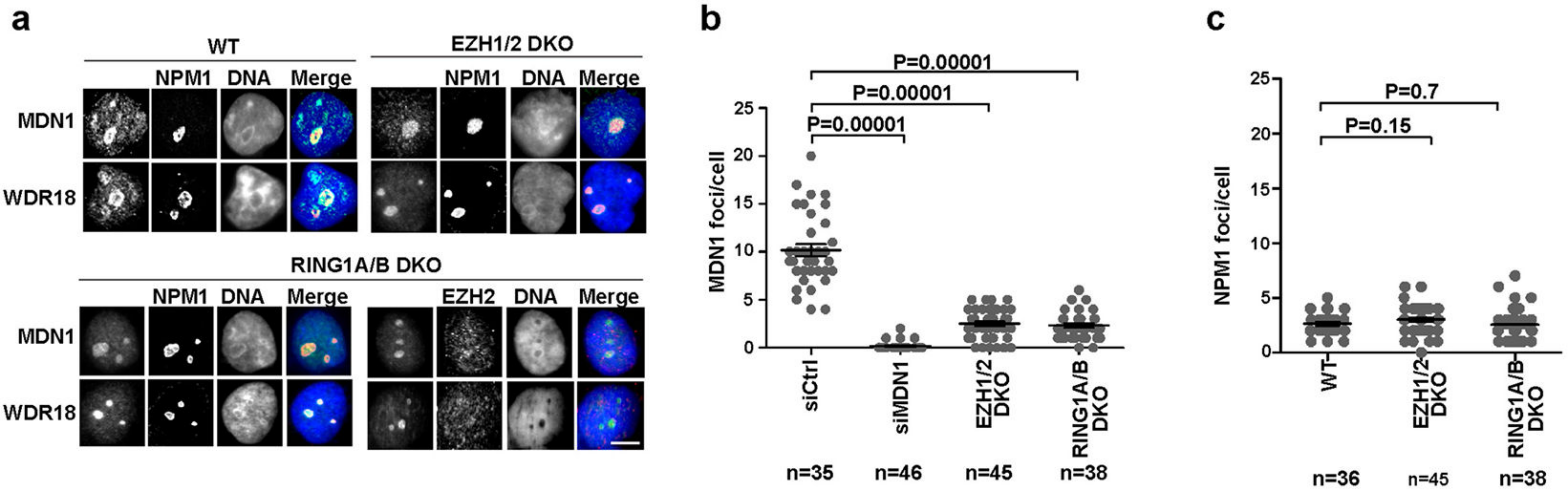
Polycomb body-enrichment of rixosome is EZH1/2- and RING1A/B-dependent. **a**, Immunofluorescence experiments showing colocalization of rixosome subunits MDN1 and WDR18 with the nucleolar marker NPM1 in wild-type (WT) and EZH1/2 double knockout cells, and with NPM1 and EZH2 in RING1A/B double knockout cells. Scale bar, 5 μm. **b, c**, Quantification of change in MDN1 (**b**) or NPM1 (**c**) foci per nucleus in the indicated cells. *p* values are from two-sided student’s t-tests. Data are presented as mean values +/− SEM.

**Fig. 3 ∣:**
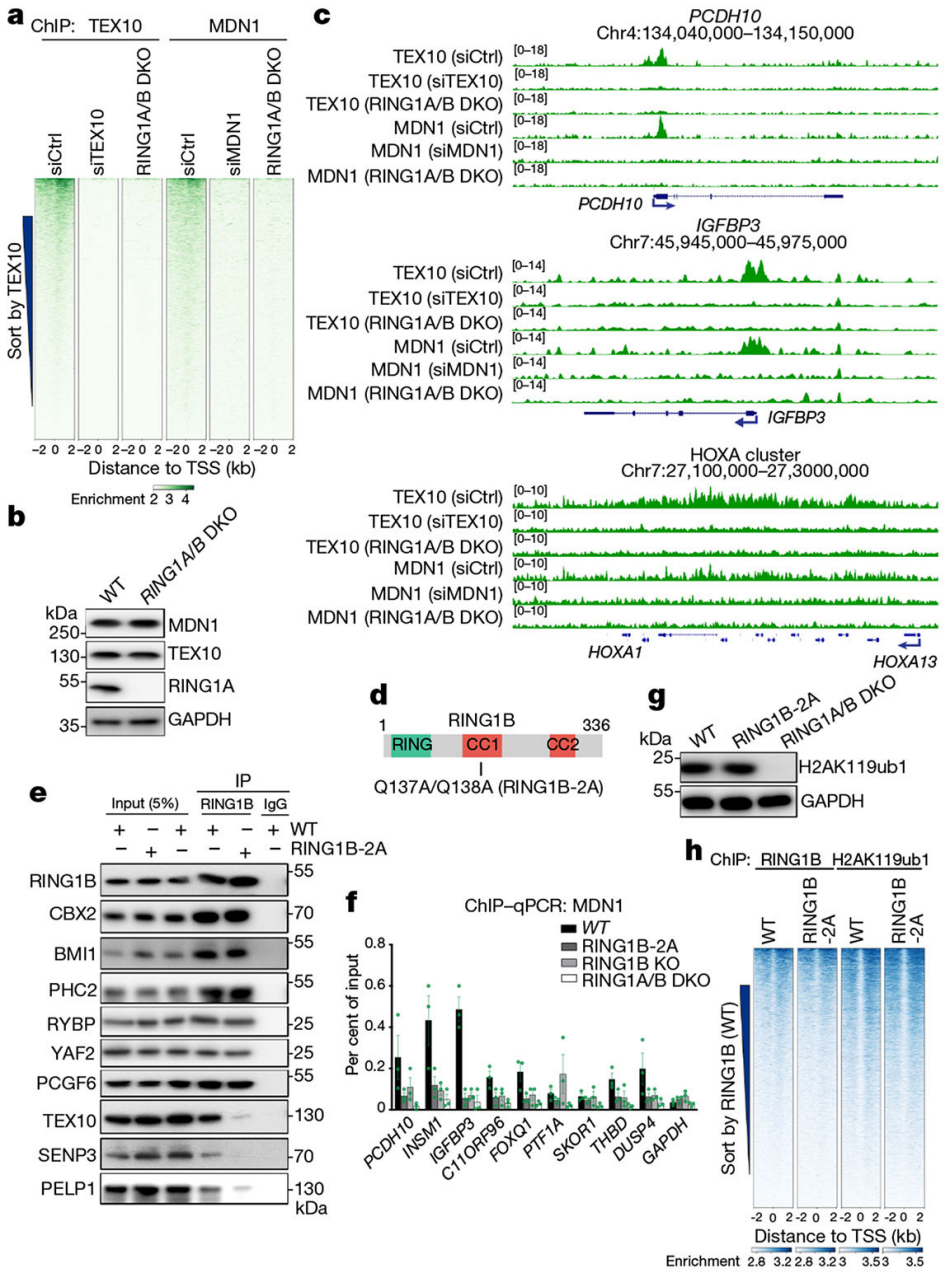
PRC1 is required for rixosome chromatin targeting. **a**, Heatmap representations of ChIP–seq of TEX10 and MDN1 in HEK 293FT cells with the indicated treatments. Rank order is from highest to lowest TEX10 signal in control siRNA (siCtrl)-treated cells. RING1A/B-DKO cells were treated with control siRNA. log_2_ enrichment levels were normalized to reads per genome coverage. Read counts per gene were summed in 50-nt bins. **b**, Immunoblots showing indicated protein levels in wild-type (WT) and RING1A/B-DKO cells. **c**, Genomic snapshots of ChIP–seq reads at Polycomb target genes *PCDH10, IGFBP3* and HOXA cluster for the indicated cells. log_2_ enrichment levels were normalized to reads per genome coverage. **d**, Schematic of RING1B protein and its domains. CC, coiled-coil domain. The location of RING1B-2A substitutions is indicated. **e**, Immunoprecipitations showing the effect of RING1B-2A substitutions on the interaction of RING1B with PRC1 subunits CBX2, PHC2, BMI1 (PCGF4), RYBP, YAF2 and PCGF6, and rixosome subunits PELP1, TEX10 and SENP3 in HEK 293FT cells. **f**, ChIP–qPCR experiments showing the localization of MDN1 at the indicated genes in wild type, RING1B-2A, RING1B-KO and RING1A/B-DKO cell lines. Primers used for quantitative PCR targeted the first exon of each gene. *GAPDH* served as a control. Data are presented as mean ± s.e.m. for three biological replicates. **g**, Immunoblots showing total H2AK119ub1 levels in wild-type, RING1B-2A and RING1A/B-DKO HEK 293FT cells. **h**, Heatmap representations of ChIP–seq of RING1B and H2AK119ub1 in wild-type, RING1B-2A HEK 293FT cells. Rank order is from highest to lowest RING1B signal in wild-type cells. log_2_ enrichment levels were normalized to reads per genome coverage. Read counts per gene were summed in 50-nt bins.

We next tested whether the localization of the rixosome to Polycomb target genes was Polycomb-dependent. As shown by heatmap analysis in Fig. 3a, the localization of both TEX10 and MDN1 to target loci was abolished in RING1A and RING1B (RING1A/B)-double-knockout (DKO) cells, whereas the levels of TEX10 and MDN1 were unaffected (Fig. 3b). Similarly, at the single-gene level, ChIP–seq signals for TEX10 and MDN1 on *PCDH10, IGFBP3* and HOXA genes were absent in the RING1A/B-DKO cells (Fig. 3c). Consistently, at the cytological level, the numbers of MDN1 and WDR18 foci were significantly reduced in RING1A/B-DKO or EZH1 and EZH2 (EZH1/2)-DKO cells, whereas the number of nucleoli (stained with NPM1) were unaffected (Extended Data Fig. 4a-c). Therefore, the localization of rixosome subunits to both Polycomb target genes and Polycomb bodies required the catalytic PRC subunits.

**Extended Data Fig. 5 ∣:**
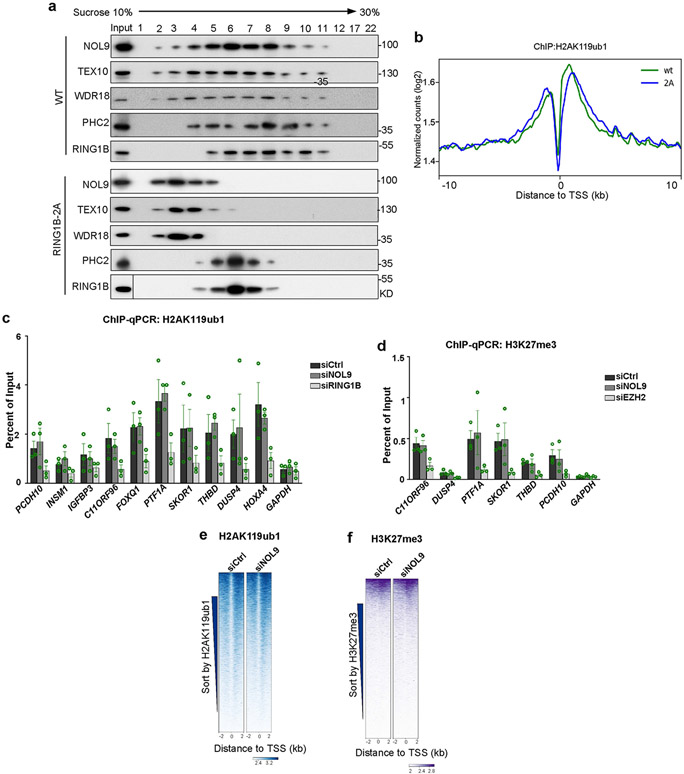
Rixosome effects on H3K27me3 and H2AK119ub1, and rixosome-Polycomb association. **a**, Co-fractionation of rixosome and PRC1 subunits. Flag-NOL9-associated and PHC2–Flag-associated proteins purified from cells with wild-type or *RING1B-2A* and were subjected to 10–30% sucrose gradient sedimentation. Fractions were collected and adsorbed to Strataclean beads and analyzed by immunoblotting with the indicated antibodies. **b**, Average distribution of H2AK119ub1 ChIP-seq reads (log2) for all annotated genes in wild type (WT) and *RING1B-2A (2A)* mutant HEK293FT cells. Enrichment levels were normalized with Reads Per Genome Coverage. Read counts per gene were summed in 50-nt bins. **c**, ChIP-qPCR experiments showing enrichment of H2AK119ub1 at the indicated target genes in siCtrl, siNOL9, and siRING1B treated HEK293FT cells. Error bars represent standard deviations for three biological replicates. Data are presented as mean values +/− SEM. **d**, ChIP-qPCR showing enrichment of H3K27me3 at the indicated target genes in siCtrl, siNOL9, and siEZH2 treated HEK293FT cells. Error bars represent standard deviations for three biological replicates. Data are presented as mean values +/− SEM. **e**, Heatmap representations of H2AK119ub1 ChIP-seq from control cells compared to cells depleted of NOL9 at TSS flanking regions. Rank order is by H2AK119ub1 signal from siCtrl cells. Enrichment levels (log2) were normalized with Reads Per Genome Coverage. Color bar at bottom indicates range of read counts per 50-nt bin. **f**, Heatmap representations of H3K27me3 ChIP-seq from control cells compared to cells depleted of NOL9 at TSS flanking regions. Rank order is by H3K27me3 signal from siCtrl cells. Enrichment levels (log2) were normalized with Reads Per Genome Coverage. Color bar at bottom indicates range of read counts per 50-nt bin.

To test whether the interaction of RING1B with the TEX10 subunit of the rixosome—observed with purified proteins (Extended Data Fig. 1g-i)—was required for the association of the rixosome with PRC1 and Polycomb target genes in cells, we used CRISPR–Cas9 to replace the chromosomal copies of RING1B with RING1B(Q137A/Q138A) (RING1B-2A) (Fig. 3d), which is impaired in its ability to bind to TEX10. As shown in Fig. 3e, the CBX2, BMI1 (also known as PCGF4) and PHC2 subunits of cPRC1, and the RYBP, YAF2 and PCGF6 subunits of vPRC1, co-immunoprecipitated with both wild-type and RING1B-2A proteins, indicating that the RING1B mutations did not disrupt the integrity of PRC1 complexes. However, whereas the TEX10, SENP3 and PELP1 subunits of the rixosome co-immunoprecipitated with wild-type RING1B, their interaction with RING1B-2A was greatly diminished (Fig. 3e). Consistent with the immunoprecipitation results, rixosome subunits NOL9, TEX10 and WDR18 co-migrated with PRC1 subunits PHC2 and RING1B during sucrose gradient sedimentation; this co-migration did not occur in extracts prepared from RING1B-2A mutant cells (Extended Data Fig. 5a). Moreover, experiments using chromatin immunoprecipitation followed by quantitative PCR (ChIP–qPCR) showed that, relative to wild-type RING1B, the interaction of MDN1 with several Polycomb target loci was diminished to a similar extent in RING1B-2A and RING1B-knockout (KO) cells, whereas as expected, RING1A/B-DKO cells displayed a greater loss of MDN1 binding (Fig. 3f). Together, these results indicate that recruitment of the rixosome to target loci requires its specific interaction with RING1B. Consistent with maintenance of PRC1 integrity, RING1B-2A mutant cells had similar total levels of H2AK119ub1 to the wild type and ChIP–seq experiments showed that the genome-wide localization of RING1B itself and H2AK119 ubiquitination were not affected by RING1B-2A (Fig. 3g, h). Similarly, the depletion of NOL9 did not affect histone H2AK119ub1 or H3K27me3 levels, which were greatly diminished upon the depletion of RING1B and EZH2, respectively (Extended Data Fig. 5b-f). The rixosome therefore acts downstream of Polycomb-catalysed histone modifications.

## Rixosome regulates nascent RNA synthesis

**Extended Data Fig. 6 ∣:**
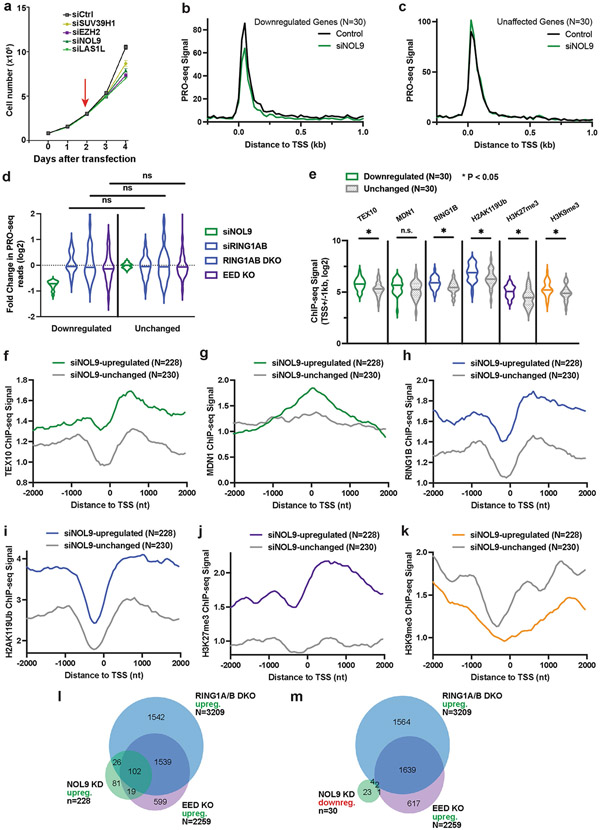
Rixosome subunits, H2AK119ub1, RING1B, and H3K27me3 are preferentially enriched at PRO-seq siNOL9-upregulated genes. **a**, Growth curves show cell number changes at indicated time points after knockdowns with siCtrl, siSUV39H1, siEZH2, siNOL9, or siLAS1L in HEK293FT cells. Error bars represent standard deviation for three biological replicates. Data are presented as mean values +/− SEM. **b**, Average distribution of PRO-seq signal is shown at genes downregulated by siNOL9 (N = 30). Data are shown in 25-nt bins. **c**, Average distribution of PRO-seq signal is shown at a set of genes unaffected by siNOL9 (N = 30) which were expression matched for the downregulated genes in **b**. Data are shown in 25-nt bins. **d**, Violin plots depict the log2 (fold change) in PRO-seq for siNOL9 downregulated (N = 30) and unaffected (N = 30) genes in siNOL9, siRING1B, *RING1AB* DKO, and *EED* KO cells. Knockout cells were treated with control siRNA. *p*-values are from two-tailed Mann-Whitney test. *P*=0.3581 for siRING1AB, *P* = 0.6438 for *RING1AB* DKO, *P* = 0.6228 for *EED* KO. **e**, Violin plots showing read counts for the indicated ChIP-seq experiments. Reads in were summed ± 1 kb from TSSs for the gene groups indicated. Violin plots depict the range of values, with median indicated by a line. *p*-values are from two-tailed Mann-Whitney test. *P* = 0.0034 for TEX10, *P* = 0.0648 for MDN1, *P* = 0.0058 for RING1B, *P* = 0.017 for H2AK119ub1, *P* = 0.0028 for H3K27me3, *P* = 0.0276 for H3K9me3. n.s., not significant. **f–k**, Average distribution of the indicated ChIP-seq reads at siNOL9-upregulated or siNOL9-unaffected genes. Read counts per gene were summed in 50-nt bins. **l–m**, Venn diagrams showing the overlap between siNOL9-upregulated (**l**) and siNOL9-downregulated (**m**) genes with genes upregulated in *RING1AB* DKO or *EED* KO cells in PRO-seq experiments.

We next investigated whether the rixosome was required for silencing of Polycomb target genes. We were unable to generate viable knockouts of several rixosome subunits, presumably owing to their essential roles in rRNA processing. We therefore used transient siRNA knockdown of rixosome subunits at timepoints that do not affect growth and proliferation to study the role of the rixosome in regulation of transcription. Growth curves after knockdown of the rixosome subunits NOL9 and LAS1L showed that 48 h of siRNA treatment did not affect cell proliferation (Extended Data Fig. 6a). We analysed changes in PolII levels and position at target genes by performing precision run-on sequencing (PRO-seq) 48 h after siRNA treatment. PRO-seq provides snapshots of transcriptionally engaged PolII with base-pair resolution^42^. In this way, we could focus on the direct transcriptional targets of the rixosome and Polycomb complexes, without the confounding effects of RNA processing or stability.

**Fig. 4 ∣:**
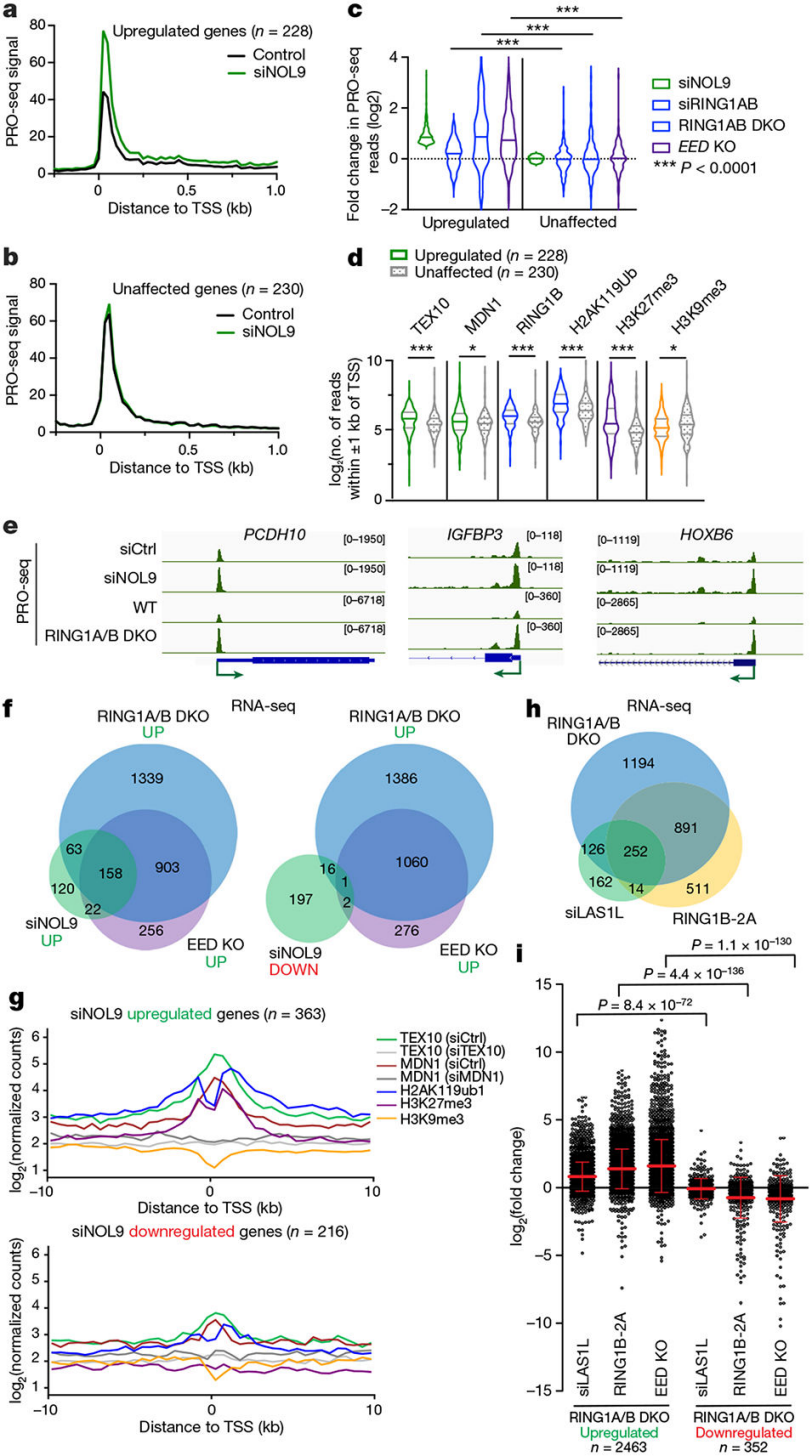
Loss of rixosome upregulates Polycomb target genes at the level of nascent RNA synthesis. **a**, Average distribution of PRO-seq signal is shown at genes upregulated by siNOL9 (*n* = 228). Data are shown in 25-nt bins. **b**, Average distribution of PRO-seq signal is shown at expression-matched genes unaffected by siNOL9 (*n* = 230). Data are shown in 25-nt bins. **c**, Violin plots depict the log_2_ fold change in PRO-seq for siNOL9 upregulated (*n* = 228) and unaffected (*n* = 30) genes in cells treated with siNOL9, siRING1B and RING1AB-DKO and EED-KO cells. Knockout cells were treated with control siRNA. *P* values are from two-tailed Mann–Whitney tests. *P* = 1.45 × 10^−5^ for siRING1AB, *P* = 1.98 × 10^−17^ for *RING1AB-DKO* and *P* = 3.39 × 10^−24^ for EED-KO. **d**, Violin plots showing read counts for the indicated ChIP–seq experiments. Reads were summed ±1 kb from the TSS for the gene groups indicated. Violin plots depict the range of values, with the centre line indicating the median. *P* values are from two-tailed Mann–Whitney tests. *P* = 1.55 × 10^−7^ for TEX10, *P* = 0.0178 for MDN1, *P* = 3.74 × 10^−10^ for RING1B, *P* = 2.34 × 10^−8^ for H2AK119ub1, *P* = 1.76 × 10^−11^ for H3K27me3 and *P* = 0.0321 for H3K9me3. **e**, Genome snapshots of PRO-seq experiments showing transcribing PolII at the indicated genes in siCtrl, siNOL9 and RING1A/B-DKO HEK 293FT cells. **f**, Venn diagrams showing overlap among upregulated (left) and downregulated (right) genes in cells treated with siNOL9 with upregulated genes in EED-KO and RING1A/B-DKO cells in RNA-seq experiments. Hypergeometric probability *P* values: siNOL9 upregulated versus RING1A/B-DKO, 3.1 × 10^−122^; siNOL9 upregulated versus EED-KO, 3.6 × 10^−125^; siNOL9 downregulated versus RING1A/B-DKO, 0.1; siNOL9 downregulated versus EED-KO, 1.2 × 10^−3^; RING1A/B-DKO versus EED-KO, 4.2 × 10^−854^. **g**, Average distribution of normalized log_2_ counts of the indicated ChIP–seq reads for genes that are upregulated (top) or downregulated (bottom) in HEK 293FT cells treated with siNOL9. Enrichment levels were normalized with reads per genome coverage. Read counts per gene were summed in 50-nt bins. **h**, Venn diagram showing overlap among upregulated genes in RING1B-2A, siLAS1L and RING1A/B-DKO cells in RNA-seq experiments; 1,143 genes (69%) were upregulated in both RING1B-2A-expressing and RING1A/B-DKO cells; 437 genes (79%) were overexpressed in both siLAS1L-treated and RING1A/B-DKO cells. Hypergeometric probability *P* values: RING1B-2A versus RING1A/B-DKO, 1.2 × 10^−805^; siLAS1L upregulated versus RING1A/B-DKO, 2.2 × 10^−239^; siLAS1L upregulated versus RING1B-2A, 8.2 × 10^−157^. **i**, Dot plots showing changes in gene expression detected by RNA-seq of RING1B-2A cells, siLAS1L-treated cells and EED-KO cells in the sets of genes that are upregulated or downregulated in RING1A/B-DKO HEK 293FT cells. Data are mean ± s.e.m. *P* value is from the two-tailed Mann–Whitney test.

PRO-seq analysis revealed a significant increase in the PRO-seq signal of 228 genes and decreases in the PRO-seq signal of 30 genes in siNOL9 cells (adjusted *P* value (*P*_adj_) < 0.05; fold change > 1.5), and metagene analyses showed change in RNA polymerase signal at both TSSs and gene bodies (Fig. 4a, b, Extended Data Fig. 6b). To assess how this set of NOL9 target genes was affected by loss of RING1A/B or EED, we compared them to sets of expression-matched genes that were not affected by NOL9 depletion (Fig. 4b, Extended Data Fig. 6c). In contrast to siNOL9-unaffected or downregulated genes, siNOL9-upregulated genes were also mostly upregulated in siRING1A/B, RING1A/B-DKO and EED-KO cells (Fig. 4c, Extended Data Fig. 6d). Furthermore, relative to siNOL9-unaffected or -downregulated genes, siNOL9-upregulated genes showed highly significant enrichment in ChIP–seq signals for rixosome subunits (TEX10 and MDN1), a PRC1 subunit (RING1B), H2AK119ub1 and H3K27me3, but not H3K9me3 (Fig. 4d, Extended Data Fig. 6e-k). Consistently, relative to siNOL9-downregulated genes, we observed a greater overlap between siNOL9-upregulated genes and those also upregulated in RING1A/B-DKO and EED-KO cells (Extended Data Fig. 6l, m). As examples at the single-gene level, we observed increased PRO-seq signal at the *PCDH10, IGFBP3* and *HOXB6* genes in siNOL9 and RING1A/B-DKO cells (Fig. 4e). The increase in PolII occupancy in siNOL9 cells was in general weaker than in RING1A/B-DKO cells, which may be owing to partial depletion of NOL9 by siRNA treatment or additional rixosome-independent functions of RING1A/B.

**Extended Data Fig. 7 ∣:**
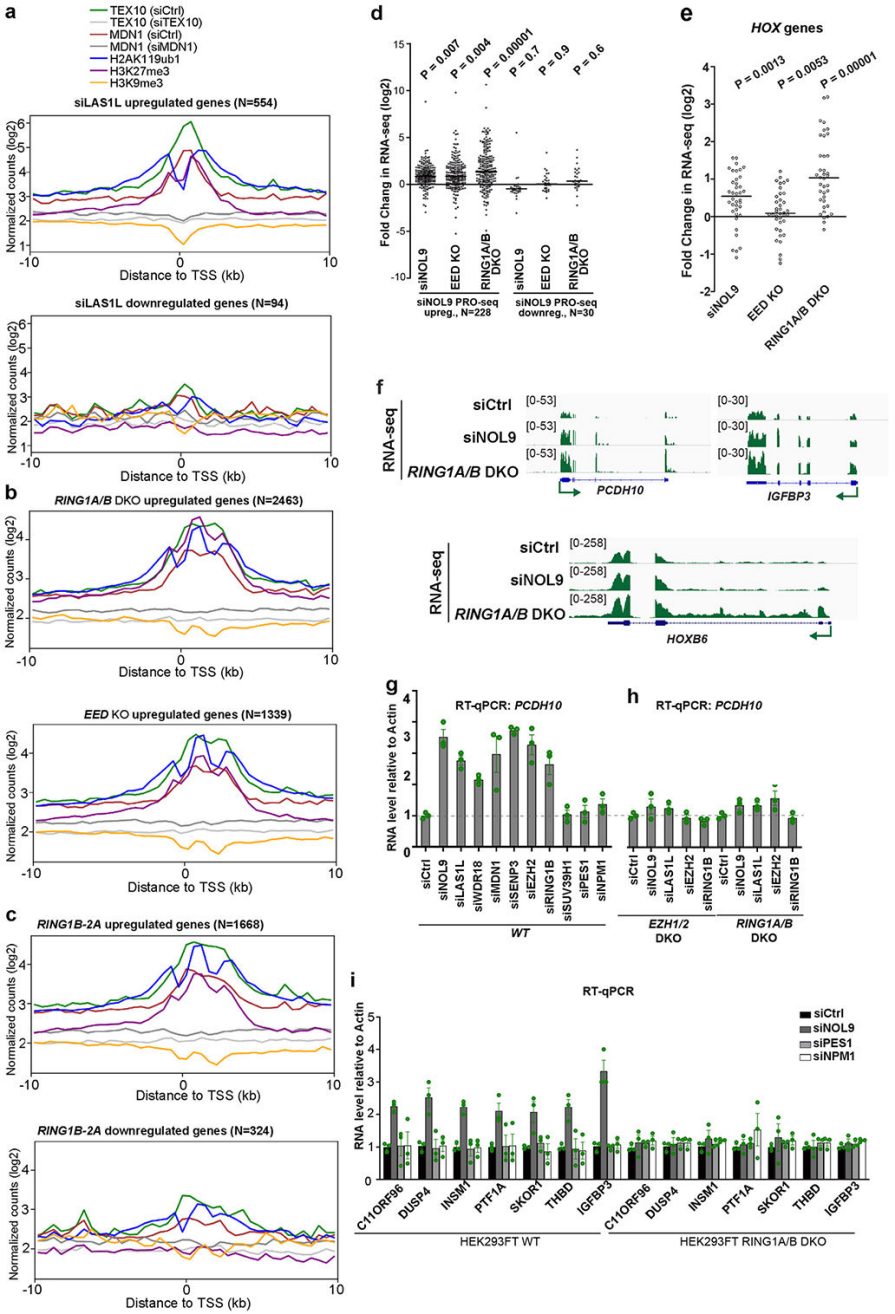
Coregulation of target genes by the rixosome and PRCs. **a–c**, Average distribution of the indicated ChIP-seq reads (log2) for genes upregulated and downregulated in *LAS1L KD* (**a**) and RING1B-2A(**b**), and upregulated genes in *EED* KO and *RING1A/B* DKO (**c**) RNA-seq experiments from HEK293FT cells. Enrichment levels were normalized with Reads Per Genome Coverage. Read counts per gene were summed in 50-nt bins. **d**, Dot plots showing RNA-seq changes in the expression of siNOL9-upregulated or siNOL9-downregulated genes in PRO-seq experiments in HEK293FT cells. siNOL9 PRO-seq upregulated genes have increased RNA levels in siNOL9, *EED* KO, and *RING1A/B* DKO cells. *p* value is from the two-tailed Wilcoxon test. The measure of center is median. **e**, RNA-seq experiments showing increased *HOX gene* expression in siNOL9 and *RING1A/B* DKO but not *EED* KO cells. *P* value is from the two-tailed Wilcoxon test. The measure of center is median. **f**, Genomic snapshots of RNA-seq reads showing the effect of siRNA knockdown of NOL9 and *RING1A/B* DKO on the expression of the indicated genes in HEK293FT cells. **g, h**, RT-qPCR assays showing that siRNA knockdown of rixosome subunits results in increased expression of *PCDH10* in wild-type (WT), but not *EZH1/2* DKO or *RING1A/B* DKO HEK293FT cells. Actin *(ACTB)* served as a normalization control. Every knockdown was normalized to siCtrl. Nucleolar PES1 and NPM1 served as controls for possible non-specific effects resulting nucleolar perturbations. Error bars represent standard deviations for three biological replicates. Data are presented as mean values +/− SEM. **i**, RT-qPCR experiments showing the effect of the indicated siRNA knockdowns on the indicated Polycomb and rixosome target genes in wild-type (WT) cells and *RING1A/B* DKO cells. Actin *(ACTB)* served as a normalization control. Every knockdown was normalized to siCtrl. Error bars represent standard deviations for three biological replicates. Data are presented as mean values +/− SEM.

In agreement with the PRO-seq results, RNA-sequencing (RNA-seq) experiments showed that in contrast to siNOL9-downregulated genes, siNOL9-upregulated genes largely overlapped with genes upregulated in RING1A/B-DKO and EED-KO cells (Fig. 4f). Metagene analysis of genes affected in RNA-seq indicated that the siNOL9- and siLAS1L-upregulated, but not the downregulated genes were enriched for rixosome subunits and Polycomb-catalysed histone modifications (Fig. 4g, Extended Data Fig. 7a, b). Moreover, similar to RING1A/B-DKO, Polycomb target genes were upregulated in cells expressing RING1B-2A (Fig. 4h). Additionally, as was the case with RING1A/B-DKO-upregulated genes, RING1B-2A-upregulated genes were enriched for rixosome subunits and Polycomb-catalysed histone modifications (Extended Data Fig. 7c). Together with the observations that RING1B-2A mutation or rixosome-subunit depletions did not affect H2AK119ub1 or H3K27me3 levels (Fig. 3g, h, Extended Data Fig. 5a-e), these results suggest that the rixosome and Polycomb complexes regulate a common set of genes at the level of transcription and that the rixosome acts downstream of Polycomb-catalysed histone modifications.

In agreement with the above analysis, RNA-seq experiments showed that the genes with increased PRO-seq signal in siNOL9, RING1A/B-DKO and EED-KO cells also had increased steady state RNA expression (Extended Data Fig. 7d). The set of siNOL9-upregulated genes in HEK 293FT cells included most of the HOX genes, which were also upregulated in RING1A/B-DKO but not in EED-KO cells (Extended Data Fig. 7e). This observation is consistent with the presence of H2AK119ub1 but little or no H3K27me3 at HOX genes in these cells (Fig. 2f). For example, genomic browser snapshots of RNA-seq reads showed that the depletion of either NOL9 or RING1A/B resulted in increased expression of the *PCHD10, IGFBP3* and *HOXB6* genes (Extended Data Fig. 7f). Notably, in contrast to wild-type cells, depletion of the rixosome subunits in EZH1/2-DKO or RING1A/B-DKO cells had no effect on the expression the *PCHD10* and several other target genes, indicating that the rixosome and Polycomb act epistatically through the same pathway (Extended Data Fig. 7g-i). As controls for possible indirect effects due to perturbation of ribosome biogenesis in the above experiments, siRNA knockdown of nucleolar NPM1 and PES1 proteins had no effect on the expression of several rixosome target genes (Extended Data Fig. 7g, i). Together, these results demonstrate that the rixosome and Polycomb complexes repress a largely shared set of genes in HEK 293FT cells.

## Rixosome functions in other cell types

**Extended Data Fig. 8 ∣:**
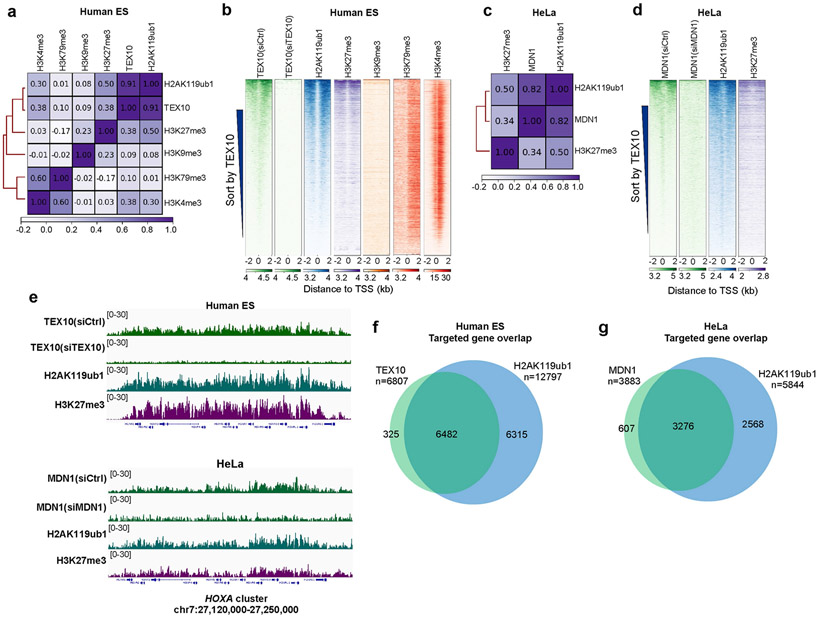
Colocalization of the rixosome with H2AK119ub1 in human ES and HeLa cells. **a**, Matrix depicting Spearman correlation coefficients between ChIP-seq datasets calculated using read counts summed +/−2 kb for all annotated gene TSSs (hg19) in human embryonic stem (ES) cells. **b**, Heatmap representations of ChIP-seq of TEX10 and histone modifications (H2AK119ub1, H3K27me3, H3K9me3, H3K79me3, and H3K4me3,) in human ES cells. Rank order is from most to least TEX10 signal. Enrichment levels (log2) were normalized with Reads Per Genome Coverage. Read counts per gene were summed in 50-nt bins. **c**, Matrix depicting Spearman correlation coefficients between ChIP-seq datasets calculated using read counts summed +/−2 kb for all annotated gene TSSs (hg19) in HeLa cells. **d**, Heatmap representations of ChIP-seq of MDN1, H2AK119ub1, and H3K27me3 in HeLa cells. Rank order is from most to least TEX10 signal. Enrichment levels (log2) were normalized with Reads Per Genome Coverage. Read counts per gene were summed in 50-nt bins. **e**, Genomic snapshots of ChIP-seq reads at Polycomb target *HOXA* cluster in human ES (top) and HeLa (bottom) cells for the indicated rixosome subunits or Polycomb histone modifications. Enrichment levels (log2) were normalized with Reads Per Genome Coverage. **f**, Venn diagram showing overlap between TEX10- and H2AK119ub1-enriched TSSs in human ES cells. Hypergeometric probability *p* values, 2.9e-4262 **g**, Venn diagram showing overlap between MDN1- and H2AK119ub1-enriched TSSs in HeLa cells. Hypergeometric probability *p* values, 8.9e-2935.

**Extended Data Fig. 9 ∣:**
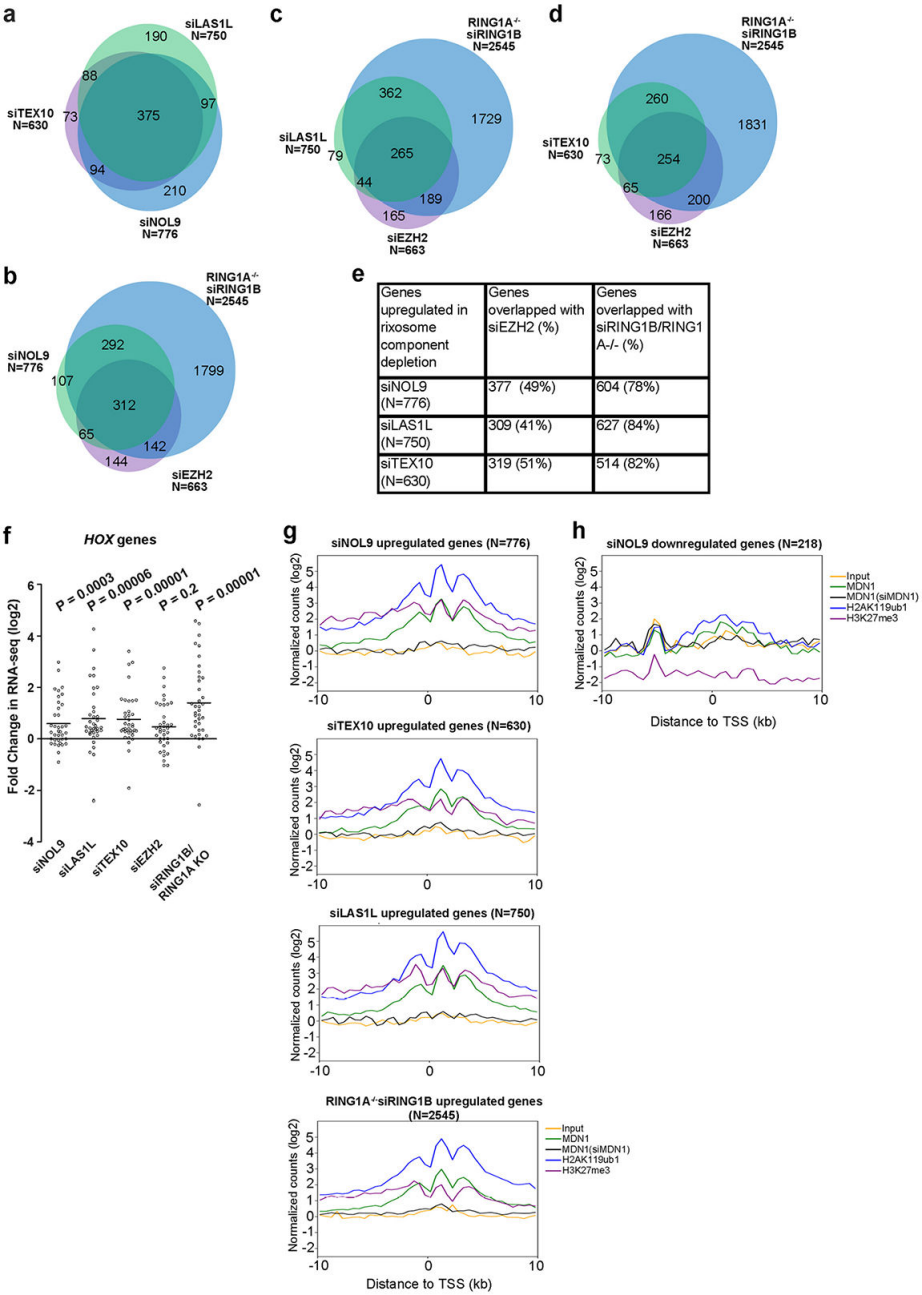
Role of the rixosome in Polycomb silencing is not cell type specific. **a**, Venn diagram showing overlap among genes upregulated in RNA-seq analysis of siNOL9, siLAS1L, and siTEX10 HeLa cells. Hypergeometric probability *p* values: siNOL9 vs siLAS1L, 2.6e-765; siNOL9 vs siTEX10, 1.4e-859; siLAS1L vs TsiEX10, 1.6e-853. **b**, Same as in **a** but showing overlap among genes upregulated in siRING1B in *RING*1A KO (siRING1B, *RING1A−/−),* siNOL9 (in wild type), and siEZH2 (in wild type). Hypergeometric probability *p* values: siNOL9 vs siRING1B, *RING1A−/−,* 4.1e-441; siNOL9 vs siEZH2, 4.6e-411; siEZH2 vs siRING1B, *RING1A−/−,* 2.9e-284. **c**, Same as in **a** but showing overlap among genes upregulated in siRING1B, *RING1A−/−,* siLAS1L (in wild type), and siEZH2 (in wild type). Hypergeometric probability *p* values: siLAS1L vs siRING1B, *RING1A−/−,* 1.4-496; siLAS1L vs siEZH2, 1.7e-298. **d**, Same as in **a** but showing overlap among genes upregulated in siRING1B, *RING1A−/−,* siTEX10 (in wild type), and siEZH2 (in wild type). Hypergeometric probability *p* values: siTEX10 vs siRING1B, *RING1A−/−,* 1.8e-391; siTEX10 vs siEZH2, 9.1e-347. **e**, Table showing the percentages of overlapping upregulated genes between rixosome and PRC depletions in panels **a–d**. **f**, Dot plots of RNA-seq experiments showing changes in the expression of 39 *HOX* genes in HeLa cells. *p* values are from two-tailed Wilcoxon test. The measure of center is median. **g**, Average distribution of indicated ChIP-seq reads (log2) for genes upregulated by siRNA depletion of TEX10, LAS1L, NOL9, and RING1B (RING1A^−/−^) in HeLa cell RNA-seq experiments. Enrichment levels were normalized with Reads Per Genome Coverage. Read counts per gene were summed in 50-nt bins. **h**, Same as in **g** but showing siNOL9 downregulated genes.

To investigate the rixosome–Polycomb connection in other cell types, we examined the genome-wide localization of TEX10 in human embryonic stem (ES) cells and MDN1 in HeLa cells. Consistent with the results in HEK 293 cells, correlation and heatmap analysis of ChIP–seq reads indicated similar enrichment patterns for TEX10 (Extended Data Fig. 8a, b) and MDN1 (Extended Data Fig. 8c, d) with H2AK119ub1 and/or H3K27me3, but not with H3K9me, in human ES cells and HeLa cells. For example, TEX10 and MDN1, along with H2AK119ub1 and H3K27me3, co-localized to the entire HOXA cluster in human ES cells and HeLa cells (Extended Data Fig. 8e). Of note, 82% of TEX10-enriched TSSs in human ES cells and 76% of MDN1-enriched TSSs in HeLa cells overlapped with H2AK119ub1 peaks (Extended Data Fig. 8f, g). We also performed RNA-seq experiments in HeLa cells with siRNA knockdowns. As expected, we observed a high degree of correlation between the genes that were upregulated upon the knockdown of NOL9, LAS1L and TEX10 rixosome subunits (Extended Data Fig. 9a). Moreover, consistent with the results in HEK 293 cells, a large fraction of the genes upregulated in siNOL9, siLAS1L, and siTEX10 cells overlapped with those upregulated in RING1A-KO, siRING1B or siEZH2 cells (Extended Data Fig. 9b-e). As in HEK 293 cells, depletion of rixosome subunits in HeLa cells resulted in increased expression of HOX genes, which were also upregulated in siEZH2 cells, consistent with their association with H3K27me3 in these cells (Extended Data Fig. 8e, Extended Data Fig. 9f). Similarly, metagene analysis indicated that the upregulated, but not downregulated genes were enriched for rixosome subunits and Polycomb-catalysed histone modification (Extended Data Fig. 9g, h). The larger overlap between rixosome- and Polycomb-repressed genes in HeLa and human ES cells is probably owing to differences in siRNA knockdown efficiencies in these cells and/or in regulatory strategies. The rixosome therefore contributes to Polycomb silencing in different cell types.

## Rixosomal RNA degradation and silencing

**Extended Data Fig. 10 ∣:**
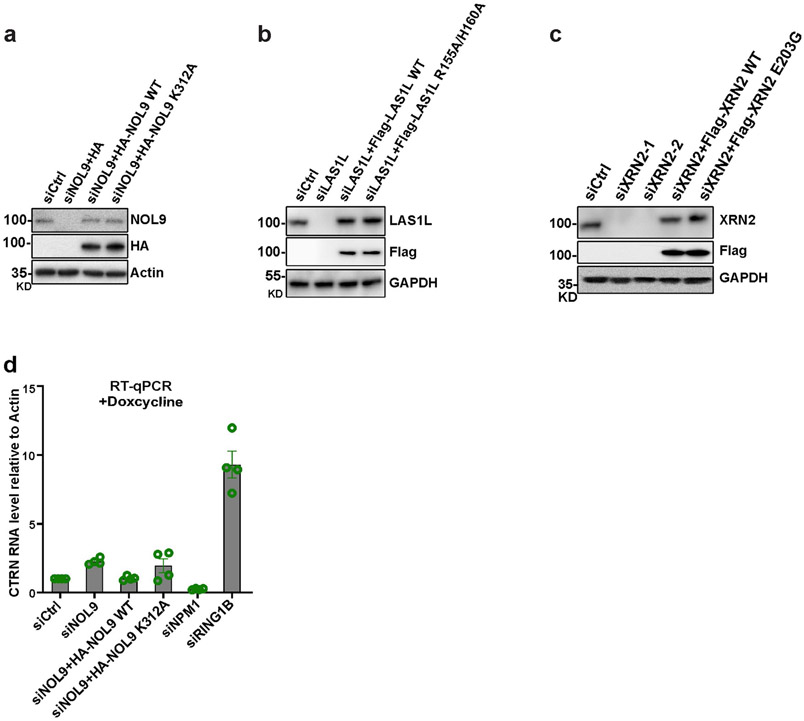
NOL9, LAS1L, and XRN2 catalytic point mutations. **a**, Immunoblot showing protein levels in control siControl (siCtrl), siNOL9, siNOL9+NOL9 wild type expressing plasmid, and siNOL9+ NOL9-K312A expressing plasmids. Actin served as a loading control. **b**, Immunoblot showing protein levels in control siControl (siCtrl), siLAS1L, siLAS1L+LAS1L wild type expressing plasmid, and siLAS1L+ LAS1L-R155A/H160A (LAS1l-2A) expressing plasmids. GAPDH served as a loading control. **c**, Immunoblot showing protein levels in control siControl (siCtrl), siXRN2-1 (siRNA 1), siXRN2-2 (siRNA 2), siXRN2-1+XRN2 wild type expressing plasmid, and siXRN2-1+ XRN2 E203G expressing plasmids. GAPDH served as a loading control. **d**, RT-qPCR analysis of RNA levels of H2B-CTRN in the indicated siRNA-treated and NOL9-rescued HEK293FT cells after 21 days of Doxcyline treatment. RNA expression levels were normalized to *ACTB*, and every knockdown was normalized to siCtrl. Error bars represent standard deviations for three biological replicates. Data are presented as mean values +/− SEM.

**Fig. 5 ∣:**
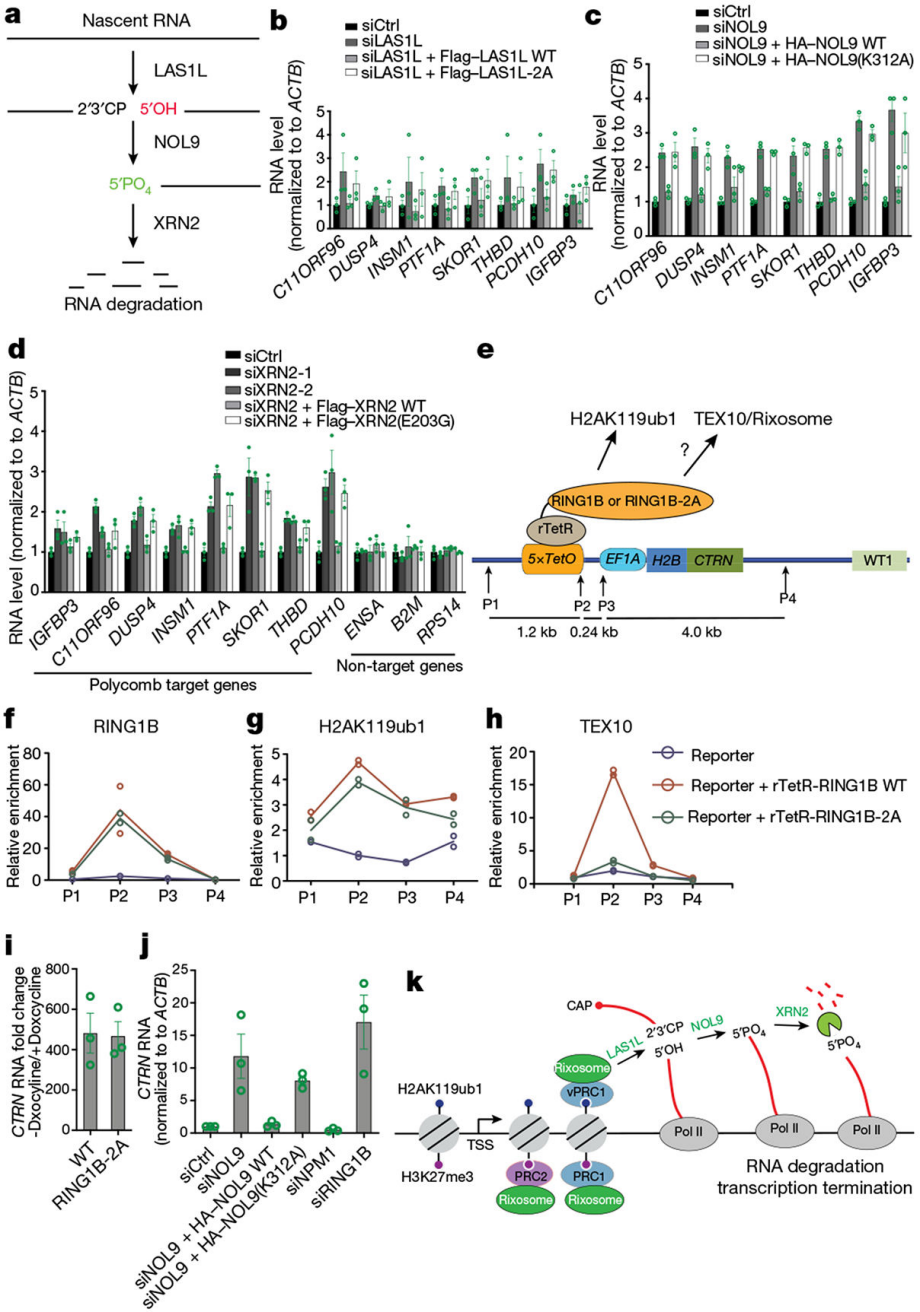
Rixosome-associated RNA degradation is required for repression of Polycomb-regulated genes. **a**, Schematic of rixosome RNA-processing activities depicting the hypothesis that the LAS1L endonuclease and RNA NOL9 kinase activities are required to prepare target RNA for XRN2-mediated degradation. **b–d**, Quantitative PCR with reverse transcription (RT–qPCR) analysis of expression of the indicated Polycomb target genes in the indicated siRNA-treated HEK 293FT cells rescued with LAS1L (**b**), NOL9 (**c**) or XRN2 (**d**). Expression levels were normalized to *ACTB* and siCtrl-treated cells. Data are presented as mean ± s.e.m. for three biological replicates. **e**, Schematic showing construction of cell lines with a 5×tetO-H2B-CITRINE (H2B-CTRN) reporter gene expressing rTetR–RING1B wild type or rTetR–RING1B-2A fusion proteins. **f–h**, ChIP–qPCR analysis of H2B-CTRN reporter enrichment for RING1B (**f**), H2AK119ub1 (**g**) and TEX10 (**h**) following 21 days of doxycycline treatment. ChIP–qPCR signals were normalized to *GAPDH*. Dots represent individual biological replicates. **i**, RT–qPCR analysis showing H2B-CTRN reporter RNA expression in rTetR–RING1B-expressing wild-type or RING1B-2A HEK 293FT cells before treatment relative to after 21 days of doxycycline treatment. RNA expression levels were normalized to *ACTB*. Data are presented as mean ± s.e.m. for three biological replicates. **j**, RT–qPCR analysis of H2B-CTRN RNA expression in the indicated siRNA-treated and NOL9-rescued HEK 293FT cells 3 days after release from 21-day doxcyline treatment. Expression levels were normalized to *ACTB* and to siCtrl-treated cells. Data are presented as mean ± s.e.m. for three biological replicates **k**, Model for the role of rixosome in Polycomb-mediated gene silencing. The rixosome is recruited through the interaction of RING1B in the vPRC1 and PRC1 complexes with the TEX10 subunit of the rixosome (individual subunits not shown) to mediate nascent RNA degradation and transcription termination. The rixosome also interacts with PRC2. See text for details.

The rixosome contains RNA endonuclease and polynucleotide kinase activities that prepare target RNAs for further degradation by the 5′–3′ XRN2 exoribonuclease^6,43^ (Fig. 5a). Cleavage of target RNA by the LAS1L endoribonuclease subunit of the rixosome generates a 5′-OH group, which must be phosphorylated by the NOL9 polynucleotide kinase subunit for the RNA to become a substrate for degradation by XRN2^6^ (Fig. 5a). We performed depletion and rescue experiments to first test whether the enzymatic activities of each LAS1L and NOL9 were required for their silencing functions. The upregulation of several target genes by the depletion of either LAS1L or NOL9 was rescued by the reintroduction of siRNA-resistant wild-type (WT) versions (Flag–LAS1L(WT) or haemagglutinin (HA)–NOL9(WT)) but not their catalytically dead mutant versions^44,45^ (Flag–LAS1L-2A or HA–NOL9(K312A)) (Fig. 5b, c). The requirements for the endonuclease activity of LAS1L and the polynucleotide kinase activity of NOL9, respectively, strongly suggest that the rixosome mediates target RNA degradation via the XRN2 exoribonuclease (Fig. 5a). To test this hypothesis, we knocked down XRN2 with two different siRNAs and found that several targets of the rixosome and Polycomb pathways were expressed at elevated levels in the knockdown cells, whereas three non-target loci were not affected (Fig. 5d; see Extended Data Fig. 10a-c for knockdown validation). Furthermore, the silencing defects resulting from XRN2 depletion were rescued by wild-type (Flag–XRN2(WT)) but not a catalytically dead^46^ XRN2 (Flag–XRN2(E203G)). We therefore conclude that the rixosome and XRN2 work together to degrade RNA at Polycomb target loci.

## Ectopic RING1B can recruit the rixosome

To provide further evidence that RING1B could recruit the rixosome to chromatin, we fused wild-type or RING1B-2A to the bacterial reverse tetracycline repressor (resulting in rTetR–RING1B or rTetR–RING1B-2A) and tested whether they could recruit TEX10 to TetR binding sites inserted together with a reporter gene at a euchromatic locus (*5xtetO-CTRN*) (Fig. 5e). ChIP–qPCR experiments showed that both wild-type RING1B and RING1B-2A mutant proteins were recruited to the ectopic locus and induced similar levels of H2AK119ub1 (Fig. 5f, g). However, whereas wild-type RING1B recruited high levels of TEX10 to the ectopic locus, RING1B-2A, which does not interact with TEX10 or the rixosome, recruited little or no TEX10 (Fig. 5h). The low levels of TEX10 recruitment induced by RING1B-2A are probably mediated by binding of the endogenous wild-type RING1B (PRC1) to H2AK119ub1 at the ectopic locus. Therefore, consistent with biochemical data and in vivo analysis of the requirements for rixosome localization to Polycomb target genes, these results demonstrate that RING1B can directly recruit the rixosome to chromatin.

We next tested the effect of RING1B tethering and depletion of NOL9 on the expression of the *5xtetO-CTRN* reporter. In the presence of doxycycline, which induces strong binding of rTetR fusion proteins to 5xtetO sites, we observed several-hundred-fold repression of *CTRN* reporter RNA for both rTetR–RING1B and rTetR–RING1B-2A tethering (Fig. 5i). However, depletion of NOL9 resulted in only weak derepression of the reporter (Extended Data Fig. 10d). We reasoned that the continuous strong binding of rTetR–RING1B to the reporter locus may partially mask the requirement for the rixosome. To test this hypothesis, we performed siRNA depletion experiments three days after the release of rTetR–RING1B (removing doxycycline from the medium). Under these conditions, depletion of NOL9 resulted in strong derepression of the *CTRN* reporter, which was rescued by wild-type but not catalytically dead NOL9 (Fig. 5j). As controls, depletion of RING1B, but not NPM1, resulted in strong derepression of the reporter gene in both the absence and presence of doxycycline (Fig. 5j, Extended Data Fig. 10d). Therefore, similar to endogenous loci, RING1B-mediated rixosome recruitment contributes to silencing at the ectopic locus.

## Discussion

Our findings demonstrate a role for the conserved rRNA processing and ribosome biogenesis complex, the rixosome, in Polycomb-mediated gene silencing. We demonstrate that the rixosome is recruited to chromatin in a PRC1-dependent manner by binding to RING1B and our PRO-seq analysis of active transcription shows that many genes targeted by these pathways contain paused PolII downstream of their promoter regions. Upon the loss of either the rixosome or Polycomb, the density of both the paused and elongating polymerase at these target genes increases, suggesting that Polycomb-mediated rixosome recruitment blocks productive transcription elongation by paused and/or elongating polymerase, thereby repressing gene activity.

In one model, silencing by Polycomb complexes is thought to involve chromatin compaction to block transcription initiation^1,2^. In both flies and mammals, subunits of the PRC1 complex can condense nucleosomal arrays in vitro and in vivo^7,22-24^ and, in mammals, PRC2 alone has in vitro chromatin compaction activity^25,26^. Moreover, recent studies show that the CBX2 subunit of the cPRC1 complex, which mediates its chromatin compaction activity, also promotes liquid–liquid phase separation in vitro and in vivo^27-29^. Our identification of a role for the rixosome in silencing of Polycomb target genes suggests that an additional layer of regulation involving RNA degradation has an important role in silencing of Polycomb target genes (Fig. 5k). We propose that the rixosome, once recruited to repressed genes by PRC1 and/or PRC2 complexes, surveys these loci for the presence of nascent RNA. At loci where Polycomb-mediated repression is weak and PolII enters early elongation, the rixosome recognizes and associates with nascent RNA to process it for degradation (Fig. 5k). Accordingly, we provide evidence that rixosome-cleaved RNAs become substrates for the 5′–3′ exoribonuclease XRN2, suggesting a role for nascent RNA cleavage and transcription termination in the potent silencing of Polycomb target genes. Heterochromatin-associated RNA degradation appears to have diverse and broadly conserved roles in gene silencing. In fission yeast, plants and animals, RNA interfence-dependent and -independent RNA degradation contributes to heterochromatin establishment and maintenance^5,47^. More recently, the LSM2–8 RNA decapping complex has been reported to act together with the XRN2 exonuclease to ensure full silencing of H3K27me3 loci in *Caenorhabditis elegans*^48^, suggesting that distinct mechanisms may act upstream of XRN2-mediated RNA degradation at Polycomb loci.

The rixosome may also regulate how chromatin-associated RNAs affect other Polycomb functions. PRC2 has been shown to interact with RNA promiscuously and with high affinity^49,50^, and RNA has been suggested to have both positive and negative roles in promoting the association of PRC2 with chromatin^51-53^. Rixosome-mediated RNA degradation may coordinate the different effects of RNA, particularly if the positive and negative roles of RNA were temporally regulated. The roles of the rixosome in the silencing functions of different types of chromatin, constitutive H3K9me heterochromatin in fission yeast^4^ and facultative H2AK119ub1 and H3K27me3 heterochromatin in human cells (this study), suggest that its RNA-degradation activities have highly conserved and critical functions in heterochromatin-mediated gene silencing.

## Methods

### Plasmid construction

Rixosome subunits (NOL9, WDR18, PELP1, TEX10), PRC2 subunits (EZH2, EED, SUZ12), PRC1 subunits (RING1B, PCGF1-4) and CBX1-8 cDNAs were amplified from human ES cell cDNA library and inserted to pGAD-T7 (Takara, 630442) and pGBK-T7 (Takara, 630443) plasmids for Y2H assays. NOL9 siRNA resistant cDNA was generated by PCR. The siRNA target sequence was mutated from 5′-AGACCTAAGTTCTGTCGAA-3′ to 5′-CGGCCGAAATTTTGCAGGA-3′ and integrated into the pCI (Promega, E1731) plasmid for ectopic protein expression. For bacteria protein expression, cDNA was integrated to pGEX-6P-1 (GE Healthcare, 28-9546-48).

### Y2H assays

Y2H budding yeast strain (Takara) was cultured with YEPD+adenine overnight at 30 °C. Yeast cells were collected OD 0.5 by centrifugation at 3,000 rpm for 3 min. Cells were resuspended and washed 2 times with 0.1 M LiAc (in 1x TE buffer). The bait pGBKT7 (0.5 μg) expressing rixosome, Polycomb, and HP1proteins and prey pGADT7 (0.5 μg) vectors were mixed with 10 μg carrier DNA, and further mixed with yeast cells collected from 10-ml cultures and resuspended in 50 μl 0.1 M LiAc (in 1× TE buffer). DNA-yeast mixture was incubated with 130 μl 40% PEG 4000 for 30 min at 30 °C. For transformation, 21 μl DMSO was added and mixed well with the yeast–DNA mixture, followed by heat shock at 42 °C for 20 min. After incubation on ice for 3 min, the cells were pelleted by centrifugation for 3 min at 4 °C. The supernatant was then discarded and sterile water was added to resuspend the cells, which were plated on double selective medium SC plates (Trp-, Leu-) for 3 days at 30°C. Colonies were further transferred to quadruple selective medium SC plates (Trp-, Leu-, His-, Ade-) for 3–4 days at 30 °C. For spotting assays, cells were incubated overnight in 4 ml double selective SC medium (Trp-, Leu-). The cells were then diluted to an optical density at 600 nm of 1, one millilitre of which was pelleted, washed once with sterilized water, resuspended in 250 μl sterilized water, and transferred to 96-well plates. Three microlitres of cell suspension from each well was plated on double-selective medium SC plates (Trp-, Leu-) and quadruple-selective medium SC plates (Trp-, Leu-, His-, Ade-) for four days.

### Cell culture

HeLa (ATCC, CCL-2), and HEK 293FT (ThermoFisher, R70007) cells were cultured in DMEM containing 10% fetal calf serum. Human embryonic stem cells were authenticated by the Initiative for Genome Editing and Neurodegeneration of Harvard Medical School and cultured as previously described^54^. In brief, cells cultures on 0.08 mg ml^−1^ matrigel coated plates with DMEM/F12 (containing 5 μg ml^−1^ insulin and 10 μg ml^−1^, 0.1 μg ml^−1^ FGF2, 1.7 ng ml^−1^ TGFβ1, 10 μg ml^−1^ transferrin). Cells were tested for mycoplasma contamination by the suppliers and were negative.

### RNAi

For siRNA-mediated knockdown, Lipofectamine RNAiMAX transfection reagent (Invitrogen) and siRNA (200 nM) were used to transfect the cells by following the manufacturer’s instructions. All the siRNAs were synthesized by Dharmacon and are listed in Supplementary Table 1.

### CRISPR–Cas9-mediated human genome editing

Small guide RNA was synthesized via in vitro transcription by using MAXIscript T7 transcription kit (ThermoFisher, AM1312). CRISPR–Cas9 protein was purified by the Initiative for Genome Editing and Neurodegeneration Core in the Department of Cell Biology at Harvard Medical School. DNA Oligonucleotide templates (synthesized by IDT, Supplementary Table 2), guide RNA, and CRISPR–Cas9 protein were delivered to cells by electroporation with Neon transfection system (ThermoFisher). Clones were screened by PCR and Miseq sequencing (Illumina).

### Immunofluorescence

Cells were placed on plates with cover slides. Cells were first washed with PBS, and fixed and permeabilized with methanol for 8 min at −20 °C. Cells were then incubated for 4–10 h at 4 °C with primary antibodies in PBS containing 4% bovine serum, which was followed by staining with secondary antibodies and 1 μg ml^−1^ DAPI. A confocal microscope (Nikon, Ti with perfect focus and spinning disk) equipped with a 60×/1.40 NA objective lens was used to image cells. NIS-Elements imaging software was used for imaging data collection. Images were post-processed with ImageJ (NIH) and photoshop (Adobe) software. EZH2 and MDN1 fluorescence intensities were assessed using ImageJ. NPM1 foci were counted visually directly using ImageJ. For MDN1 foci, the signal was measured in the regions with NPM1 in control cells, foci with the lowest value of NPM1 staining in the control cells was then used as a cutoff and any foci measured by ImageJ with higher value were counted as MDN1 foci. A list of antibodies and their sources is described in Supplementary Table 3.

### Immunoprecipitation and mass spectrometry analysis

To prepare chromatin-enriched fractions, cells were washed with PBS and then resuspended in ice-cold hypotonic buffer (10 mM HEPES, pH7.9, 1.5 mM MgCl_2_, 10 Mm KCl, 0.2 mM PMSF, 0.2 mM DTT) and incubated on ice for 10 min. Cell membranes were then disrupted by douncing 10 times. Nuclei were pelleted by centrifugation at 2,000g for 10 min, resuspended in cell lysis buffer (50 mM Hepes, pH 7.4, 150 mM NaCl, 1 mM MgCl_2_, 1 mM EGTA, and 0.5% Triton X-100) by pipetting for 3 min, and pelleted by centrifugation at 2,000g for 10 min to obtain a chromatin fraction. The chromatin pellet was resuspended in IP buffer (50 mM Hepes, pH 7.4, 250 mM NaCl, 10% glycerol, 1 mM MgCl_2_, 1 mM EGTA, and 1% Triton X-100) containing protease inhibitor cocktail (5056489001, Sigma) and 1 mM DNase I. Chromatin was digested for 2 h at 4 °C and centrifuged at 10,000g for 10 min. The supernatant was then incubated with specific antibodies (Supplementary Table 3) and immune complexes were collected using Dynabeads Protein A/G (ThermoFisher). For silver staining, samples were run on a 5%–20% Bis-Tris SDS–PAGE gel (BioRad) and stained with SilverQuest Silver Staining kit (Invitrogen) according to the manufacturer’s instructions. For immunoblotting, beads were boiled for 5 min in SDS loading buffer. For immunoprecipitations in Fig. 1f, g, Benzonase (Sigma, E8263) treatment was performed by adding 500 U ml^−1^ benzonase to cell lysates followed by incubation for 1 h in 4 °C before incubation with antibody immobilized beads. For mass spectrometry analysis, proteins were eluted with 0.5 M NH_4_OH and dried to completion in a speed vac.

For Flag–NOL9 and Flag–WDR18 immunoprecipitation and mass spectrometry, dried protein samples were digested in 200 mM EPPS buffer pH 8.5 with trypsin (Promega V5111). Digests contained 2% acetonitrile (v/v) and were performed at 37 °C overnight. Digests were labelled directly with TMT10 plex reagents (ThermoFisher Scientific, 90406). Labelling efficiency was checked by mass spectrometry. After hydroxylamine-quenching (0.3% v/v) for 15 min, reactions were mixed and acidified and solvent evaporated to near completion by speed vac. Samples were then fractionated by alkaline reversed phase chromatography (ThermoFisher 84868) into 12 fractions eluted with 10%, 12.5%, 15%, 17.5%, 20%, 25%, 30%, 35%, 40%, 50%, 65% and 80% acetonitrile. Fractions were pooled into 6 fractions (1+7, 2+8, 3+9, 4+10, 5+11, 6+12), dried down, stage-tipped and analysed by mass spectrometry on an Orbitrap Lumos instrument (Thermo Scientific). Relative quantification followed a multi-notch SPS-MS^3^ method. Prior to injection, peptides were separated by HPLC with an Easy-nLC 1200 liquid chromatography system using 100 μm inner diameter capillaries and a C_18_ matrix (2.6 μM Accucore C_18_ matrix, ThermoFisher Scientific). Peptides were separated with 4-hour acidic acetonitrile gradients. MS^1^ scans were measured by orbitrap recording (resolution 120,000, mass range 400–1400 Th). After collision induced dissociation (CID) (35%), MS^2^ spectra were collected by iontrap mass analyser. After SPS (synchronous precursor selection), TMT reporter ions were generated by high-energy collision-induced dissociation (HCD) (55%) and quantified by orbitrap MS^3^ scan (resolution 50,000 at 200 Th). Spectra were searched with an in-house written software based on Sequest (v.28, rev. 12) against a forward and reversed human proteome database (Uniprot 07/2014). Mass tolerance for searches was 50 ppm for precursors and 0.9 Da for fragment ions. Two missed tryptic cleavages per peptide were allowed and oxidized methionine (+15.9949 Da) was searched dynamically. For a peptide FDR (false discovery rate) of 1%, a decoy database strategy and linear discriminant analysis (LDA) were applied. The FDR for collapsed proteins was 1%. Proteins were quantified by summed peptide TMT s/n (signal/noise) with a sum s/n > 200 and an isolation specificity of >70%. Details of the TMT workflow and sample preparation procedures were described recently^55^.

For Flag–PHC2 and Flag–CBX4 immunoprecipitation and mass spectrometry, we added 20 μl of 8 M urea, 100 mM EPPS pH 8.5 to the beads. We added 5 mM TCEP and incubated the mixture for 15 min at room temperature. We then added 10 mM of iodoacetamide for 15 min at room temperature in the dark. We added 15 mM DTT to consume any unreacted iodoacetamide. We added 180 μl of 100 mM EPPS pH 8.5. to reduce the urea concentration to <1 M, 1 μg of trypsin, and incubated at 37 °C for 6 h. The solution was acidified with 2% formic acid and the digested peptides were desalted via StageTip, dried via vacuum centrifugation, and reconstituted in 5% acetonitrile, 5% formic acid for LC-MS/MS processing. All label-free mass spectrometry data were collected using a Q Exactive mass spectrometer (Thermo Fisher Scientific) coupled with a Famos Autosampler (LC Packings) and an Accela600 liquid chromatography (LC) pump (Thermo Fisher Scientific). Peptides were separated on a 100 μm inner diameter microcapillary column packed with about 20 cm of Accucore C18 resin (2.6 μm, 150 Å, Thermo Fisher Scientific). For each analysis, we loaded about 2 μg onto the column. Peptides were separated using a 1 h method from 5 to 29% acetonitrile in 0.125% formic acid with a flow rate of about 300 nl min^−1^. The scan sequence began with an Orbitrap MS1 spectrum with the following parameters: resolution 70,000, scan range 300–1,500 Th, automatic gain control (AGC) target 1 × 10^5^, maximum injection time 250 ms, and centroid spectrum data type. We selected the top twenty precursors for MS2 analysis which consisted of HCD high-energy collision dissociation with the following parameters: resolution 17,500, AGC 1 × 10^5^, maximum injection time 60 ms, isolation window 2 Th, normalized collision energy (NCE) 25, and centroid spectrum data type. The underfill ratio was set at 9%, which corresponds to a 1.5 × 10^5^ intensity threshold. In addition, unassigned and singly charged species were excluded from MS2 analysis and dynamic exclusion was set to automatic. Mass spectrometric data analysis. Mass spectra were processed using a Sequest-based in-house software pipeline. MS spectra were converted to mzXML using a modified version of ReAdW.exe. Database searching included all entries from the *S. pombe* UniProt database which was concatenated with a reverse database composed of all protein sequences in reversed order. Searches were performed using a 50 ppm precursor ion tolerance. Product ion tolerance was set to 0.03 Th. Carbamidomethylation of cysteine residues (+57.0215 Da) were set as static modifications, while oxidation of methionine residues (+15.9949 Da) was set as a variable modification. Peptide spectral matches (PSMs) were altered to a 1% FDR. PSM filtering was performed using a linear discriminant analysis, as described previously, while considering the following parameters: XCorr, ΔCn, missed cleavages, peptide length, charge state, and precursor mass accuracy. Peptide-spectral matches were identified, quantified, and collapsed to a 1% FDR and then further collapsed to a final protein-level FDR of 1%. Furthermore, protein assembly was guided by principles of parsimony to produce the smallest set of proteins necessary to account for all observed peptides.

### GST pulldown and immunoblotting

Proteins for GST pulldown assays were expressed in BL21 Codon Plus *Escherichia coli* (Agilent Technologies) with 200 μM IPTG induction at 16 °C overnight. Bacteria were then collected and washed with cold PBS, and sonicated (Branson sonicator) for 1 min with 20% amplitude at 4 °C. Sonicated samples were centrifuged at 20,000*g* for 10 min, and the supernatant was added to 0.5 ml Glutathione Sepharose 4B resin (GE Healthcare, 17075605), which was equilibrated with PBS. GST-tagged proteins were incubated with the resin for 2 h at 4 °C. The resin was then washed 6 times with PBS containing 1% Triton 100. To remove the GST tag, bead-coupled proteins were incubated with PreScission Protease (GE Healthcare, 27-0843-01) in reaction buffer (50 mM Tris-HCl, Ph7.0, 150 mM NaCl, 1 mM EDTA, 1 mM DTT) for 2 h at 4 °C. The GST-tagged PreScission Protease was removed using Glutathione Sepharose 4B resin.

For GST pulldown assays, 10 μl 50% slurry of Glutathione Sepharose 4B was used for each sample. GST or GST-tagged proteins (0.1 μM) were incubated with untagged proteins (0.1 μM) in 1 ml PBS (137 mM NaCl, 2.7 mM KCl, 8 mM Na_2_HPO_4_, and 2 mM KH2PO4, Ph7.4) containing 0.5% Triton 100 overnight at 4 °C. Beads were washed 3 times with PBS containing 0.5% Triton 100, resuspended in SDS protein buffer, and boiled for 5 min. Input (2–5%) and bound proteins (10–50%) were run on 4–20% gradient SDS–PAGE gel. SDS–PAGE was performed to separate proteins for 2 h at 80 V, and proteins were transferred to a PVDF membrane (Millipore). The membranes were blocked in 3% milk in PBS with 0.2% Tween-20, and sequentially incubated with primary antibodies and HRP-conjugated secondary antibodies, or directly incubated with HRP-conjugated primary antibodies for chemiluminescence detection. Sources of antibodies can be found in Supplementary Table 3.

### Sucrose gradient centrifuge fractionation assay

Flag-tagged proteins were purified from the soluble chromatin fraction using magnetic beads (Sigma, M8823) and eluted with 3×Flag peptides (APExBIO, A6001) in elution buffer (20 mM Hepes-KOH, pH7.5, 100 mM KOAc, 5 mM Mg(OAc)_2_, 1 mM EDTA, 10% Glycerol). Sucrose gradients (10%-30%) were prepared using the Gradient station (BIOCOMP). An Optima TLX Ultracentifuge equipped with TLS-55 rotor was used for ultracentrifugation for 16 h at 4 °C with 35 k rpm. Gradients of 2.2 ml were fractionated into 22 fractions. One-hundred-microlitre fractions were pipetted from top and protein in fractions was captured using StrataClean resin (Agilent, 400714). Protein samples were boiled in SDS sample buffer (62.5 mM Tris-HCl, pH 6.8, 2% SDS, 10% glycerol, 0.01% bromophenol blue) for 3 min at 98 °C, and analysed by immunoblotting following gel electrophoresis (4%–15% precast protein gel with SDS from Biorad, 4561081).

### RT–qPCR

Total RNA was extracted using the RNeasy Plus kit (74134, Qiagen) and reverse transcribed into cDNA using gene-specific primers and reverse transcription kit (18090010, ThermoFisher). cDNA was analysed by running PCR on a QuantStudio 7 Flex Real Time PCR System (Applied Biosystem). All reactions were performed using 10 ng RNA in a final volume of 10 μl. PCR parameters were 95 °C for 2 min and 40 cycles of 95 °C for 15 s, 60 °C for 15 s, and 72 °C for 15 s, followed by 72 °C for 1 min. All the quantitative PCR data presented were at least three biological replicates. The forward and reverse primers used for RT–qPCR targeted the first exons of the genes. Primer sequences are presented in Supplementary Table 4.

### RNA-seq

Total RNA was isolated from human cells with an RNA purification kit (Qiagen, 74134) and genomic DNA was removed by genomic DNA binding columns in the kit. Two micrograms of total RNA was used for RNA-seq library construction. Poly(A)-containing mRNA was isolated by poly(A) selection beads and further reverse transcribed to cDNA. The resulting cDNA was ligated with adapters, amplified by PCR, and further cleaned to obtain the final library. Libraries were sequenced on an Illumina Hiseq machine (Novogene) to obtain 150 bp paired-ended reads.

RNA-seq reads were pseudo aligned using Kallisto 0.45.1. An index was generating using the Ensembl hg19 GTF and cDNA FASTA. Kallisto was run using default parameters with two exceptions: allowing searching for fusions (−fusion) and setting bootstrap to 100 (−b 100).

To visualize the mapped RNA-seq with IGV or UCSC genome browser, bam files were generated with Hisat 2.2.0, which was followed by making bigwig files with deeptools (v/3.0.2) (binsize 10). Reads were normalized to reads per genome coverage.

Read counts were calculated on a per transcript basis using Kallisto and the above described pseudoalignment. The R package tximport 1.10.1 was used to select the dominant transcript per gene (txOut = FALSE), which was then used for DEseq2 analysis. To analyse only active genes, those with 0 read counts in all samples were removed from the DEseq2 output. As they are not transcribed by PolII, 13 genes on chrM were also removed, resulting in a list of 24,043 active genes. Upregulated genes and downregulated genes are defined with *P*_adj_ < 0.05 and fold change > 2 or < −2.

### PRO-seq library construction

Aliquots of frozen (−80 °C) permeabilized cells were thawed on ice and pipetted gently to fully resuspend. Aliquots were removed and permeabilized cells were counted using a Luna II, Logos Biosystems instrument. For each sample, 1 million permeabilized cells were used for nuclear run-on, with 50,000 permeabilized *Drosophila* S2 cells added to each sample for normalization. Nuclear run on assays and library preparation were performed essentially as described^56^ with modifications noted: 2× nuclear run-on buffer consisted of (10 mM Tris (pH 8), 10 mM MgCl2, 1 mM DTT, 300 mM KCl, 40 μM each biotin-11-NTPs (Perkin Elmer), 0.8 U μl^−1^ SuperaseIN (Thermo), 1% sarkosyl). Run-on reactions were performed at 37 °C. Adenylated 3′ adapter was prepared using the 5′ DNA adenylation kit (NEB) and ligated using T4 RNA ligase 2, truncated KQ (NEB, per manufacturer’s instructions with 15% PEG-8000 final) and incubated at 16 °C overnight. One-hundred-eighty microlitres of betaine blocking buffer (1.42 g of betaine brought to 10 ml with binding buffer supplemented to 0.6 μM blocking oligonucleotide (TCCGACGATCCCACGTTCCCGTGG/3InvdT/)) was mixed with ligations and incubated 5 min at 65 °C and 2 min on ice prior to addition of streptavidin beads. After T4 polynucleotide kinase (NEB) treatment, beads were washed once each with high salt, low salt, and blocking oligonucleotide wash (0.25× T4 RNA ligase buffer (NEB), 0.3 uM blocking oligonucleotide) solutions and resuspended in 5′ adapter mix (10 pmol 5′ adapter, 30 pmol blocking oligonucleotide, water). 5′ adapter ligation was per Reimer but with 15% PEG-8000 final. Eluted cDNA was amplified with five cycles (NEBNext Ultra II Q5 master mix (NEB) with Illumina TruSeq PCR primers RP-1 and RPI-X) following the manufacturer’s suggested cycling protocol for library construction. A portion of preCR was serially diluted and for test amplification to determine optimal amplification of final libraries. Pooled libraries were sequenced using the Illumina NovaSeq platform.

### PRO-seq data analysis

All custom scripts described herein are available on the Adelman Lab Github (https://github.com/AdelmanLab/NIH_scripts). Using a custom script (trim_and_filter_PE.pl), FASTQ read pairs were trimmed to 41 bp per mate, and read pairs with a minimum average base quality score of 20 retained. Read pairs were further trimmed using cutadapt 1.14 to remove adapter sequences and low-quality 3′ bases (−match-read-wildcards -m 20 -q 10). R1 reads, corresponding to RNA 3′ ends, were then aligned to the spiked in Drosophila genome index (dm3) using Bowtie 1.2.2 (-v2-p6–best–un), with those reads not mapping to the spike genome serving as input to the primary genome alignment step (using Bowtie 1.2.2 options -v2–best). Reads mapping to the hg19 reference genome were then sorted, via samtools 1.3.1 (−n), and subsequently converted to bedGraph format using a custom script (bowtie2stdBedGraph.pl). Because R1 in PRO-seq reveals the position of the RNA 3′ end, the ‘+’ and ‘−’ strands were swapped to generate bed-Graphs representing 3′ end position at single nucleotide resolution.

For NOL9 KD PRO-seq, we performed 2 sets of PRO-seq experiments, each with two biological replicates. In the first set of experiments, NOL9 depletion resulted in many more upregulated (228) than downregulated (30) genes, while in the second set experiments, nearly the same number of genes were up (162) and down (160) regulated. Furthermore, unlike the first set, in the second set, the extent of overlap between siNOL9 upregulated and downregulated genes with those upregulated in EED-KO or RING1A/B-DKO was similar. Although the basis of this discrepancy is unclear, the correlation between the two biological replicates in Set2 was lower than Set1 raising the possibility that poor growth or inefficient NOL9 depletion in Set2 siNOL9 cells may have resulted in a larger number of non-specifically downregulated genes. We therefore eliminated the Set2 siNOL9 data and used only the 2 biological replicates from the Set1 siNOL9 experiment.

### Gene model refinement using PRO-seq and RNA-seq

To select gene-level features for differential expression analysis, as well as for pairing with PRO-seq data, we assigned a single, dominant TSS and transcription end site (TES) to each active gene. This was accomplished using a custom script, get_gene_annotations.sh (available at https://github.com/AdelmanLab/GeneAnnotationScripts), which uses RNA-seq read abundance and PRO-seq R2 reads (RNA 5′ ends) to identify dominant TSSs, and RNA-seq profiles to define most commonly used TESs. RNA-seq and PRO-seq data from control and siNOL9 cells were used for this analysis, to capture gene activity under both conditions. Exon- and transcript-level features consistent with the resulting TSS to TES windows for 21,004 active genes in HEK 293T cells were selected from an hg19 reference GTF (GRCh38.99 from Ensembl). This filtered list of active genes was used for analyses shown in Figs. 2c-e, 4a-d, Extended Data Figs, 2c, d, 6b-k, as well as for defining differentially expressed genes in PRO-seq data. Differentially expressed genes between control (*n* = 2) and siNOL9 (*n* = 2) cells were determined using DESeq2 v1.26.0. Genes were called as differentially expressed using DEseq2’s DESeqDataSetFromMatrix mode at an adjusted *P* value threshold of <0.05 and fold change >1.5. This revealed 228 genes to be upregulated and 30 genes to be downregulated upon siNOL9.

### ChIP–qPCR, ChIP–seq and data analysis of ChIP–seq

ChIP was performed as previously described with minor modifications^57^. Cells for ChIP were cultured in 15 cm plates. Cell were first washed with cold PBS, crosslinked at room temperature with 10 mM DMP (ThermoFisher Scientific) for 30 min, and then 1% formaldehyde (ThermoFisher Scientific) for 15 min. Crosslinking reactions were quenched by addition of 125 mM glycine for 5 min. Crosslinked cells were separated by 3 min treatment of 0.05% trypsin (Gibco), and then washed with cold PBS 3 times. In every wash, cells were centrifuged for 3 min at 1,000g at 4 °C. Cell were then resuspended in sonication buffer (pH 7.9, 50 mM Hepes, 140 mM NaCl, 1 mM EDTA, 1% Triton, 0.1% Sodium deoxycholate, and 0.5% SDS) and sonicated to shear chromatin into ~300 bp fragments using a Branson sonicator. Sonicated samples were diluted fivefold with ChIP dilution buffer (pH 7.9, 50 mM Hepes, 140 mM NaCl, 1 mM EDTA, 1% Triton, 0.1% Sodium deoxycholate) to obtain a final concentration of 0.1% SDS. Diluted samples were centrifuged at 13,000 rpm for 10 min. The supernatant was pre-cleared with protein A/G or Dynabeads M-280 Streptavidin beads (ThermoFisher) and immunoprecipitated for 3–12 h using 3 μg antibodies and 40 μl protein A/G or Dynabeads M-280 Streptavidin beads. The beads were washed twice with high salt wash buffer A (pH 7.9, 50 mM Hepes, 500 mM NaCl, 1 mM EDTA, 1% Triton, 0.1% Sodium deoxycholate, and 0.1% SDS), and once with wash buffer B (pH 7.9, 50 mM Hepes, 250 mM LiCl, 1 mM EDTA, 1% Triton, 0.1% Sodium deoxycholate, 0.5% NP-40). The bound chromatin fragments were eluted with elution buffer (pH 8.0, 50 Mm Tris, 10 mM EDTA, 1% SDS) for 5 min at 65 °C. Eluted DNA-proteins complexes were treated with RNase A and crosslinks were reversed overnight at 65 °C. Proteinase K was then added to digest proteins for 1 h at 55 °C. DNA was further purified using PCR Purification Kit (QIAGEN) and analysed by PCR on a QuantStudio 7 Flex Real Time PCR System (Applied Biosystem). PCR parameters were 95 °C for 2 min and 40 cycles of 95 °C for 15 s, 60 °C for 15 s, and 72 °C for 15 s, followed by 72 °C for 1 min. All the ChIP–qPCR data presented were at least three biological replicates. Primer sequences are in Supplementary Table 4. Error bars represent standard deviation (three biological replicates).

For ChIP–seq, sequencing library was constructed using TruSeq DNA sample Prep Kits (Illumina) and adapter dimers were removed by agarose gels electrophoresis. Sized selected and purified DNA libraries were sequenced on an Illumina Hiseq 2500 machine (Bauer core facility at Harvard University) to obtain 50 bp single-ended reads. ChIP–seq reads were quality controlled with fastqc (v0.11.5) and mapped to the human genome reference (GRCh37/hg19) using bowtie2 (v2.2.9) with default parameters or bowtie (v1.2.2) with parameters -v2-k1–best. Bam files were generated with samtools 1.3.1, which was followed by making bigwig files with deeptools (v/3.0.2) (binsize 10). Reads were normalized to Reads Per Genome Coverage (RPGC) with deeptools (v/3.0.2) bamCoverage function. To analyse read density at TSS regions, we made heatmaps and metaplots of ChIP–seq samples. TSS was centered in the regions plotted and data were tabulated with the same distance relative to TSS. Matrix files were generated using computematrix function of deeptools (v/3.0.2). Based on generated matrix file, heatmaps were generated by PlotHeatmap function, and profiles were generated by plotprofile function or in Prism.

To analyse read density and correlation between different ChIP–seq samples, we performed Spearman correlation analysis. Reads density was analysed at all hg19 annotated TSSs (*n* = 56,335) with multiBigwig-Summary function from deeptools (v/3.0.2) to get a npz matrix file. The heatmap Spearman of Pearson correlation was generated by plot-Correlation function of deeptools (v/3.0.2). The heatmaps generated in this study also included all annotated human genes (hg19). The gene list was obtained from https://genome.ucsc.edu. Promoter regions were defined as ±2 kb from TSSs. Peak overlaps were analysed by bedtools (v/3.0.2) intersect function.

For co-occupany analysis in Extended Data Fig. 2, peak calling of TEX10, H2AK119ub1, and H3K9me3 was performed with MACS2 (2.1.1.20160309) with Input ChIP–seq sample as control (−p 0.05–broad,–broad-cutoff 0.05, FoldChang>2.5, Length>800 bp).

For defining TEX10-bound targets in Fig. 2, TEX10 peaks were called using HOMER (version 4.9) with the -style histone option and siTEX10 ChIP–seq as background. TEX10-bound genes were defined as those that had 50 or more TEX10 reads in the TSS ±1 kb region (*n* = 7,827); all others were considered unbound (*n* = 13,177).

For defining Polycomb target genes in Figs. 2, 3, H2AK119ub1 ChIP–seq data from HEK 293FT cells were used. Deeptools was used to count reads in TSS ±2 kb regions. *K*-means clustering was performed with *k* = 2. Cluster one was H2AK119ub1 enriched and counted as Polycomb target genes. Venn diagrams in Extended Data Fig. 8 were made based on the number of overlapping target genes. Deeptools was used to count reads in TSS ±2 kb regions. *K*-means clustering was performed with a fixed value of *k* = 3. Cluster one was counted as target genes.

The sources of ChIP–seq data used in this study are listed in Supplementary Table 5.

### Statistical tests

For RNA-seq, PRO-seq and ChIP–seq, statistical significance for comparisons was assessed by Wilcoxon (unpaired) or Mann–Whitney (pairwise) tests. The test used and error bars are defined in each figure legend.

Significance for immunostaining foci was evaluated using unpaired two-tail student’s *t*-test. All the RT–qPCR and ChIP–qPCR data are represented as mean ± s.d. using GraphPad Prism 8 software. Volcano plots of Mass spec results were made with Microsoft Excel.

## Extended Data

## Supplementary Material

Supplementary Tables and Figure 1

Supplementary Table 7

Supplementary Table 8

## Data Availability

The raw mass spectrometry data were deposited with accession number PXD027966 and PXD029403. The raw and processed high-throughput sequencing data have been deposited at NCBI Gene Expression Omnibus under accession GSE175678. Source data are provided with this paper.
