# Supplementary material for: Rixosomal RNA degradation contributes to silencing of Polycomb target genes
Source: Nature. 2022 Mar 30;604(7904):167–74. doi: 10.1038/s41586-022-04598-0 (PMC8986528; doi:10.1038/s41586-022-04598-0)

nature portfolio

https://doi.org/10.1038/s41586-022-04598-0

Supplementary information

Rixosomal RNA degradation contributes to silencing of Polycomb target genes

In the format provided by the authors and unedited

nature portfolio

https://doi.org/10.1038/s41586-022-04598-0

Supplementary information

Rixosomal RNA degradation contributes to silencing of Polycomb target genes

In the format provided by the authors and unedited

Supplementary Tables 1-6 and Supplementary Figure 1

Rixosomal RNA degradation contributes to silencing of Polycomb target genes

Haining Zhou[1], Chad B. Stein[2], Tiasha A. Shafiq[1], Gergana Shipkovenska[1], Marian Kalocsay[3], Joao A. Paulo[3], Jiuchun Zhang[4], Zhenhua Luo[5], Steven P. Gygi[3], Karen Adelman[2], Danesh Moazed[1*]

[1]Howard Hughes Medical Institute, Department of Cell Biology, Harvard Medical School, Boston, MA, USA.

[2]Department of Biological Chemistry and Molecular Pharmacology, Blavatnik Institute, Harvard Medical School, Boston, MA, USA.

[3]Department of Cell Biology, Harvard Medical School, Boston, MA, USA.

[4]Initiative for Genome Editing and Neurodegeneration, Department of Cell Biology, Blavatnik Institute, Harvard Medical School, Boston, MA, USA.

[5]Precision Medicine Institute, the First Affiliated Hospital, Sun Yat-sen University, Guangzhou, China

*Correspondence: danesh@hms.harvard.edu

Supplementary Table 1. List of siRNA sequences used in this study.

**siRNA**	**sequence**	**SOURCE**
negative control	5'-UUCUCCGAACGUGUCACGU-3'	This study
NOL9	5'-AGACCUAAGUUCUGUCGAA-3'	This study
SUV39H1	5'-UCUUGUGGCAAAGAAAGCGAUGCGG-3'	Reference [1]
WDR18	5'-AGAUCAAUCGGGACCUGUU-3'	This study
MDN1	5'-GGAAUGCCGAAGCCAUUAA-3'	Reference [2]
XRN2-1	5'-CCAGUAAACCUAAUCCAAA-3'	This study
XRN2-2	5'-GCAGAUGCUGAUCUCAUUA-3'	This study
SENP3	5'-GGGAGUCCCUUCCCAAGAA-3'	This study
LAS1L	5'-CAUUUAUACCCAGAGUGGA-3'	This study
TEX10	5'-GCAGUUCAACUUCUUCAAU-3'	This study
EZH2-1	5'-GCUCUAGACAACAAACCUU-3'	This study
EZH2-2	5'-GCUCUAGACAACAAACCUU-3'	This study
PELP1	5'-GCACUGUGUGUCUUGGCUU-3'	This study
RING1A	5'-CCUGAUCUCUAAGAUCUAU-3'	This study
RING1B-1	5'-GCUCAUCAAGAGAGAGUAU-3'	This study
RING1B-2	5'-CCUACAAAGGAGCACAAAU-3'	This study
NPM1	5'-GAAUUGCUUCCGGAUGACU-3'	Reference [3]
PES1	5'-CCAGAGGACCUAAGUGUGA-3'	Reference [4]

Supplementary Table 2. List of oligonucleotides used for CRISPR/Cas9 genome tagging editing in this study.

Oligo	sequence	Source	Application
NOL9 sgRNA	GCCCTGAGGACCC AGCATGG	This study	Tagging
WDR18 sgRNA	GTGGGGAAGGCA AGATGG	This study	Tagging
PHC2 sgRNA	AGCATGCTCAAGG ACTCCTA	This study	Tagging
NOL9-Forward primer	GGCCCTGAGGACC CAGCAT	This study	genotyping
NOL9-Reverse primer	GGATTCGAGT TCGGGTTCGG	This study	genotyping
WDR18-Forward primer	TCGTCCGCGTCTC GCTCAT	This study	genotyping
WDR18-Reverse primer	ACTAAGGGTG CACGCGGCC	This study	genotyping
PHC2-Forward primer	AAG CCC TGC TGC TGC TCA AG	This study	genotyping
PHC2-Reverse primer	TCCAACC GGCCCCATTT CTG	This study	genotyping

Supplementary Table 3. List of antibodies used in this study.

Antibodies	Source	Cat#	Application
Anti-RING1B	Cell Signaling Technology	5694S	ChIP, 1:200
Anti-CBX2	Proteintech	15579-1-AP	IB, 1:500
Anti-RING1B	Genetex	GTX106431	IB, 1:300
Anti-RING1B	Active Motif	39664	IP, 1:50
Anti-PHC2	Elabscience	E-AB-65051	IB, 1:500
Anti-SENP3	Cell Signaling Technology	5591S	IB, 1:1000
Anti-WDR18	Sigma	HPA050193	IB, 1:200; IF, 1:30
Anti-MDN1	Sigma	HPA029666	IB, 1:300; IF, 1:50
Anti-MDN1	Bethyl	A304-739A-T	ChIP, 1: 500
Anti-NOL9	Sigma	SAB4301156	IB, 1:200;
Anti-Actin	Abcam	mAbcam8224	IB, 1:500
Anti-H3K9me3	Diagenode	C15500003-50	ChIP, 1:1000
Anti-XRN2	Proteintech	11267-1-AP	IB, 1:500
Anti-Flag	Sigma	F3165	IP, 1:100
Anti-Flag M2-Peroxidase	Sigma	A8592	IB, 1:10000
Anti-EED	Millipore	17-10034	IB, 1:200
Anti-EZH2	Millipore	17-662	IB, 1:300, IF, 1:50

Anti-TEX10	ThermoFisher	720257	IB, 1:500; ChIP, 1:500
Anti-LAS1L	Proteintech	16010-1-AP	IB, 1:500
Anti-GAPDH	Abcam	Ab181603	IB, 1:1000
Anti-SUZ12	Millipore	17-661	IB, 1:500
Anti-H3K27me3	Millipore	17-622	ChIP, 1:1000
Anti-H2AK119ub1	Cell Signaling Technology	8240T	ChIP, 1:2000
Anti-RYBP	Proteintech	11365-1-AP	IB, 1:100
Anti-YAF2	Genetex	GTX115355	IB, 1:100
Anti-PCGF6	Proteintech	24103-1-AP	IB, 1:500
Anti-NPM1	Proteintech	60096-1-lg	IB, 1:1000; IF, 1:500
Anti-BMI1	Proteintech	10832-1-AP	IB, 1:1000
Anti-PELP1	Novus bio	NB-110-40622	IB, 1:500

Supplementary Table 4. List of oligonucleotide sequences for RT-qPCR, and ChIP-qPCR used in this study.

**Primers**	**Sequence**	**Application**
HUMAN GAPDH-F	AACAGCCTCAAGATCATCAGC	ChIP
HUMAN GAPDH-R	GGATGATGTTCTGGAGAGCC	ChIP
HUMAN PCDH10-F	GGATGGCAACCGATTCGCTGA	ChIP, RT
HUMAN PCDH10-R	ACCTCCTCCG TCCACCGCGG T	ChIP, RT
HUMAN BETA ACTIN-R	ATGTCCACGTCACACTTCAT	RT
HUMAN BETA ACTIN-F	AGAGCTACGAGCTGCCTGAC	RT
HUMAN THBD-F	ACAGTCGTCT TGTTACAGGG	ChIP
HUMAN THBD-R	AGTAAACCCT GCCCTGGCGC	ChIP
HUMAN THBD-F	ATGCTTGGGG TCCTGGTCCT	RT
HUMAN THBD-R	CACTGGCTG CCACCCGGCT	RT
HUMAN SKOR1-F	CCAGAAAGCAAAGTGCAAGGA	ChIP, RT
HUMAN SKOR1-R	CCGGCTCA TATGGGCTAG AA	ChIP, RT
HUMAN PTF1A-F	ATGGACGCGG TGTTGCTGGA	ChIP, RT
HUMAN PTF1A-R	CGTGAAGA CTGGTCGGTG AA	ChIP, RT
HUMAN C11ORF96-F	CAAG CTGCCCAAGG GCCGG	ChIP, RT
HUMAN C11ORF96-R	CTCCACCTCC TGGATCTCGT	ChIP, RT
HUMAN DUSP4-F	AG AGCCTCCGCG AGGACAG	ChIP, RT
HUMAN DUSP4-R	CTTTGAGCAG GCAGATGTCG	ChIP, RT
HUMAN B2M-F	AATATAAGTG GAGGCGTCGC	RT
HUMAN B2M-R	GAAAGAGAGA GTAGCGCGAG	RT
HUMAN RPS14-F	TCCCACTCTC TCTTTCCGGT	RT
HUMAN RPS14-R	ACAGGGTCCC CTCGCCGCAG	RT
HUMAN ENSA-F	GACACGCAGG AGAAAGAAGG	ChIP, RT
HUMAN ENSA-R	AGTCGGAGCC TCCAGGCTTT	ChIP, RT
HUMAN PES1-F	AACAAGCTGG CGGAGAAGCG	RT
HUMAN PES-R	TCACTCCGGC CTTGCCTTCT	RT
HUMAN NPM1-F	CTATTCAAG ATCTCTGGCA G	RT
HUMAN NPM1-R	CAACTGTTAC AGAAATGAAA	RT

HUMAN IGFBP3-F	GGGGCTTCTG CTGGTGTGTG	ChIP, RT
HUMAN IGFBP3-R	CTACTTGCTC TGCATGCTGT	ChIP, RT
HUMAN INSM1-F	GTGTGCGGAG AGTCGTTCGC	ChIP, RT
HUMAN INSM1-R	AGGTGGCCGG GCAGTACTTG	ChIP, RT
Citrine-F	CAAGGGCGA GGAGCTGTTC	RT
Citrine-R	CACGCTGAA CTTGTGGCCG	RT
P3-F	CGCCTTTTT CCCGAGGGTG	ChIP
P3-R	GTGTTCTGG CGGCAAACCC	ChIP
P2-F	ACGTATGTCGAGGTAGGCGT	ChIP
P2-R	CTAGGCACCGGTTCAATTGC	ChIP
P1-F	GGAATTCCAGATGGTGCGCT	ChIP
P1-R	GGCCAGAGCAGATACGTAGG	ChIP
P4-F	TGCATAAACGTTGTCGCCATT	ChIP
P4-F	AAGTGCGCCCTTCGAGTAAG	ChIP

RT, RT-pPCR.

Supplementary Table 5 . Sources and accession numbers for Next-generation sequencing data used in this study.

ChIP-seq	Identifier	Source
Human embryonickidney 293 H3K4me4	GSM897574	Reference [5]
Human embryonickidney 293 H3K36me3	GSM1948534	Reference [6]
Human embryonickidney 293 H3K79me3	GSM2357976	Reference [7]
Human ES H3K9me3	GSM1528888	Reference [8]
Human ES H3K27me3	GSM1528885	Reference [8]
Human ES H3K4me4	GSM616128	Reference [9]
Human ES H3K79me3	GSM3027439	Reference [10]
**ChIP-seq of this study**	**Identifier**	**Source**
INPUT_siMDN1_rep1	GSM4239937	This study
INPUT_siMDN1_rep2	GSM4239938	This study
INPUT_siNC_rep1	GSM4239939	This study
INPUT_siNC_rep2	GSM4239940	This study
INPUT_siNOL9_rep1	GSM4239941	This study
INPUT_siNOL9_rep2	GSM4239942	This study
H3K9me3_rep1	GSM4239943	This study
H3K9me3_rep2	GSM4239944	This study
H3K27me3_siNC_rep1	GSM4239945	This study
H3K27me3_siNC_rep2	GSM4239946	This study
H3K27me3_siNOL9_rep1	GSM4239947	This study
H3K27me3_siNOL9_rep2	GSM4239948	This study
MDN1_siMDN1_rep1	GSM4239949	This study
MDN1_siMDN1_rep2	GSM4239950	This study
MDN1_siNC_rep1	GSM4239951	This study
MDN1_siNC_rep2	GSM4239952	This study

H2AK119ub1-HEK293FT_rep1	GSM4502558	This study
H2AK119ub1-HEK293FT_rep2	GSM4502559	This study
H2AK119ub1-HEK293FT_siNOL9_rep1	GSM5343681	This study
H2AK119ub1-HEK293FT_siNOL9_rep2	GSM5343682	This study
MDN1-HEK293FT_RING1DKO_rep1	GSM5343683	This study
MDN1-HEK293FT_RING1DKO_rep2	GSM5343684	This study
TEX10-HEK293FT_siNC_rep1	GSM5343685	This study
TEX10-HEK293FT_siNC_rep2	GSM5343686	This study
TEX10-HEK293FT_siTEX10_rep1	GSM5343687	This study
TEX10-HEK293FT_siTEX10_rep2	GSM5343688	This study
TEX10-HEK293FT_RING1DKO_rep1	GSM5343689	This study
TEX10-HEK293FT_RING1DKO_rep2	GSM5343690	This study
H2AK119ub1-HeLa_rep1	GSM5343691	This study
H2AK119ub1-HeLa_rep2	GSM5343692	This study
H3K27me3-HeLa_rep1	GSM5343693	This study
H3K27me3-HeLa_rep2	GSM5343694	This study
INPUT-HeLa_rep1	GSM5343695	This study
INPUT-HeLa_rep2	GSM5343696	This study
MDN1-HeLa_siMDN1_rep1	GSM5343697	This study
MDN1-HeLa_siMDN1_rep2	GSM5343698	This study
MDN1-HeLa_siNC_rep1	GSM5343699	This study
MDN1-HeLa_siNC_rep2	GSM5343700	This study
H2AK119ub1-ES_rep1	GSM5343673	This study
H2AK119ub1-ES_rep2	GSM5343674	This study
INPUT-ES_rep1	GSM5343675	This study
INPUT-ES_rep2	GSM5343676	This study

TEX10-ES_rep1	GSM5343677	This study
TEX10-ES_rep2	GSM5343678	This study
TEX10-ES_siTEX10_rep1	GSM5343679	This study
TEX10-ES_siTEX10_rep2	GSM5343680	This study
H2AK119ub1_HEK293FT_RING1BQ137AQ138A_rep1	GSM5659334	This study
H2AK119ub1_HEK293FT_RING1BQ137AQ138A_rep2	GSM5659335	This study
H2AK119ub1_HEK293FT_WT_rep1	GSM5659336	This study
H2AK119ub1_HEK293FT_WT_rep2	GSM5659337	This study
RING1B_HEK293FT_WT_rep1	GSM5659338	This study
RING1B_HEK293FT_WT_rep2	GSM5659339	This study
RING1B_HEK293FT_RING1BQ137AQ138A_rep1	GSM5659340	This study
RING1B_HEK293FT_RING1BQ137AQ138A_rep2	GSM5659341	This study
**RNA-seq and PRO-seq of this study**	**Identifier**	**Source**
RING1AB_KO_no_siRNA_RNAseq_HEK293FT_rep1	GSM5394422	This study
RING1AB_KO_no_siRNA_RNAseq_HEK293FT_rep2	GSM5394423	This study
RING1B_Q1371Q138A_RNAseq_HEK293FT_rep1	GSM5394424	This study
RING1B_Q1371Q138A_RNAseq_HEK293FT_rep2	GSM5394425	This study
WT_RNAseq_HEK293FT_rep1	GSM5394426	This study
WT_RNAseq_HEK293FT_rep2	GSM5394427	This study
EED_KO_RNAseq_HEK293FT_rep1	GSM5343705	This study
EED_KO_RNAseq_HEK293FT_rep2	GSM5343706	This study
EZH_KO_RNAseq_HEK293FT_rep1	GSM5343707	This study
EZH_KO_RNAseq_HEK293FT_rep2	GSM5343708	This study

RING1AB_KO_RNAseq_HEK293FT_rep1	GSM5343709	This study
RING1AB_KO_RNAseq_HEK293FT_rep2	GSM5343710	This study
siEZH2_RNAseq_HEK293FT_rep1	GSM5343711	This study
siEZH2_RNAseq_HEK293FT_rep2	GSM5343712	This study
siNC_RNAseq_HEK293FT_rep1	GSM5343713	This study
siNC_RNAseq_HEK293FT_rep2	GSM5343714	This study
siNOL9_RNAseq_HEK293FT_rep1	GSM5659344	This study
siNOL9_RNAseq_HEK293FT_rep2	GSM5659345	This study
siLAS1L_RNAseq_HEK293FT_rep1	GSM5659342	This study
siLAS1L_RNAseq_HEK293FT_rep2	GSM5659343	This study
siRING1B_RNAseq_HEK293FT_rep1	GSM5343717	This study
siRING1B_RNAseq_HEK293FT_rep2	GSM5343718	This study
RING1A_KO_siRING1B_RNAseq_HeLa_rep1	GSM5343719	This study
RING1A_KO_siRING1B_RNAseq_HeLa_rep2	GSM5343720	This study
siEZH2_RNAseq_HeLa_rep1	GSM5343721	This study
siEZH2_RNAseq_HeLa_rep2	GSM5343722	This study
siLAS1L_RNAseq_HeLa_rep1	GSM5343723	This study
siLAS1L_RNAseq_HeLa_rep2	GSM5343724	This study
siNC_RNAseq_HeLa_rep1	GSM5343725	This study
siNC_RNAseq_HeLa_rep2	GSM5343726	This study
siNOL9_RNAseq_HeLa_rep1	GSM5343727	This study
siNOL9_RNAseq_HeLa_rep2	GSM5343728	This study
siTEX10_RNAseq_HeLa_rep1	GSM5343729	This study
siTEX10_RNAseq_HeLa_rep2	GSM5343730	This study
siNC-proseq-rep-1	GSM4544659	This study
siNC-proseq-rep-2	GSM4544660	This study

siNOL9-proseq-rep-1	GSM4544661	This study
siNOL9-proseq-rep-2	GSM4544662	This study
EED-KO-proseq-rep-1	GSM4544662	This study
EED-KO-proseq-rep-2	GSM4544663	This study
siRING1AB-proseq-rep-1	GSM5343701	This study
siRING1AB-proseq-rep-2	GSM5343702	This study
RING1AB_DKO-proseq-rep1	GSM5343703	This study
RING1AB_DKO-proseq-rep2	GSM5343704	This study
siCtrl-for-RING1AB_KD_DKO-proseq-rep-1	GSM5824569	This study
siCtrl-for-RING1AB_KD_DKO-proseq-rep-2	GSM5824570	This study

Supplementary Table 6. The software and algorithms used for data analysis in this study.

DEseq2 (v1.18.1)	https://doi.org/10.18129/B9.bioc.DESeq2	Reference [11]
deeptools (v3.0.2)	https://github.com/deeptools/	Reference [12]
Bedtools (v2.27.1)	https://github.com/arq5x/bedtools2	Reference [13]
Samtools (v1.3.1)	https://github.com/samtools/samtools	Reference [14]
get_gene_annotations.sh	https://github.com/AdelmanLab/GetGeneAnnotation_GGA	DOI: 10.5281/zenodo.5519928
trim_and_filter_PE.pl	https://github.com/AdelmanLab/NIH_scripts/tree/main/trim_and_filter_PE	10.5281/zenodo.5519915
bowtie2stdBedGraph.pl	https://github.com/AdelmanLab/NIH_scripts/tree/main/bowtie2stdbedgraph	10.5281/zenodo.5519915

1 Murayama, A. *et al.* Epigenetic control of rDNA loci in response to intracellular energy status. *Cell* **133**, 627-639, doi:10.1016/j.cell.2008.03.030 (2008).

2 Raman, N., Weir, E. & Muller, S. The AAA ATPase MDN1 Acts as a SUMO-Targeted Regulator in Mammalian Pre-ribosome Remodeling. *Mol Cell* **64**, 607-615, doi:10.1016/j.molcel.2016.09.039 (2016).

3 Gadad, S. S. *et al.* HIV-1 infection induces acetylation of NPM1 that facilitates Tat localization and enhances viral transactivation. *J Mol Biol* **410**, 997-1007, doi:10.1016/j.jmb.2011.04.009 (2011).

4 Holzel, M. *et al.* The BRCT domain of mammalian Pes1 is crucial for nucleolar localization and rRNA processing. *Nucleic Acids Res* **35**, 789-800, doi:10.1093/nar/gkl1058 (2007).

5 Deplus, R. *et al.* TET2 and TET3 regulate GlcNAcylation and H3K4 methylation through OGT and SET1/COMPASS. *EMBO J* **32**, 645-655, doi:10.1038/emboj.2012.357 (2013).

6 Zhu, K. *et al.* SPOP-containing complex regulates SETD2 stability and H3K36me3-coupled alternative splicing. *Nucleic Acids Res* **45**, 92-105, doi:10.1093/nar/gkw814 (2017).

7 Kang, J. Y. *et al.* KDM2B is a histone H3K79 demethylase and induces transcriptional repression via sirtuin-1-mediated chromatin silencing. *FASEB J* **32**, 5737-5750, doi:10.1096/fj.201800242R (2018).

8 Vallot, C. *et al.* Erosion of X Chromosome Inactivation in Human Pluripotent Cells Initiates with XACT Coating and Depends on a Specific Heterochromatin Landscape. *Cell Stem Cell* **16**, 533-546, doi:10.1016/j.stem.2015.03.016 (2015).

9 Lister, R. *et al.* Human DNA methylomes at base resolution show widespread epigenomic differences. *Nature* **462**, 315-322, doi:10.1038/nature08514 (2009).

10 Bueno, C. *et al.* Enhanced hemato-endothelial specification during human embryonic differentiation through developmental cooperation between AF4-MLL and MLL-AF4 fusions. *Haematologica* **104**, 1189-1201, doi:10.3324/haematol.2018.202044 (2019).

11 Love, M. I., Huber, W. & Anders, S. Moderated estimation of fold change and dispersion for RNA-seq data with DESeq2. *Genome Biol* **15**, 550, doi:10.1186/s13059-014-0550-8 (2014).

12 Ramirez, F., Dundar, F., Diehl, S., Gruning, B. A. & Manke, T. deepTools: a flexible platform for exploring deep-sequencing data. *Nucleic Acids Res* **42**, W187-191, doi:10.1093/nar/gku365 (2014).

13 Quinlan, A. R. & Hall, I. M. BEDTools: a flexible suite of utilities for comparing genomic features. *Bioinformatics* **26**, 841-842, doi:10.1093/bioinformatics/btq033 (2010).
14 Li, H. *et al.* The Sequence Alignment/Map format and SAMtools. *Bioinformatics* **25**, 2078-2079, doi:10.1093/bioinformatics/btp352 (2009).

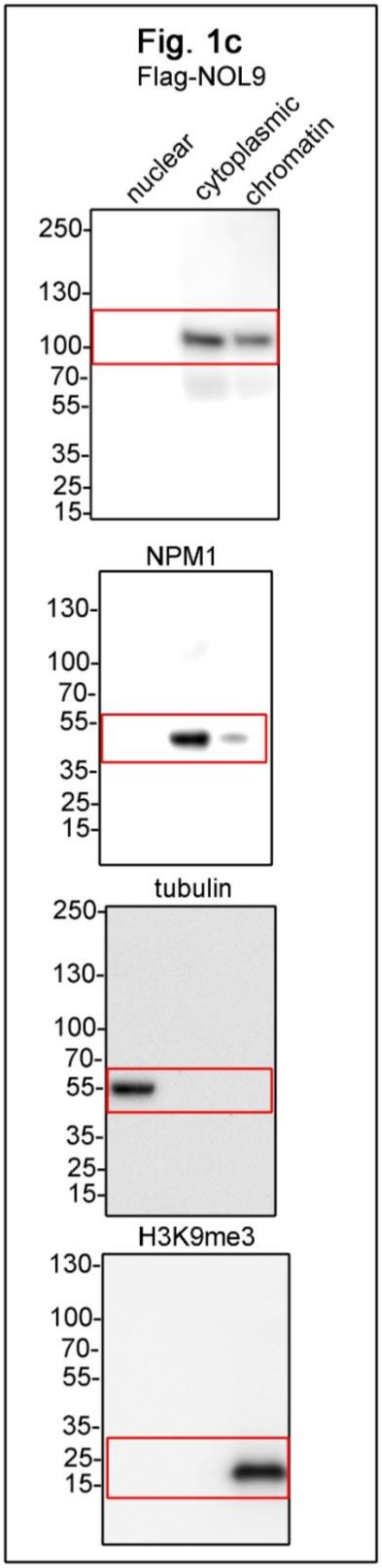
Fig. 1c
Flag-NOL9
nuclear
cytoplasmic
chromatin
250-
130-
100-
70-
55-
35-
25-
15-
NPM1
tubulin
H3K9me3

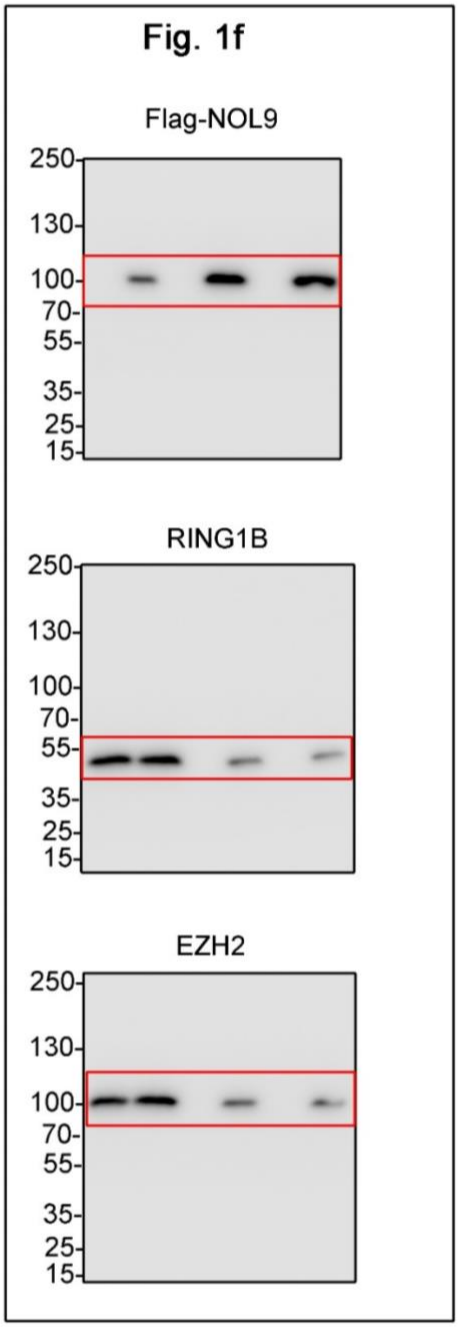
Fig. 1f
Flag-NOL9
RING1B
EZH2
250-
130-
100-
70-
55-
35-
25-
15-

Fig. 1g

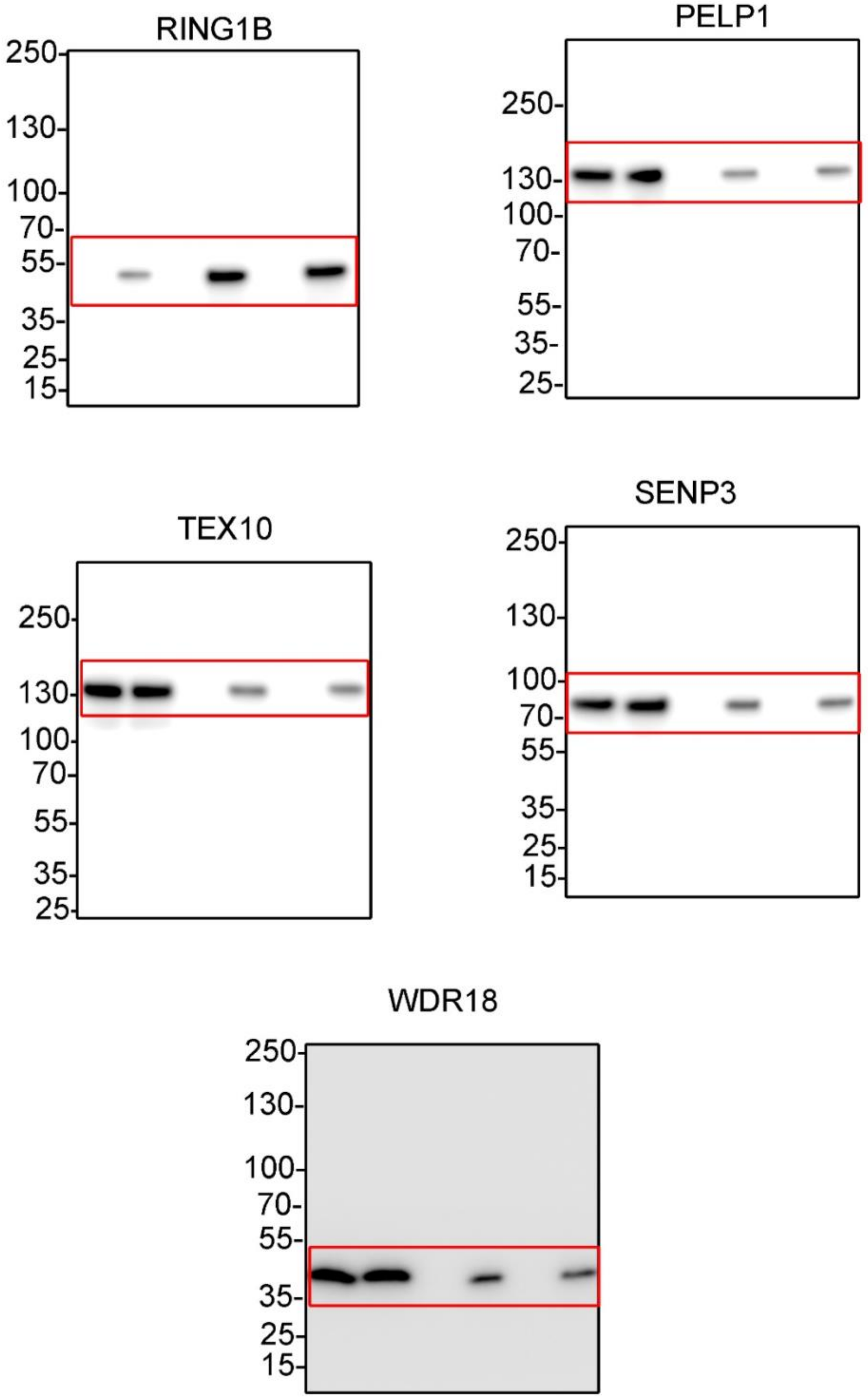
RING1B
250-
130-
100-
70-
55-
35-
25-
15-
PELP1
250-
130-
100-
70-
55-
35-
25-
TEX10
250-
130-
100-
70-
55-
35-
25-
SENP3
250-
130-
100-
70-
55-
35-
25-
15-
WDR18
250-
130-
100-
70-
55-
35-
25-
15-

Fig. 3b

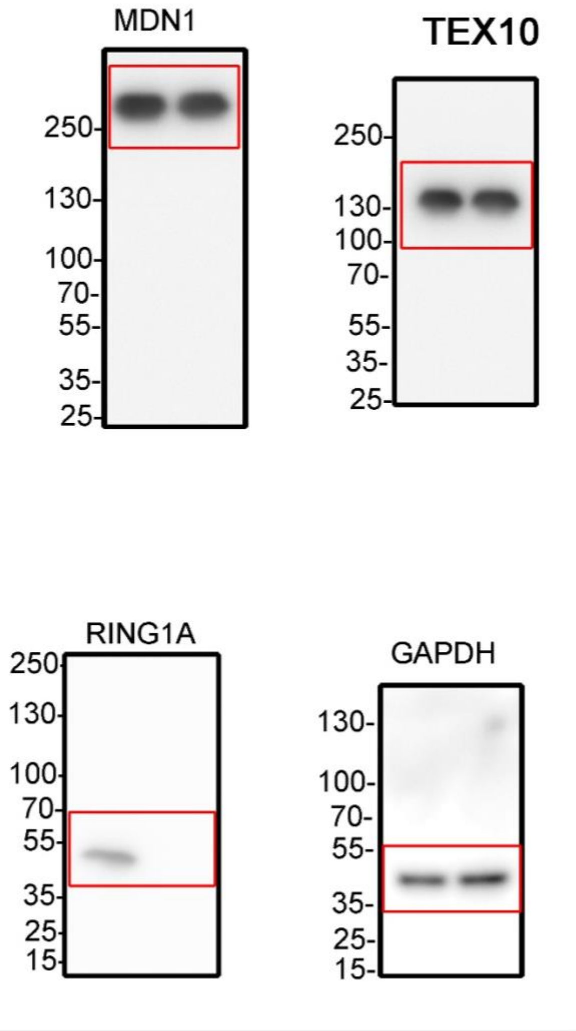
MDN1
250-
130-
100-
70-
55-
35-
25-
TEX10
250-
130-
100-
70-
55-
35-
25-
RING1A
250
130
100
70
55
35
25
15
GAPDH
130-
100-
70-
55-
35-
25-
15-

Fig. 3g

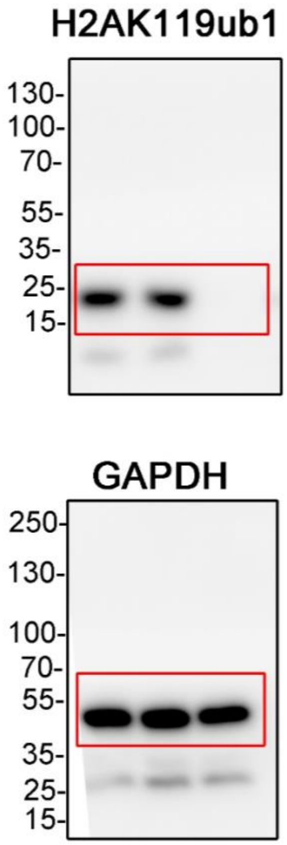
H2AK119ub1
130-
100-
70-
55-
35-
25-
15-
GAPDH
250-
130-
100-
70-
55-
35-
25-
15-

Fig. 3e

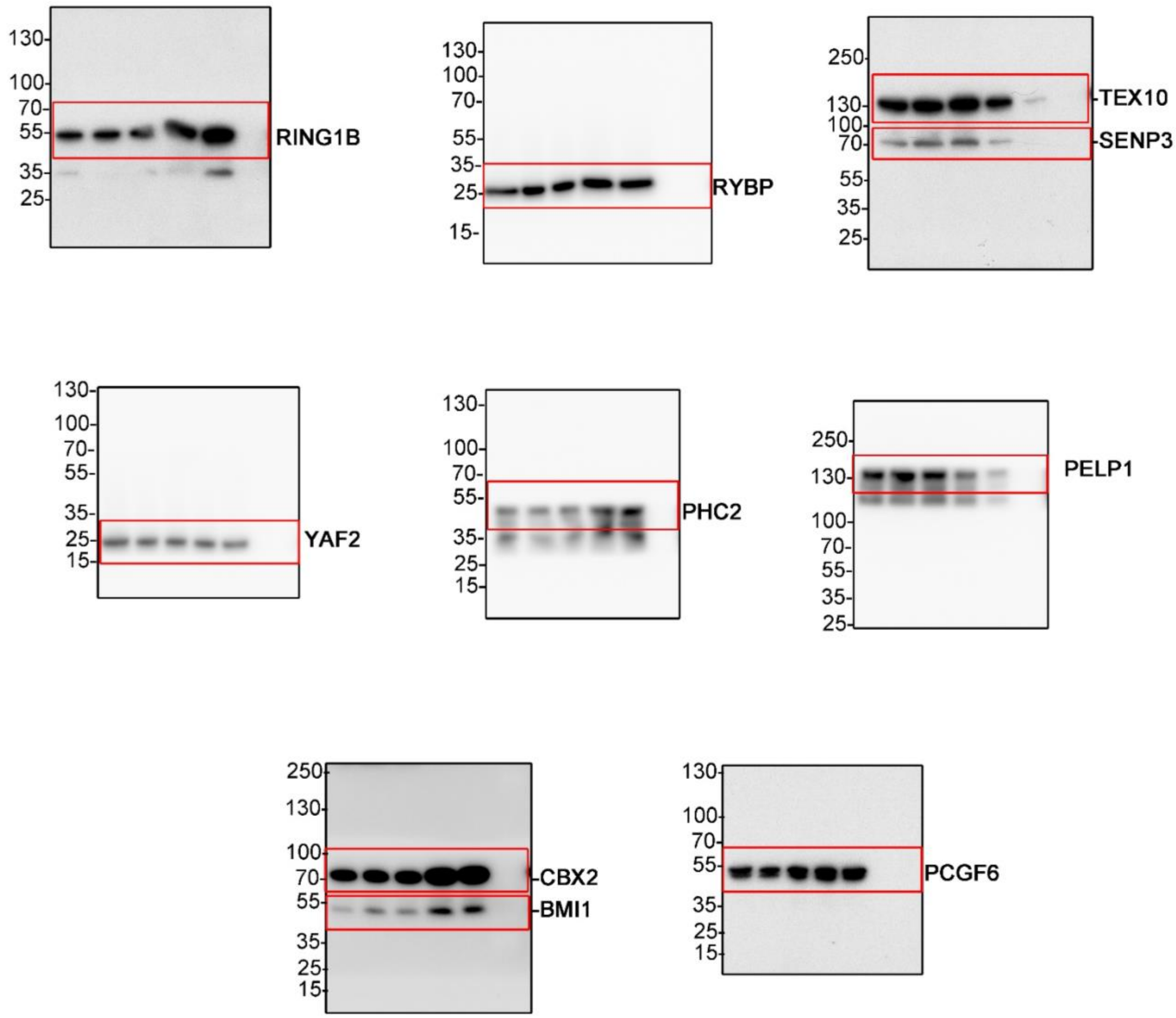

130
100
70
55
35
25
RING1B
130
100
70
55
35
25
15
RYBP
250
130
100
70
55
35
25
TEX10
SENP3
130
100
70
55
35
25
15
YAF2
130
100
70
55
35
25
15
PHC2
250
130
100
70
55
35
25
PELP1
250
130
100
70
55
35
25
15
CBX2
BMI1
130
100
70
55
35
25
15
PCGF6

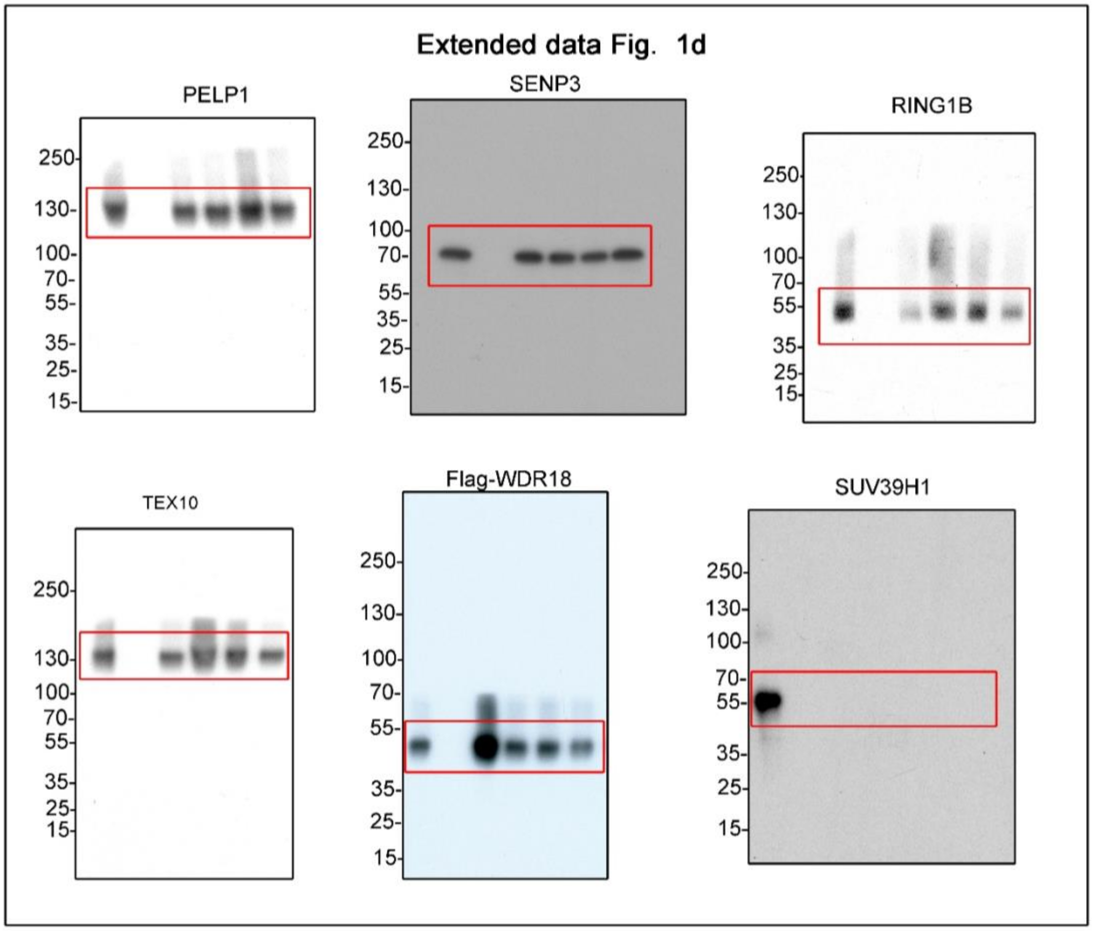
Extended data Fig. 1d
PELP1
250-
130-
100-
70-
55-
35-
25-
15-
SENP3
250-
130-
100-
70-
55-
35-
25-
15-
RING1B
250
130
100
70
55
35
25
15
TEX10
250-
130-
100-
70-
55-
35-
25-
15-
Flag-WDR18
250-
130-
100-
70-
55-
35-
25-
15-
SUV39H1
250-
130-
100-
70-
55-
35-
25-
15-

Extended data Fig. 1h

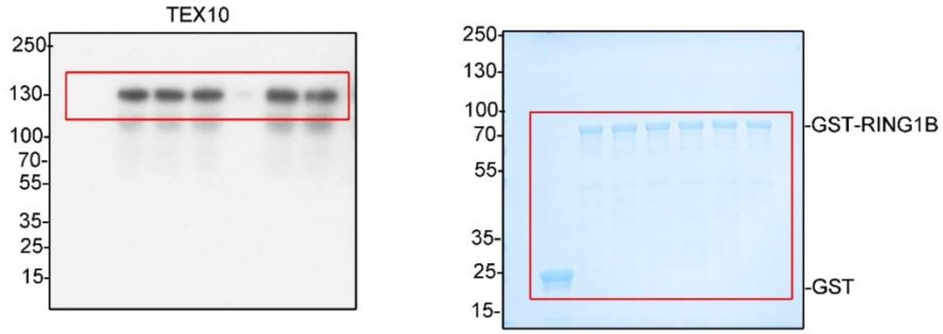

Extended data Fig. 1i

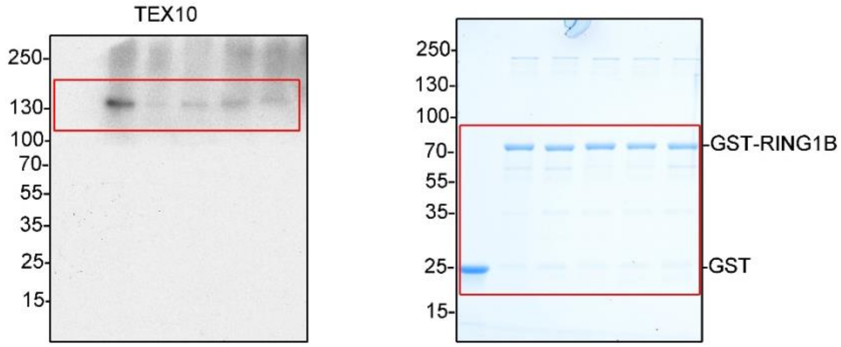

Extended data Fig. 2a

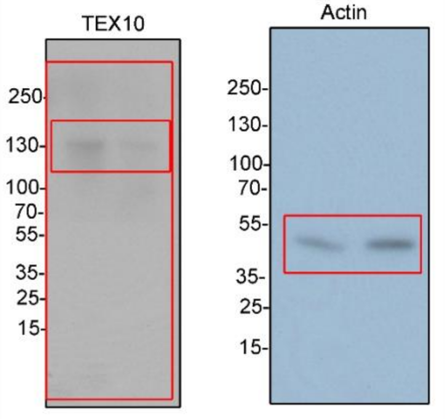

Extended data Fig. 2b

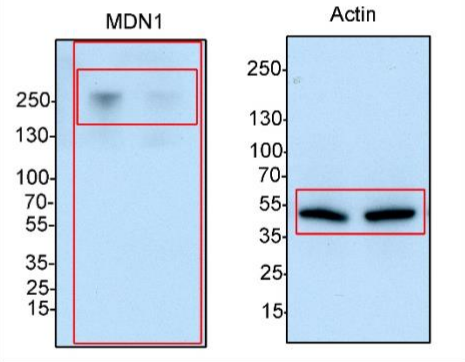

Extended data Fig. 3a

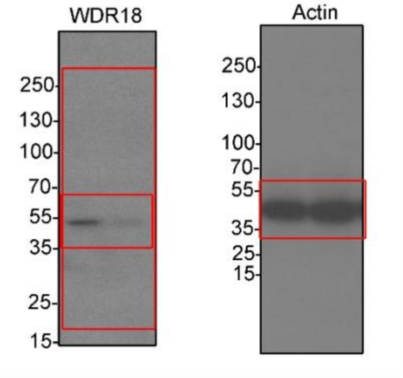

Extended data Fig. 3b

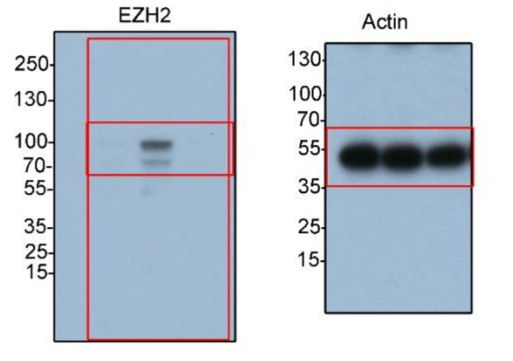

Extended data Fig. 3c

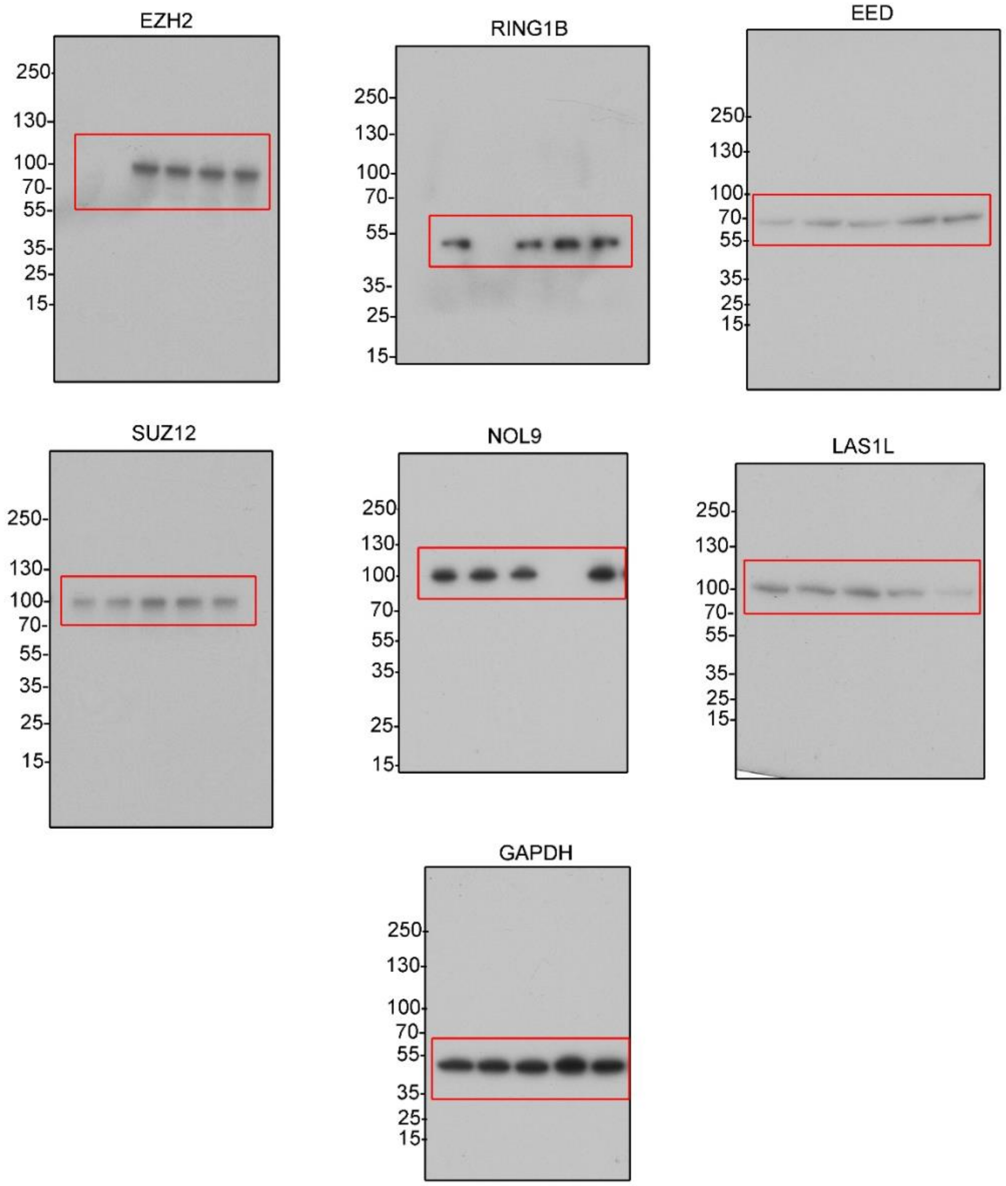

Extended data Fig. 5a RING1B-2A

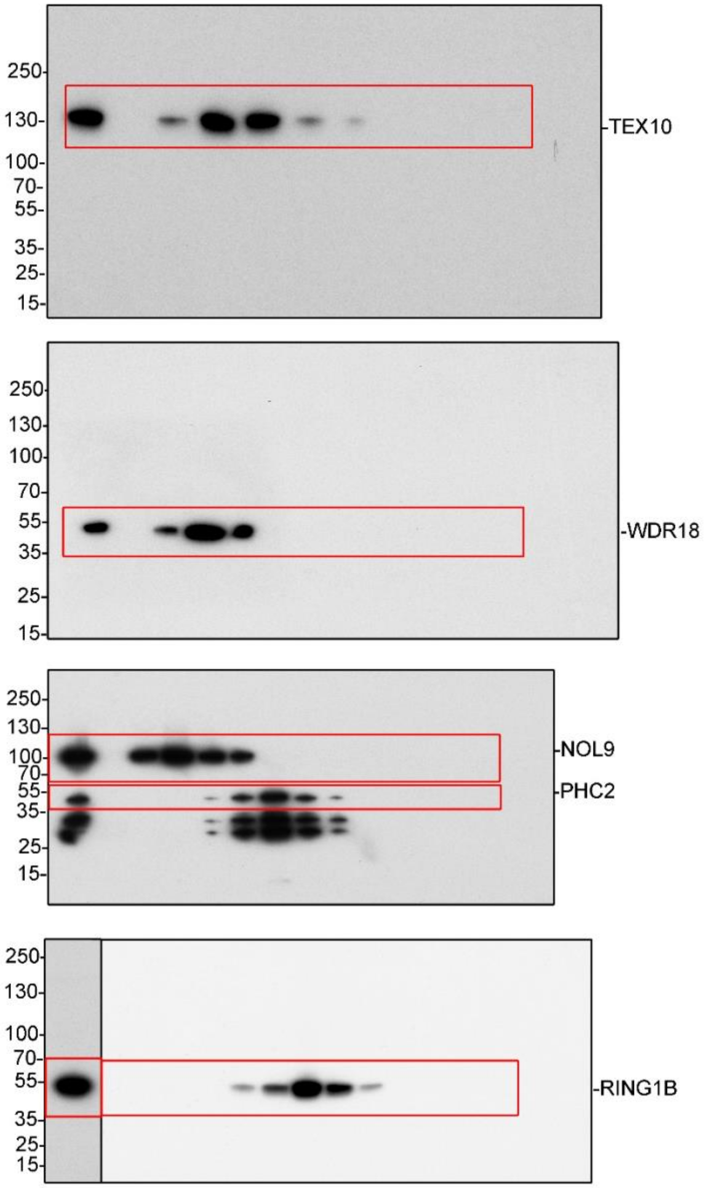

Extended data Fig. 5a WT

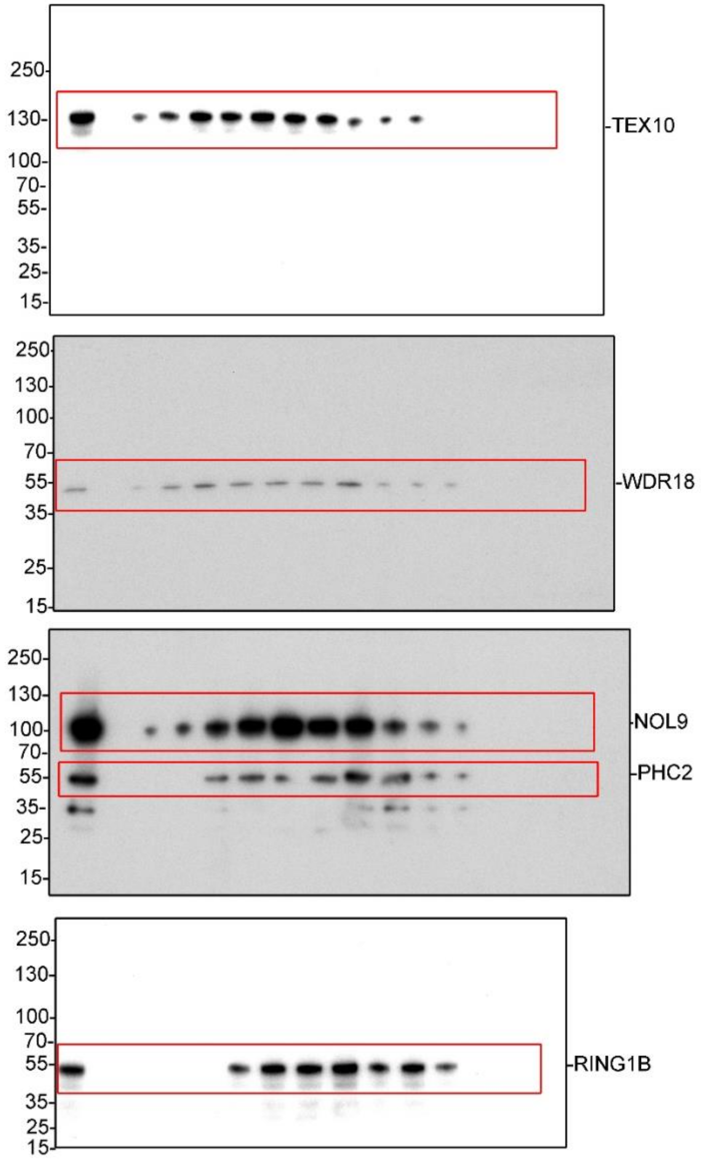

Extended data Fig. 10a

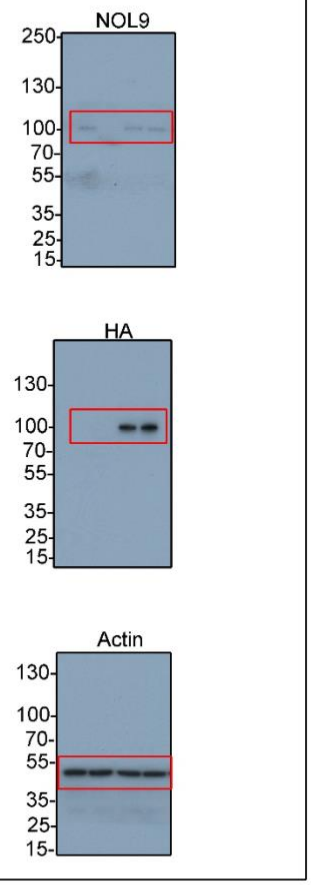

Extended data Fig. 10b

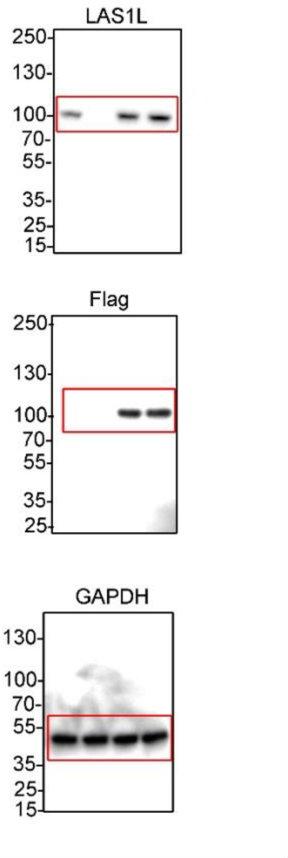

Extended data Fig. 10c

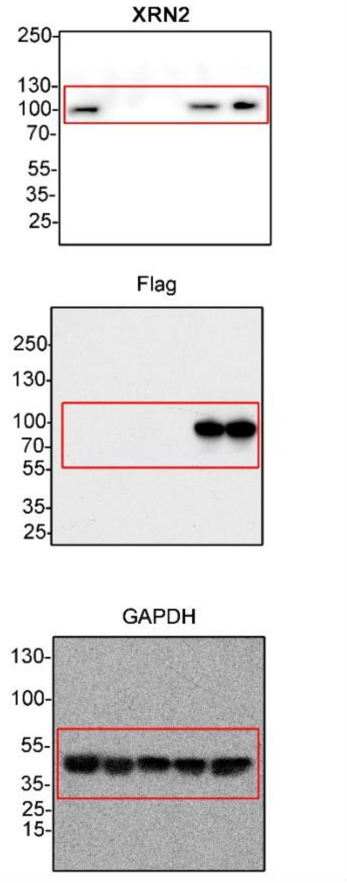

Supplement: Supplementary file 1 [file 41586_2022_4598_MOESM1_ESM.pdf]
