# Supplementary material for: Rixosomal RNA degradation contributes to silencing of Polycomb target genes
Source: Nature. 2022 Mar 30;604(7904):167–74. doi: 10.1038/s41586-022-04598-0 (PMC8986528; doi:10.1038/s41586-022-04598-0)
Supplement: Supplementary file 2 [file 41586_2022_4598_MOESM2_ESM.pdf]

Corresponding author(s): Danesh Moazed

Last updated by author(s): Jan 19, 2022

Reporting Summary

Nature Portfolio wishes to improve the reproducibility of the work that we publish. This form provides structure for consistency and transparency in reporting. For further information on Nature Portfolio policies, see our Editorial Policies and the Editorial Policy Checklist.

Statistics

For all statistical analyses, confirm that the following items are present in the figure legend, table legend, main text, or Methods section.

n/a	Confirmed	
☐	☒	The exact sample size (*n*) for each experimental group/condition, given as a discrete number and unit of measurement
☐	☒	A statement on whether measurements were taken from distinct samples or whether the same sample was measured repeatedly
☐	☒	The statistical test(s) used AND whether they are one- or two-sided<br>*Only common tests should be described solely by name; describe more complex techniques in the Methods section.*
☒	☐	A description of all covariates tested
☒	☐	A description of any assumptions or corrections, such as tests of normality and adjustment for multiple comparisons
☐	☒	A full description of the statistical parameters including central tendency (e.g. means) or other basic estimates (e.g. regression coefficient) AND variation (e.g. standard deviation) or associated estimates of uncertainty (e.g. confidence intervals)
☐	☒	For null hypothesis testing, the test statistic (e.g. *F*, *t*, *r*) with confidence intervals, effect sizes, degrees of freedom and *P* value noted<br>*Give P values as exact values whenever suitable.*
☒	☐	For Bayesian analysis, information on the choice of priors and Markov chain Monte Carlo settings
☒	☐	For hierarchical and complex designs, identification of the appropriate level for tests and full reporting of outcomes
☒	☐	Estimates of effect sizes (e.g. Cohen's *d*, Pearson's *r*), indicating how they were calculated

Our web collection on statistics for biologists contains articles on many of the points above.

Software and code

Policy information about availability of computer code

Data collection	NIS-Elements imaging software (version 4.11.0) was used for imaging data collection.
Data analysis	DEseq2 (v1.18.1), deeptool (v3.0.2), Bedtool (v2.27.1), Samtool (v1.3.1)

For manuscripts utilizing custom algorithms or software that are central to the research but not yet described in published literature, software must be made available to editors and reviewers. We strongly encourage code deposition in a community repository (e.g. GitHub). See the Nature Portfolio guidelines for submitting code & software for further information.

Data

Policy information about availability of data

All manuscripts must include a data availability statement. This statement should provide the following information, where applicable:

- Accession codes, unique identifiers, or web links for publicly available datasets
- A description of any restrictions on data availability
- For clinical datasets or third party data, please ensure that the statement adheres to our policy

The siRNAs used in this study are listed in Extended Table S1. The primers used for ChIP-qPCR and RT-qPCR are listed in Extended Table S4. The next-generation sequencing data sets generated from this study are deposited in Gene Expression Omnibus (GEO) database using accession ID GSE175678, including ChIP-seq, RNA-seq, and PRO-seq. Published ChIP-seq datasets used in this study were downloaded from NCBI. All the datasets accession numbers are listed in Extended Table S5. The Mass spectrometry protein lists are available in Extended Tables S7 and S8.

Field-specific reporting

Please select the one below that is the best fit for your research. If you are not sure, read the appropriate sections before making your selection.

☒ Life sciences ☐ Behavioural & social sciences ☐ Ecological, evolutionary & environmental sciences

For a reference copy of the document with all sections, see nature.com/documents/nr-reporting-summary-flat.pdf

Life sciences study design

All studies must disclose on these points even when the disclosure is negative.

Sample size	Sample size was estimated according to preliminary experiments and the size of the observed effect.<br>For all high-throughput sequencing experiments, two independent biological replicates were used (RNA-seq, ChIP-seq, and PRO-seq). Two biological replicates allow us to calculate the p value and FDR values in RNAs-seq and PRO-seq analysis.<br>ChIP-qPCR and RT-qPCR were were repeated with 2-4 biological replicates.<br>For counting foci in IF experiments, n>20 cells were counted for each cell staining.
Data exclusions	No data was excluded from the analysis.
Replication	Each experiment was repeated (See Figure Legends). For all high-throughput sequencing experiments, there are two biological replicates. For ChIP-qPCR and RT-qPCR, there are three biological replicates. For immunostaining, there are three biological replicates. For immunocrecipitaition, three are two biological replicates.
Randomization	Samples were allocated to groups according to genotype or treatment. No randomization was required as the starting materials.
Blinding	The investigators were not blinded to allocation during experiments and outcome assessment. Blinding was not required because the results of physical measurements of biomolecules, phenotypic analysis (e.g., growth rates), or sequencing of nucleic acid libraries are not affected by the experimenters knowledge of sample identities. For all assays there are both negative controls and positive controls and all the results are obtained in parallel using the same settings, and each treatment was assigned to a number during the experiment.

Reporting for specific materials, systems and methods

We require information from authors about some types of materials, experimental systems and methods used in many studies. Here, indicate whether each material, system or method listed is relevant to your study. If you are not sure if a list item applies to your research, read the appropriate section before selecting a response.

Materials & experimental systems

n/a	Involved in the study	
☐	☒	Antibodies
☐	☒	Eukaryotic cell lines
☒	☐	Palaeontology and archaeology
☒	☐	Animals and other organisms
☒	☐	Human research participants
☒	☐	Clinical data
☒	☐	Dual use research of concern

Methods

n/a	Involved in the study	
☐	☒	ChIP-seq
☒	☐	Flow cytometry
☒	☐	MRI-based neuroimaging

Antibodies

Antibodies used	All antibodies used have been provided in Extended Data Table 3 with details
Validation	"1. Flag. The manufacturer indicates reactivity by immunoblot to detect Flag epitope-tagged proteins. This antibody has been intensely used and proved to perform well in immunoprecipitation experimemnts. We validated it in western blot with siRNA knockdown in human HEK293FT cells as well as against untagged control cells. We also validated it in IP-Mass spectrometry experiments by comparing IP results in Flag-NOL9 and wild type (untagged) in human HEK293FT cells.<br>2. EZH2. We validated this antibody with siRNA knockdown in western blotting and immunofluorescence in human HEK293FT cells.<br>3. MDN1. We validated this antibody with siRNA knockdown in western blotting, ChIP-seq and immunofluorescence in human HEK293FT cells.<br>4. GAPDH: The manufacturer indicates human and mouse reactivity by immunoblot. It is widely used in western blot as a loading control. We validated it in HEK293FT cells.<br>5. TEX10. We validated this antibody with siRNA knockdown in western blotting and ChIP-seq in human HEK293FT cells and human ES cells.<br>6. WDR18. We validated this antibody with siRNA knockdown in western blotting and immunofluorescence in human HEK293FT cells.<br>7. NOL9. We validated this antibody with siRNA knockdown in western blotting in human HEK293FT cells.

8. H2AK119ub1. We validated this antibody with RING1A/B double knockout in western blotting in human HEK293FT cells.
9. H3K27me3. This is widely used in the field. We validated in human HEK293FT cells.
10. LAS1L. We validated this antibody with siRNA knockdown in western blotting in human HEK293FT cells.
11. PELP1. We validated this antibody with siRNA knockdown in western blotting in human HEK293FT cells.
12. SUV39H1. We validated this antibody with siRNA knockdown in western blotting in human HEK293FT cells.
13. CBX2. We validated this antibody in western blotting in human HEK293FT cells.
14. PHC2. We validated this antibody in western blotting in human HEK293FT cells.
15. Actin. We validated this antibody in western blotting in human HEK293FT cells.
16. H3K9me3. We validated this antibody in western blotting in human HEK293FT cells.
17. XRN2. We validated this antibody in western blotting in human HEK293FT cells.
18. EED. We validated this antibody in western blotting in human HEK293FT cells.
19. SUZ12. We validated this antibody in western blotting in human HEK293FT cells.
20. NPM1. We validated this antibody with siRNA knockdown in western blotting and immunofluorescence in human HEK293FT cells.
21. BMI1. We validated this antibody in western blotting in human HEK293FT cells.
22. SENP3. We validated this antibody in western blotting in human HEK293FT cells.
23. RYBP: We validated this antibody in western blotting in human HEK293FT cells.
24. YAF2: We validated this antibody in western blotting in human HEK293FT cells.
25. PCGF6: We validated this antibody in western blotting in human HEK293FT cells.

Eukaryotic cell lines

Policy information about cell lines

Cell line source(s)

HEK293FT, from ThermoFisher (R70007). human ES cells, from Cell biology department at Harvard Medical School. HeLa, from ATCC (CCL-2).

Authentication

For cells purchased directly from ATCC, cells are authenticated by sequencing at ATCC. For human ES cells, cell culture is conducted by stem cell facillity from HMS cell biology department. During culture, cells were authenticated based on the testing and monitoring of phenotypic features (morphology, differentiation potential, growth conditions, etc.).

Mycoplasma contamination

All cell lines tested negative for Mycoplasma contamination.

Commonly misidentified lines
(See ICLAC register)

No misidentidied cell lines were used.

ChIP-seq

Data deposition

☒ Confirm that both raw and final processed data have been deposited in a public database such as GEO.

☒ Confirm that you have deposited or provided access to graph files (e.g. BED files) for the called peaks.

Data access links
May remain private before publication.

GSE175678:
HTTP://www.ncbi.nlm.nih.gov/geo/query/acc.cgi?acc=GSE175678

Files in database submission

"INPUT_siMDN1_rep1.bw
INPUT_siMDN1_rep2.bw
INPUT_siNC_rep1.bw
INPUT_siNC_rep2.bw
INPUT_siNOL9_rep1.bw
INPUT_siNOL9_rep2.bw
H3K9me3_rep1.bw
H3K9me3_rep2.bw
H3K27me3_siNC_rep1.bw
H3K27me3_siNC_rep2.bw
H3K27me3_siNOL9_rep1.bw
H3K27me3_siNOL9_rep2.bw
MDN1_siMDN1_rep1.bw
MDN1_siMDN1_rep2.bw
MDN1_siNC_rep1.bw
MDN1_siNC_rep2.bw
H2AK119ub1-HEK293FT_rep1.bw
H2AK119ub1-HEK293FT_rep2.bw
H2AK119ub1-HEK293FT_siNOL9_rep1.bw
H2AK119ub1-HEK293FT_siNOL9_rep2.bw
MDN1-HEK293FT_RING1DKO_rep1.bw
MDN1-HEK293FT_RING1DKO_rep2.bw
TEX10-HEK293FT_siNC_rep1.bw
TEX10-HEK293FT_siNC_rep2.bw
TEX10-HEK293FT_siTEX10_rep1.bw
TEX10-HEK293FT_siTEX10_rep2.bw
TEX10-HEK293FT_RING1DKO_rep1.bw

TEX10-HEK293FT_RING1DKO_rep2.bw
H2AK119ub1-HeLa_rep1.bw
H2AK119ub1-HeLa_rep2.bw
H3K27me3-HeLa_rep1.bw
H3K27me3-HeLa_rep2.bw
INPUT-HeLa_rep1.bw
INPUT-HeLa_rep2.bw
MDN1-HeLa_siMDN1_rep1.bw
MDN1-HeLa_siMDN1_rep2.bw
MDN1-HeLa_siNC_rep1.bw
MDN1-HeLa_siNC_rep2.bw
H2AK119ub1-ES_rep1.bw
H2AK119ub1-ES_rep2.bw
INPUT-ES_rep1.bw
INPUT-ES_rep2.bw
TEX10-ES_rep1.bw
TEX10-ES_rep2.bw
TEX10-ES_siTEX10_rep1.bw
TEX10-ES_siTEX10_rep2.bw
H2AK119ub1_HEK293FT_RING1BQ137AQ138A_rep1.bw
H2AK119ub1_HEK293FT_RING1BQ137AQ138A_rep2.bw
H2AK119ub1_HEK293FT_WT_rep1.bw
H2AK119ub1_HEK293FT_WT_rep2.bw
RING1B_HEK293FT_WT_rep1.bw
RING1B_HEK293FT_WT_rep2.bw
RING1B_HEK293FT_RING1BQ137AQ138A_rep1.bw
RING1B_HEK293FT_RING1BQ137AQ138A_rep2.bw

INPUT_siMDN1_rep1.fq
INPUT_siMDN1_rep2.fq
INPUT_siNC_rep1.fq
INPUT_siNC_rep2.fq
INPUT_siNOL9_rep1.fq
INPUT_siNOL9_rep2.fq
H3K9me3_rep1.fq
H3K9me3_rep2.fq
H3K27me3_siNC_rep1.fq
H3K27me3_siNC_rep2.fq
H3K27me3_siNOL9_rep1.fq
H3K27me3_siNOL9_rep2.fq
MDN1_siMDN1_rep1.fq
MDN1_siMDN1_rep2.fq
MDN1_siNC_rep1.fq
MDN1_siNC_rep2.fq
H2AK119ub1-HEK293FT_rep1.fq
H2AK119ub1-HEK293FT_rep2.fq
H2AK119ub1-HEK293FT_siNOL9_rep1.fq
H2AK119ub1-HEK293FT_siNOL9_rep2.fq
MDN1-HEK293FT_RING1DKO_rep1.fq
MDN1-HEK293FT_RING1DKO_rep2.fq
TEX10-HEK293FT_siNC_rep1.fq
TEX10-HEK293FT_siNC_rep2.fq
TEX10-HEK293FT_siTEX10_rep1.fq
TEX10-HEK293FT_siTEX10_rep2.fq
TEX10-HEK293FT_RING1DKO_rep1.fq
TEX10-HEK293FT_RING1DKO_rep2.fq
H2AK119ub1-HeLa_rep1.fq
H2AK119ub1-HeLa_rep2.fq
H3K27me3-HeLa_rep1.fq
H3K27me3-HeLa_rep2.fq
INPUT-HeLa_rep1.fq
INPUT-HeLa_rep2.fq
MDN1-HeLa_siMDN1_rep1.fq
MDN1-HeLa_siMDN1_rep2.fq
MDN1-HeLa_siNC_rep1.fq
MDN1-HeLa_siNC_rep2.fq
H2AK119ub1-ES_rep1.fq
H2AK119ub1-ES_rep2.fq
INPUT-ES_rep1.fq
INPUT-ES_rep2.fq
TEX10-ES_rep1.fq
TEX10-ES_rep2.fq
TEX10-ES_siTEX10_rep1.fq
TEX10-ES_siTEX10_rep2.fq
H2AK119ub1_HEK293FT_RING1BQ137AQ138A_rep1.fq

H2AK119ub1_HEK293FT_RING1BQ137AQ138A_rep2.fq
H2AK119ub1_HEK293FT_WT_rep1.fq
H2AK119ub1_HEK293FT_WT_rep2.fq
RING1B_HEK293FT_WT_rep1.fq
RING1B_HEK293FT_WT_rep2.fq
RING1B_HEK293FT_RING1BQ137AQ138A_rep1.fq
RING1B_HEK293FT_RING1BQ137AQ138A_rep2.fq

"

Genome browser session
(e.g. UCSC)

https://genome.ucsc.edu/cgi-bin/hgTracks?db=hg19&lastVirtModeType=default&lastVirtModeExtraState=&virtModeType=default&virtMode=0&nonVirtPosition=&position=chr7%3A27069735%2D27331434&hgsid=1124281605_98AtDsDojdyO66v1aVSLPv1CFn1W

Methodology

Replicates

All ChIP-seq experiments are two biological replicates.
Key target genes were validated by ChIP-qPCR with three biological replicates.
All the Spearson correlations between two replicates are > 0.9.

Sequencing depth

"All the ChIP-seq reads are 50 bp single-ended.
sample name, accession, reads, mappable reads
H3K9me3_rep1 GSM4239943 36,178,114 33,299,264
H3K9me3_rep2 GSM4239944 33,710,338 30,088,342
H3K27me3_siNC_rep1 GSM4239945 22,448,370 19,986,885
H3K27me3_siNC_rep2 GSM4239946 27,258,302 25,489,190
H3K27me3_siNOL9_rep1 GSM4239947 25,236,224 22,201,241
H3K27me3_siNOL9_rep2 GSM4239948 44,112,820 32,274,856
MDN1_siMDN1_rep1 GSM4239949 28,876,349 26,794,150
MDN1_siMDN1_rep2 GSM4239950 24,505,119 21,252,219
MDN1_siNC_rep1 GSM4239951 47,455,840 39,841,271
MDN1_siNC_rep2 GSM4239952 46,749,114 39,523,629
H2AK119ub1-HEK293FT_rep1 GSM4502558 42,814,400 38,796,292
H2AK119ub1-HEK293FT_rep2 GSM4502559 44,810,575 40,851,022
H2AK119ub1-HEK293FT_siNOL9_rep1 GSM5343681 30831920 28979586
H2AK119ub1-HEK293FT_siNOL9_rep2 GSM5343682 28673743 26359794
MDN1-HEK293FT_RING1DKO_rep1 GSM5343683 51892351 30157140
MDN1-HEK293FT_RING1DKO_rep2 GSM5343684 68970398 23819904
TEX10-HEK293FT_siNC_rep1 GSM5343685 52349542 28440006
TEX10-HEK293FT_siNC_rep2 GSM5343686 45222611 26067372
TEX10-HEK293FT_siTEX10_rep1 GSM5343687 54472229 45170712
TEX10-HEK293FT_siTEX10_rep2 GSM5343688 53020996 43660440
TEX10-HEK293FT_RING1DKO_rep1 GSM5343689 43922816 38410092
TEX10-HEK293FT_RING1DKO_rep2 GSM5343690 37940504 33263796
H2AK119ub1-HeLa_rep1 GSM5343691 22133412 20431728
H2AK119ub1-HeLa_rep2 GSM5343692 21543900 18632208
H3K27me3-HeLa_rep1 GSM5343693 23139858 17712162
H3K27me3-HeLa_rep2 GSM5343694 22330217 16491690
INPUT-HeLa_rep1 GSM5343695 29677221 23185104
INPUT-HeLa_rep2 GSM5343696 28716807 21996510
MDN1-HeLa_siMDN1_rep1 GSM5343697 26077688 18961200
MDN1-HeLa_siMDN1_rep2 GSM5343698 25770612 17774814
MDN1-HeLa_siNC_rep1 GSM5343699 28612815 25861200
MDN1-HeLa_siNC_rep2 GSM5343700 27897975 24603468
H2AK119ub1-ES_rep1 GSM5343673 38266571 31901874
H2AK119ub1-ES_rep2 GSM5343674 39721402 32829510
INPUT-ES_rep1 GSM5343675 65906239 41147184
INPUT-ES_rep2 GSM5343676 58572325 40123776
TEX10-ES_rep1 GSM5343677 41964069 35945550
TEX10-ES_rep2 GSM5343678 37781268 33029886
TEX10-ES_siTEX10_rep1 GSM5343679 46161213 40625820
TEX10-ES_siTEX10_rep2 GSM5343680 63131985 38097246
INPUT_siMDN1_rep1 GSM4239937 28980392 22973640
INPUT_siMDN1_rep2 GSM4239938 33121564 27500160
INPUT_siNC_rep1 GSM4239939 21738787 19575000
INPUT_siNC_rep2 GSM4239940 46815083 28853400
INPUT_siNOL9_rep1 GSM4239941 26745670 22704720
INPUT_siNOL9_rep2 GSM4239942 38271800 30207360
H2AK119ub1_HEK293FT_RING1BQ137AQ138A_rep1 GSM5659334 35987406 27997560
H2AK119ub1_HEK293FT_RING1BQ137AQ138A_rep2 GSM5659335 33736059 26545080
H2AK119ub1_HEK293FT_WT_rep1 GSM5659336 35744555 27872520
H2AK119ub1_HEK293FT_WT_rep2 GSM5659337 35313796 27292680
RING1B_HEK293FT_WT_rep1 GSM5659338 51366994 32583120
RING1B_HEK293FT_WT_rep2 GSM5659339 48056785 31293360

RING1B_HEK293FT_RING1BQ137AQ138A_rep1 GSM5659340 50382269 34448640
RING1B_HEK293FT_RING1BQ137AQ138A_rep2 GSM5659341 70536347 45870480"

Antibodies

"MDN1, Bethyl, A304-739A-T.
TEX10, Thermofisher, 720257.
H3K27me3, Millipore, 17-622.
H2AK119ub1, CST, 8240T.
H3K9me3, Diagenode, C15500003-50.
RING1B, CST, 5694S"

Peak calling parameters

" macs2/2.1.1.20160309.
macs2 callpeak -t sample.bam -c input.bam -f BAM -g hs --nomodel --broad -p 1e-9 --broad-cutoff 0.05"

Data quality

FASTQC 0.11.5 is run to check the sequencing quality.

Software

DEseq2 (v1.18.1), deeptool (v3.0.2), Bedtool (v2.27.1), Samtool (v1.3.1)
